# Risk factors for severe COVID-19 outcomes in the Asia-Pacific region: a literature review

**DOI:** 10.3389/fpubh.2025.1562179

**Published:** 2025-06-09

**Authors:** Madeline Thompson, Amanda K. Buttery, Shu Xin Oh, Macy Chan, Byung Hyun Lee, Tomoharu Iino, Yu-Chun Alice Wang, Chris Clarke

**Affiliations:** ^1^Moderna, Melbourne, VIC, Australia; ^2^Moderna, Singapore, Singapore; ^3^Moderna, Hong Kong, Hong Kong SAR, China; ^4^Moderna, Seoul, Republic of Korea; ^5^Moderna, Tokyo, Japan; ^6^Moderna, Taipei, Taiwan

**Keywords:** COVID-19, severe outcomes, mortality, Asia-Pacific, risk factors, SARS-CoV-2, comorbidity, epidemiology

## Abstract

This comprehensive synthesis of severe COVID-19 risk factors specific to the Asia-Pacific (APAC) region addresses gaps in previous global studies, which often overlook regional demographic, epidemiological, and healthcare system variations. Three databases (PubMed, Ovid MedLine, Scopus) and two preprint platforms (BioRxiv, MedRxiv) were searched between December 1, 2019, and March 31, 2023. English-language publications from 11 APAC countries/regions (Australia, Hong Kong, Japan, Macau, New Zealand, Philippines, Singapore, South Korea, Taiwan, Thailand and Vietnam) reporting conditions associated with severe COVID-19 outcomes in adults (aged ≥16 years) were included. Of 295 publications screened, 123 met inclusion criteria, mostly from South Korea (*n* = 68) and Japan (*n* = 23). Common risk factors included older age, male sex, obesity, diabetes, heart failure, renal disease, and dementia. Less commonly hypertension, chronic obstructive pulmonary disease, cardio-and cerebrovascular disease, immunocompromise, autoimmune disorders, and mental illness were reported. To date, no prior region-specific synthesis of risk factors for severe COVID-19 outcomes across the APAC region has been identified. The findings support the development of tailored vaccination strategies and public health interventions at both national and regional levels, helping ensure high-risk populations are prioritized in ongoing COVID-19 prevention and management efforts.

## Summary

- This review highlights risk factors for severe COVID-19 outcomes in APAC populations including age, immunocompromise, obesity, diabetes, heart failure, renal disease, dementia and mental illness.
- Understanding APAC-specific risk factors can support vaccination strategies and improve the targeting of high-risk groups.

## Introduction

SARS-CoV-2, the virus responsible for COVID-19, causes a spectrum of disease ranging from mild to severe, with a proportion of patients developing critical illness, particularly early in the pandemic (1, 2). Extensive research has been undertaken globally to identify demographics, lifestyle factors, and medical conditions that may increase the risk of severe COVID-19 outcomes (i.e., hospitalization, intensive care unit [ICU] admission, and death) (2, 3). The presence of preexisting medical conditions including hypertension, diabetes, cardiovascular disease (CVD), dementia, chronic respiratory disease, chronic kidney disease (CKD), cancer, and chronic liver disease are associated with an increased risk of COVID-19-related severe outcomes and mortality (4–6). Although numerous studies have investigated risk factors for severe COVID-19 outcomes globally, few have focused specifically on the Asia-Pacific (APAC) region. These studies often overlook ethnic and geographic differences, as well as social and healthcare variations, unique to the APAC region (7). For instance, elevated smoking rates among low-and-middle-income men in APAC countries contribute to region-specific incidence rates of CVD, stroke, and cancers (8, 9). Similarly, multimorbidity, defined as the presence of ≥2 conditions (10), is increasingly prevalent in Asia. A unique ‘lean diabetic’ phenotype (characterized by diabetes and low body mass index [BMI]), and evidence that Asian patients can experience heart failure (HF) up to a decade earlier than European and Northern American patients, may influence COVID-19 disease burden in the region (11).

Globally, COVID-19 vaccination has substantially improved clinical outcomes, as demonstrated by reductions in COVID-19–related hospitalizations, ICU admissions, and mortality (6, 12, 13). However, to optimize vaccination strategies in the APAC region, it is essential to understand the key medical risk factors that contribute to severe COVID-19 disease. Despite extensive global research, a critical gap remains in identifying and quantifying risk factors specific to APAC populations, where variations in demographics, disease burden and healthcare systems may influence outcomes. This review addresses this gap and contributes the following:

- Presents, to the best of current knowledge, the first synthesis of medical risk factors for severe COVID-19 outcomes focused specifically on the APAC region
- Synthesizes evidence in the context of region-specific health characteristics, such as earlier onset of chronic diseases and unique phenotypes like lean diabetes
- Highlights medical conditions that may be under-recognized in regional vaccination guidelines
- Identifies substantial heterogeneity across APAC studies in the definitions of risk conditions and outcomes, as well as methodological variability, underscoring the need for standardized approaches
- Informs more tailored, risk-based public health and vaccination strategies suited to the APAC context

## Methods

### Search strategy and eligibility criteria

A literature search was conducted across three electronic databases (PubMed, Ovid MedLine, and Scopus) and two preprint platforms (BioRxiv and MedRxiv) between December 1, 2019, and March 31, 2023. English-language publications from 11 APAC countries/regions (Australia, Hong Kong, Japan, Macau, New Zealand, Philippines, Singapore, South Korea, Taiwan, Thailand, and Vietnam) were included if they reported adjusted hazard ratios (aHRs) or odds ratios (aORs) for age, sex, and underlying medical conditions associated with severe COVID-19 outcomes. Studies were limited to research articles (cohort, cross-sectional, and case-controlled studies) of adults aged ≥16 years. Publications were also excluded if they contained pooled data where country- or region-level data could not be isolated and individually evaluated. Two independent reviewers (MT and CC) evaluated each study based on selection criteria, comparability, and outcome assessment. Discrepancies were resolved through discussion with a third reviewer (AKB).

### Data extraction and synthesis

Data were extracted from eligible studies on study design, setting, population characteristics, risk factors examined, comparator groups, and reported outcomes. Both positively and negatively associated findings were included, and results were grouped by risk factor and outcome (e.g., hospitalization, ICU admission, or mortality). A narrative synthesis was performed to summarize patterns across studies. Additional methodological detail is available in the Supplementary methods.

### Quality assessment of individual studies

Although not designed as a systematic review or meta-analysis, this review employed a robust and comprehensive approach to identify, screen, and extract data from the literature (Supplementary Table 17). Priority was given to larger cohort studies (*n* > 1,000) for common conditions. For less prevalent conditions (e.g., neurological or autoimmune diseases), studies with at least 50 participants were included if quantitative risk estimates were reported.

To mitigate potential publication bias, a known limitation of narrative reviews, the search strategy also included two preprint server databases. In addition, backward citation searches were conducted to enhance comprehensiveness.

## Results

### Literature search

**Figure 1:**
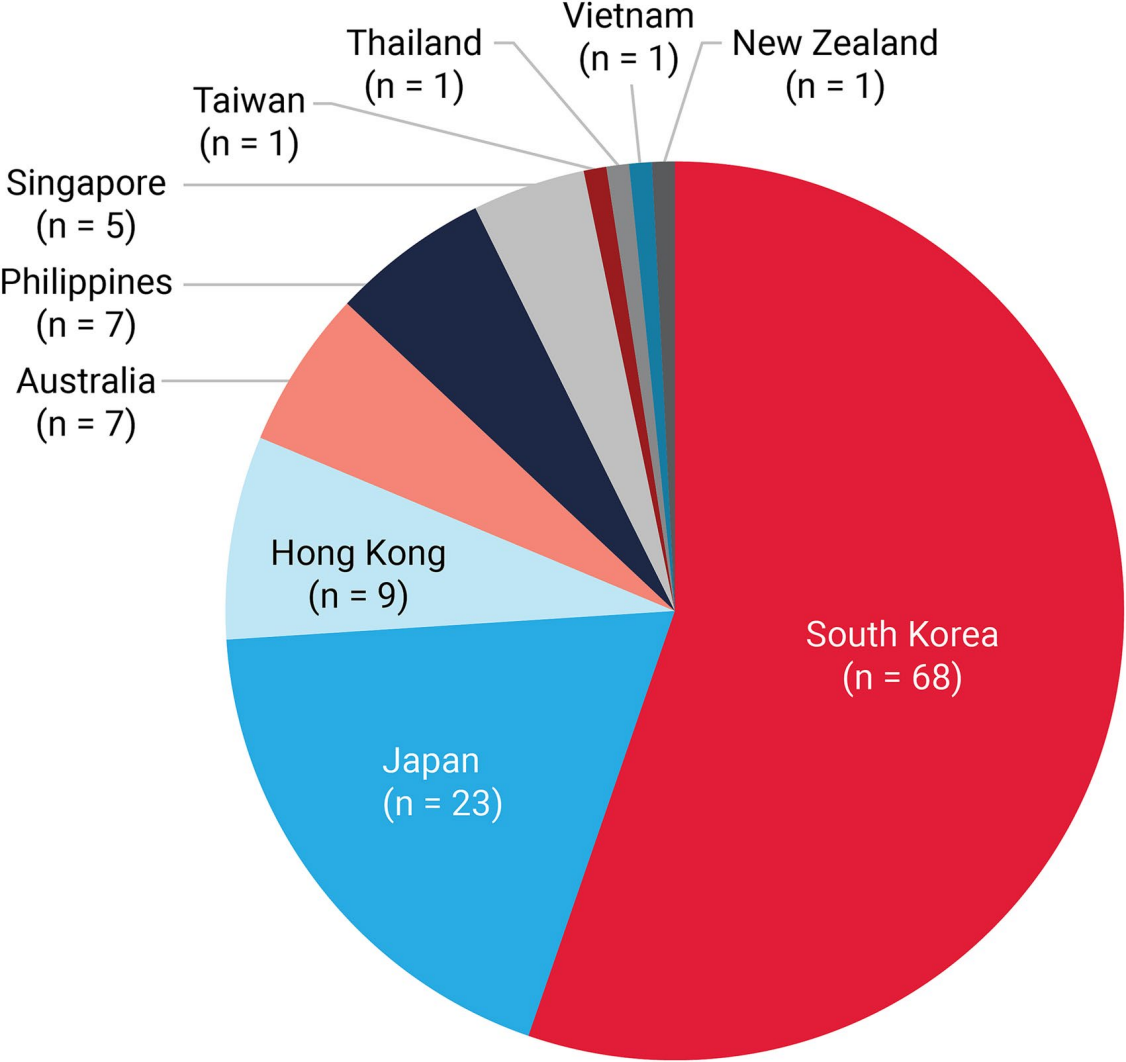
Study search and screening flow diagram. *n*, number of studies.

**Figure 2:**
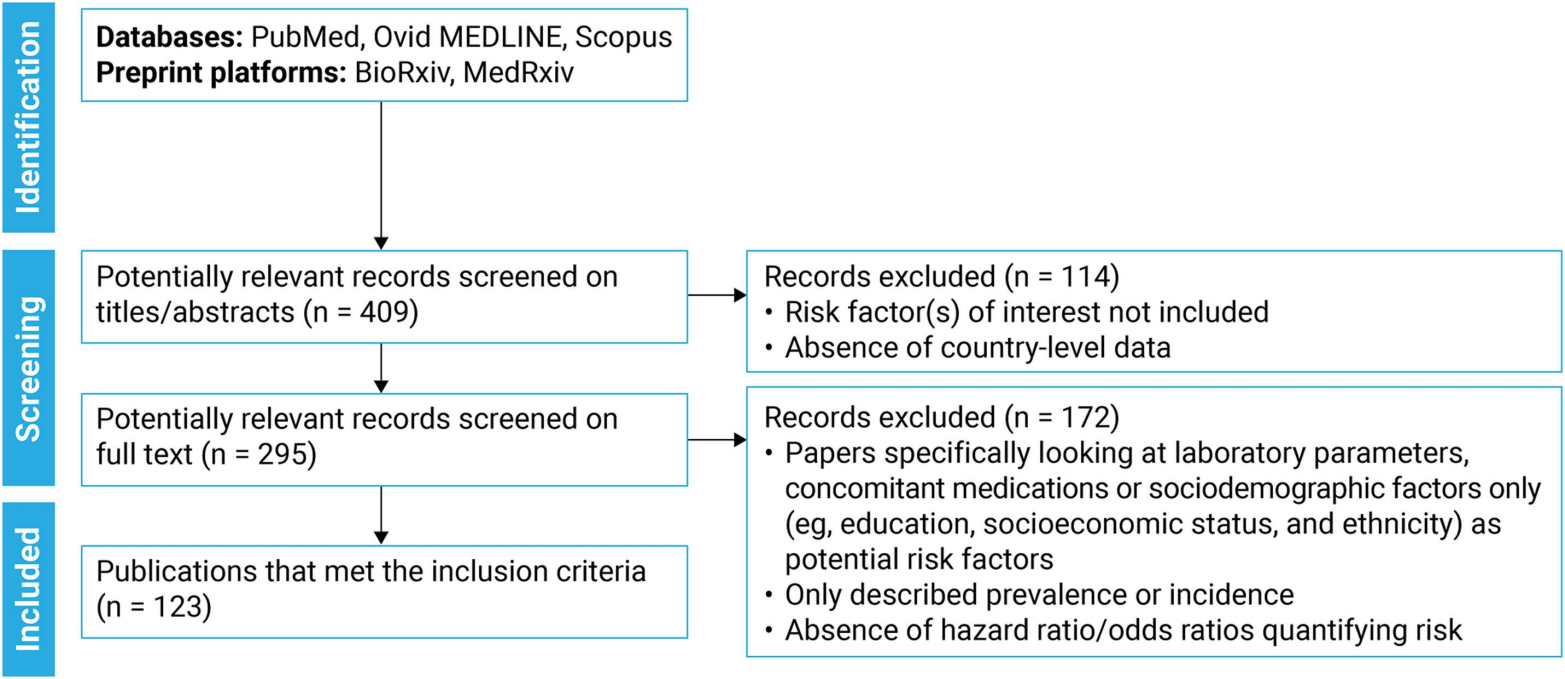
Country/region of origin for included studies^a^ (*N* = 123). *N*, number of studies. ^a^Macau had 0 studies included.

The literature search identified 409 studies; 295 full-text articles were screened, and 123 met the inclusion criteria for data extraction (Figure 1). Most publications originated from South Korea (*n* = 68) and Japan (*n* = 23; Figure 2) and were conducted during early pandemic phases, with 83 studies published in 2020 and 2021. Only 23 studies examined outcomes during periods when the Delta and Omicron SARS-CoV-2 variants were predominant (from mid-2021).

### Key risk factors for severe COVID-19 outcomes and mortality

The number of risk factors included per individual study varied widely (range: 1–24 factors), as did the total study sample sizes (range: 84–937,758 individuals). Studies varied in the definitions and number of severe outcomes tested for statistical association. The risk factors most commonly associated with severe COVID-19 outcomes included older age, male sex, obesity (BMI ≥25 or ≥30 kg/m^2^), diabetes, HF, renal disease (including CKD), and dementia (Table 1). Other associated conditions included hypertension, chronic obstructive pulmonary disease (COPD), CVD, cerebrovascular disease, immunocompromise, autoimmune diseases, and mental illness (Table 1).

**Table 1:** Risk factors for severe COVID-19 and corresponding number, frequency and observed OR/HR ranges for included studies.

Risk factor		Number of studies (*N* = 123)	Studies reporting ≥1 significant outcome, *n* (%)	OR/HR range	Notes on definitions / variability
Age		90	82 (91%)	1.01–377	Higher risk consistently observed with advancing age
Male sex		64	44 (69%)	1.15–15.6*	Most studies defined sex as binary; one found higher risk in women
Obesity (BMI ≥ 25 or ≥30 kg/m^2^)		26	17 (65%)	1.09–26.0	BMI cutoffs varied by country; Asian studies used ≥25
Hypertension		49	20 (41%)	1.13–3.17	Definitions varied and were not always clearly described
Diabetes		70	52 (74%)	1.06–64.1	Included diagnosed, undiagnosed, and prediabetes
Chronic respiratory disease	All	46	18 (39%)	1.04–13.7	Includes COPD and asthma; definitions varied; some studies had small subgroups
	COPD	25	11 (44%)	1.19–8.07	Stronger evidence than for asthma; included in broader respiratory disease category
	Asthma	16	2 (13%)	1.04–4.09	Limited evidence; small subgroups may limit power
Cardiovascular disease	All	45	24 (53%)	1.28–14.3	Included heart failure, IHD, AF; definitions varied across studies
	Heart failure	19	13 (68%)	1.28–4.68	Sometimes grouped under cardiovascular disease
	IHD/CAD	14	4 (29%)	1.52–6.67	Varied definitions; sometimes included in broader cardiovascular disease group
Cerebrovascular disease		24	6 (25%)	1.06–8.58	Case definitions varied (stroke, TIA, CVA); limited statistical power in some studies
Chronic renal disease		38	24 (63%)	1.48–16	Definitions varied (eGFR, ICD codes); small samples common
Hepatobiliary disorders		21	6 (29%)	1.07–5.12	Definitions varied (e.g., NAFLD, cirrhosis); small subgroup sizes
Cancer/malignancy		47	27 (57%)	1.07–13.6	Mostly broad definitions; few studies on specific cancer types
Immunosuppression		11	6 (55%)	1.06–107	Definitions varied; many studies had small sample sizes
Autoimmune disorders		14	4 (29%)	1.19–6.69	Broad disease categories; variable inclusion of rheumatic/connective tissue diseases
Neurologic conditions	All	34	30 (88%)	1.18–11.6	Primarily dementia; other conditions included Parkinson’s and paralysis
	Dementia	26	22 (85%)	1.27–11.6	Often included under ‘neurological conditions’
Mental and behavioral disorders		10	6 (60%)	1.27–3.57	Included severe mental illness, depression and schizophrenia

Based on comparisons using females as the reference group vs males as the comparator group. BMI, body mass index; CAD, coronary artery disease; COPD, chronic obstructive pulmonary disease; IHD, ischemic heart disease.

#### Age

Ninety studies assessed age as a potential risk factor for severe outcomes from COVID-19 infection. Of these, 82 studies (91%) reported a significant association between older age and increased risk of at least one severe COVID-19 outcome (Table 1; Supplementary Table 1). Studies that assessed risk across different age strata consistently reported an incremental increase in risk with advancing age, most notably in adults aged ≥50 years. A nationwide study of 937,758 adults in Japan found that compared with younger adults (aged 20–49 years), the aOR for COVID-19 mortality was 8.8 for those aged 50–64 years (14).

#### Sex

Sixty-four studies compared the risk of severe outcomes from COVID-19 infection between women and men (Table 1). Overall, 44 (69%) found a significantly increased risk for men (Supplementary Table 2); 1 study found an increased risk for women in a South Korean study of 5,571 patients hospitalized with COVID-19 (15), and the remainder did not find any significant differences between sex. Overall, these studies were conducted in a variety of populations (e.g., hospitalized adults) and the potential for underlying sex biases because of sex-associated risk factors for certain medical conditions and differences in health service utilization needs to be considered when interpreting these findings.

#### Obesity (BMI ≥25 or ≥30 kg/m^2^)

Twenty-six studies assessed obesity on the risk of severe COVID-19 outcomes (Table 1). Clinical definitions of obesity varied between studies, with a BMI of ≥25 kg/m^2^ often used for Asian populations compared with the typical threshold of ≥30 kg/m^2^ in non-Asian populations. This variation reflects ethnic differences in body composition and metabolic profiles, highlighting the importance of population-appropriate clinical thresholds when interpreting and comparing COVID-19 risk. Almost two-thirds of the studies (17/26; 65%) showed an increased risk of at least 1 reported outcome in individuals with obesity and/or BMI ≥25 or ≥30 kg/m^2^. No studies showed a decrease in associated risk of severe outcomes among those with obesity. Across studies with large sample sizes (*n* > 1,000) of individuals classified as overweight or obese, the reported OR (95% CI) for severe COVID-19 outcomes ranged from 1.19 (1.02–1.38) to 6.85 (3.18–14.78; Supplementary Table 3). Several studies evaluated risk levels across different BMI categories. Although some studies indicated a higher risk of severe outcomes among individuals with a BMI ≥30 kg/m^2^ vs. those with a BMI ranging from 25 to 29 kg/m^2^, this observation was not uniform across all studies.

#### Hypertension

Forty-nine studies explored hypertension as a potential risk factor (Table 1). The clinical definition of hypertension varied and was not always clearly described. Twenty studies (41%) found a substantially increased risk of severe COVID-19 outcomes among patients with hypertension, including 10 studies with large cohorts (*n* > 1,000). Among these studies, the aORs and aHRs ranged from aHR 1.13 (1.01–1.26) to aOR 3.17 (1.05–10.40; Supplementary Table 4).

#### Diabetes

The impact of diabetes on the risk of severe COVID-19 outcomes was assessed in 70 studies (Table 1). Fifty-two studies (74%) found diabetes was associated with an increased risk of severe outcomes for at least 1 of the outcomes assessed. Of the remainder, 18 studies found no significant association between people with and without diabetes; a single study found a lower risk of COVID-19 hospitalization, albeit in a very small group with diabetes (*n* < 100) in a cross-sectional internet study (16). Among 12 large (*n* > 1,000) studies, the aOR (95% CI) for severe COVID-19 outcomes across the different outcomes reported ranged from 1.06 (1.02–1.10) to 2.02 (1.44–2.84; Supplementary Table 5). Two studies also assessed the risk of severe COVID-19 outcomes among patients with prediabetes and/or undiagnosed diabetes, with contrasting results (17, 18).

#### Chronic respiratory diseases

A total of 46 studies investigated chronic respiratory diseases, including COPD (25 studies) and asthma (16 studies; Table 1). Eighteen studies (39%) reported an increased risk of severe outcomes among patients with a chronic respiratory disease for at least 1 reported outcome. Among the different case definitions for chronic respiratory disease, more evidence was found for COPD (11/25 studies) than asthma (2/16 studies). Many studies included small subpopulations, potentially limiting their ability to accurately detect risk. For larger studies (risk factor cohort *n* > 50), the reported OR (95% CI) ranged from aOR, 1.04 (1.01–1.08) to OR, 4.19 (1.60–10.98; Supplementary Table 6).

#### Cardiovascular diseases

Forty-five studies assessed CVD, including HF (19 studies) and ischemic heart disease/coronary artery disease (14 studies), using varying case definitions. Overall, 24 (53%) studies found a substantially increased risk of severe COVID-19 outcomes from at least 1 of the outcomes assessed (Table 1). Among those exploring HF, 13/19 studies (68%) found a significant association with severe COVID-19 outcomes with the aOR (95% CI) ranging from 1.28 (1.24–1.33) to 3.17 (1.88–5.34; Supplementary Table 7). Two of the 5 studies investigating atrial fibrillation (AF) and 1 of the 7 studies investigating peripheral vascular disease (PVD) reported a significant risk of severe COVID-19 outcomes (19, 20).

#### Cerebrovascular diseases

Twenty-four studies assessed the risk of severe COVID-19 outcomes in patients with cerebrovascular diseases (Table 1), including 15 studies examining cerebrovascular disease broadly and 7 studies specifically addressing conditions defined as stroke, transient ischemic attack (TIA), or cerebrovascular accident (CVA), with varying case definitions. Five of the 15 studies of cerebrovascular disease and 1 of the 7 studies of patients with a history of stroke/TIA/CVA showed an increased risk of severe COVID-19 outcomes (Supplementary Table 8).

#### Renal disease

Thirty-eight studies assessed renal disease as a risk factor for severe COVID-19 outcomes, including CKD (24 studies), renal/kidney disease (10 studies), and renal failure (2 studies). Case definitions and criteria used to define CKD varied and cohort sizes were generally small (*n* < 300). Of these, 24/38 studies (63%) showed a significantly increased risk of severe COVID-19 outcomes in patients with preexisting kidney/renal disease (Table 1). For studies with a risk factor cohort >50 patients, the aOR (95% CI) for severe outcomes ranged from 1.48 (1.42–1.55) to 5.59 (2.48–12.63; Supplementary Table 9). In a large study from Japan that included 20,638 adults with renal disease using broad International Classification of Diseases, Tenth Revision (ICD-10) codes, the aORs (95% CI) were 1.48 (1.42–1.55) for severe COVID-19 disease and 1.67 (1.59–1.75) for mortality (14).

#### Hepatobiliary disorders (including chronic liver disease)

Twenty-one studies assessed whether hepatobiliary disorders were associated with severe outcomes after COVID-19 infection. Case definitions varied, ranging from broad categories like chronic liver disease and liver disease (9 studies) to specific conditions like chronic hepatitis or cirrhosis, hepatitis B/C, and non-alcoholic fatty liver disease (NAFLD). Overall, 6 studies (29%) showed that hepatobiliary disorders were associated with an increased risk of severe outcomes (Table 1). Of these, 5 studies assessed liver disease/cirrhosis and the sixth study of a large, nationwide study in South Korea found that NAFLD was associated with an increased risk of severe COVID-19 outcomes, although results for mortality were less certain and showed wide confidence intervals (Supplementary Table 10) (21).

#### Cancer/malignancy

Forty-seven studies explored cancer as a potential risk factor with almost all studies using broad case definitions such as ‘cancer’, ‘malignancy’ or ‘malignant tumor’. Five studies focused on specific cancer types. Cohort sizes also varied widely, ranging from 6 to 42,854 participants. Overall, 27/47 studies (57%) found a significantly increased risk of severe COVID-19 outcomes (Table 1). In a large Korean study, malignancy was associated with an increased risk of severe COVID-19 outcomes including invasive mechanical ventilation (IMV) and other markers of disease severity (high-flow nasal cannula use, acute HF and renal replacement therapy; Supplementary Table 11) (22). However, malignancy was not found to be an independent risk factor for in-hospital mortality among patients with COVID-19 (aOR, 1.24 [95% CI, 0.92–1.66]) (22). Variations between countries in the associations between cancer and COVID-19 related hospitalization and mortality should be interpreted in the context of regional palliative care service provision and cultural variations in place of death.

#### Immunosuppression

Eleven studies evaluated the risk of severe COVID-19 outcomes in populations with immune-suppressive illnesses or those receiving immunosuppressive medications (Table 1). Five studies focused on HIV/AIDS and 6 studies examined individuals described as immunocompromised, immunosuppressed, or having immunodeficiency, although definitions between studies varied (e.g., use of corticosteroids, biologics, and immunosuppressive drugs; lymphocytopenia; bone marrow dysfunction; hospitalization with organ transplant, hematopoietic cancer, rheumatoid arthritis, Crohn’s disease, or ulcerative colitis). Ten studies included very small cohorts of patients with immunosuppression, ranging from 4 to 129 participants, along with 1 larger study from Korea with 5,186 participants (22). Therefore, quantitative approaches to assess associations and the ability to adjust for confounding factors were limited. Overall, 6/11 studies (55%) found a significantly increased risk of severe COVID-19 outcomes in patients who were immunosuppressed/ immunocompromised. The aOR (95% CI) in these studies varied from 1.06 (1.05–1.07) for IMV to 107 (6.38 to >999) for mortality (Supplementary Table 12).

#### Autoimmune disorders (rheumatic and connective tissue diseases)

Fourteen studies investigated whether autoimmune disorders increased the risk of severe outcomes from COVID-19 infection. Most studies used broad case definitions such as ‘rheumatic disease’, ‘autoimmune disease’, and ‘connective tissue disease.’ Overall, 4 of the 14 studies (29%) showed a significant increased risk in patients with autoimmune or connective tissue disorders for at least 1 outcome (Table 1). Among the 3 studies including >1,000 participants with autoimmune diseases, the aOR (95% CI) for severe COVID-19 outcomes and/or mortality ranged from 1.19 (1.12–1.26) to 1.81 (1.02–3.18; Supplementary Table 13). In a Korean nationwide cohort study of >8,000 patients with autoimmune inflammatory rheumatic diseases (inflammatory arthritis or connective tissue disease), high-dose corticosteroids use (≥10 mg/day), but not disease-modifying antirheumatic drugs, was associated with increased risk of severe COVID-19 infection and COVID-19–related death (23).

#### Neurological conditions

Thirty-four studies explored neurological conditions as potential risk factors, with most focusing on dementia (26 studies), hemiplegia/paraplegia/paralysis (4 studies), chronic neurological disorders (3 studies), and Parkinson disease (2 studies). Overall, 30/34 studies (88%) found a significantly increased risk of severe COVID-19 outcomes in patients with neurological conditions (Table 1). A large Japanese study that included >20,000 patients with COVID-19 with diagnosed dementia (using ICD-10 codes) found it was an independent risk factor for severe COVID-19 outcomes (aOR, 1.27 [95% CI, 1.23–1.32]) and death (1.58 [1.52–1.65]; Supplementary Table 14) (14). Another Japanese study observed a greater than 3-fold increased risk of mortality among adults aged >50 years with Parkinson’s disease (OR: 3.57 [95% CI: 1.08, 10.2]) (24).

#### Mental health and behavioral disorders

Ten studies investigated mental and behavioral disorders and the risk of severe outcomes from COVID-19 infection. Definitions varied across studies and included ‘mental disorder’ and ‘mental illnesses,’ for which studies were larger, and more specific conditions, including schizophrenia and depression/anxiety, wherein cohort sizes were smaller. Overall, 6/10 studies (60%) found a significantly increased risk of severe COVID-19 outcomes for patients with mental and behavioral disorders (Table 1). The aOR (95% CI) for severe COVID-19 outcomes ranged from 1.27 (1.01–1.66) to 3.57 (1.36–9.38; Supplementary Table 15). In a Korean nationwide cohort study of >1,000 patients with a previous diagnosis of a mental illness, severe mental illness (nonaffective or affective disorders with psychotic features) was associated with an increased risk of severe COVID-19 outcomes (OR: 1.27 [95% CI: 1.01, 1.66]) (25).

#### Other medical conditions

There were 18 studies that were categorized as other medical conditions, of which 9 showed significant associations (Supplementary Table 16).

## Discussion

This review identified several risk factors associated with severe COVID-19 outcomes, including older age, male sex, obesity (BMI ≥25 or ≥30 kg/m^2^), HF, chronic renal disease (including CKD), cancer, immunosuppression and dementia, consistent with global reviews and meta-analyses (2, 26–28). Some risk factors, such as older age and immunocompromise align with current COVID-19 recommendations in the APAC region. However, a large volume of evidence was associated with other conditions, including CVD, hypertension, diabetes (and prediabetes), kidney disease and liver disease, less consistently reflected in regional vaccine guidance. Regional authorities and medical society guidance varies, and often broad disease categories for COVID-19 vaccination are advised, with limited examples of specific conditions. This may result in vulnerable populations being overlooked for targeted prevention.

Associations with severe COVID-19 outcomes were identified under the categories of hypertension, diabetes (including prediabetes), and CVD. This finding is consistent with a recent meta-analysis of cardiometabolic risk factors in the APAC region that demonstrated a 2.85-fold increase in COVID-19–associated mortality for diabetes, a 2.5-fold increase for hypertension, and a 2.75-fold increase for CVD (29). A large volume of evidence was identified to indicate an association between HF and severe COVID-19 outcomes. Given the rapidly rising prevalence of HF in the region, and the earlier onset observed in Asian populations compared to other regions (11), the findings highlight the need for increased awareness of preventative strategies among younger individuals with HF. By 2050, ischemic heart disease and stroke are projected to be the most prevalent subtypes of CVD in the region, with high systolic blood pressure the leading risk factor (8). Regional variations in CVD burden indicate high ischemic heart disease mortality in Central Asia, while stroke is a key driver in Southeast Asia (30). This is largely attributed to regional differences in the burden of AF and flutter, leading to a rise in thromboembolic strokes. Ongoing focus on effective strategies to reduce the burden of CVD-associated severe COVID-19 outcomes among Asian populations is warranted from this review.

This review identified an association between severe COVID-19 disease and neurological conditions, particularly dementia. This finding is consistent with global studies reporting a > 7-fold increased risk of COVID-19 hospitalization and death among older patients with preexisting dementia (31), a phenomenon that has biological (neuronal injury) and environmental explanations (32, 33). Importantly, with Asia now at the forefront of population aging, and Hong Kong, South Korea and Japan expected to have approximately 40% of their populations aged ≥65 years by 2050 (34), regional dementia case estimates are rising in the APAC region (35). Mental and behavioral disorders were found to be associated with an increased risk of severe COVID-19 outcomes. These conditions, along with dementia, are notably under-represented in regional guidelines. This review supports global evidence, for example, a UK Biobank study showing individuals with serious mental illness (SMI) were hospitalized with COVID-19 twice as often as those without SMI (36). SMI was also associated with higher rates of illness and death than heart disease, COPD, and diabetes.

This review suggests that the risk of severe COVID-19 outcomes in patients with liver and kidney disease may be influenced by the stage or severity of these conditions. Global studies have shown that patients with advanced liver disease, such as cirrhosis, and those with CKD stages 4–5 are at substantially higher risk of severe COVID-19 complications which is reflected in current vaccine recommendations across the region (37, 38). While current vaccine recommendations typically focus on the presence of individual medical conditions, it is crucial to consider the cumulative burden of conditions when assessing the risk of severe COVID-19 disease. Given the high prevalence and clustering of cardiometabolic conditions in APAC populations, such as hypertension, diabetes, and increasingly, obesity, risk assessment tools and vaccine prioritization strategies may be enhanced by incorporating measures of multimorbidity (11). Specific recommendations for COVID-19 vaccination for those with multimorbidity and elevated metabolic risks may be warranted. Other regionally relevant exposures, such as elevated smoking prevalence in certain APAC subgroups, may also contribute to underlying cardiovascular and respiratory vulnerability, and warrant further exploration.

A key strength of this analysis is its broad and inclusive review of the available literature across selected APAC countries; however, several limitations must be acknowledged. As a narrative review focused on conceptual themes in the literature, no formal risk-of-bias assessment was conducted, nor was publication bias quantified, as would typically be done in a systematic review. This synthesis is based on published studies and may inadvertently over-represent positive findings due to publication bias. A high degree of heterogeneity was observed across studies, including variations in the definitions of risk conditions and severe outcomes (influenced by definitions and data availability in national health and insurance databases), reported parameters, statistical analyses, modeling approaches, and adjustment for confounding factors. Some studies had small samples sizes, limiting the precision of estimates and the ability to adjust for covariates. Most studies did not account for smoking or medication use for comorbidities, and residual confounding and statistical robustness were limitations of some included studies. Most included studies collected data early in the pandemic, before the introduction of vaccines and/or specific antiviral medications, potentially limiting the applicability of findings to the current context in which these interventions are widely available. Future research should aim to standardize definitions of risk factors and outcomes, adopt more robust confounding adjustments, and include studies that evaluate post-vaccination era cohorts. Regional multicenter collaborations could help reduce heterogeneity and support targeted systematic reviews focusing on specific risk factors identified in this review. Where appropriate, meta-analyses of these data could more accurately quantify risk among Asian populations and improve generalizability.

In summary, this review synthesizes region-specific evidence, identifying several under-recognized risk factors for severe COVID-19 outcomes. These findings complement global evidence but also highlight important regional nuances. Improved identification of high-risk groups, particularly those with dementia, mental illness, and cardiometabolic multimorbidity, can strengthen targeted prevention efforts in APAC settings.

## Conclusion

This review appears to be the first synthesis of risk factors for severe COVID-19 outcomes specific to the Asia-Pacific region, based on currently available evidence. Beyond the well-established risks of older age and immunocompromise, several high-priority conditions, such as AF, PVD, dementia, and mental health disorders, were identified as often overlooked in current guidance. To improve protection for vulnerable groups, public health agencies in APAC should update vaccination frameworks to explicitly include these conditions, expand access to boosters and antivirals for individuals with multimorbidity, and integrate region-specific evidence into national risk assessment and public health messaging. Continued surveillance and evidence synthesis will be critical to adapting COVID-19 policy for emerging variants and population needs.
