# Supplementary material for: Risk factors for severe COVID-19 outcomes in the Asia-Pacific region: a literature review
Source: Front Public Health. 2025 Jun 9;13:1562179. doi: 10.3389/fpubh.2025.1562179 (PMC12224185; doi:10.3389/fpubh.2025.1562179)
Supplement: Supplementary file 1 [file Supplementary_file_1.docx]

# Supplementary Appendix

# Supplementary Methods

*Search strategy and eligibility criteria*

Key search terms included ‘COVID-19’, ‘risk’, and ‘severe’; the countries and potential risk factors of interest were identified following a review of global and local guidelines. The search terms and key inclusion/exclusion criteria are presented in **Supplementary Tables 17 and 18**. We reviewed National Immunization Technical Advisory Group (NITAG) and Advisory Committee on Immunization Practices (ACIP) recommendations for each of the countries/regions of interest to understand current recommendations related to risk factors and medical conditions to ensure inclusion in this review. A summary table of APAC NITAG and ACIP guidance, including medical conditions and descriptions of high-risk groups, is presented in **Supplementary Tables 19 and 20**.

The primary endpoints of interest were severe COVID-19 outcomes, defined as hospitalization, ICU admission, and COVID-19 mortality. Advanced respiratory support (mechanical ventilation, extracorporeal membrane oxygenation [ECMO]), and oxygen therapy) and acute respiratory failure were also included as surrogate markers of severe COVID-19. Definitions for severe/critical COVID-19 outcomes differed across studies (**Supplementary Table 21**).

This review focused on medical conditions associated with severe COVID-19 outcomes. We therefore excluded studies that only described sociodemographic associations, those that only investigated laboratory parameters and medication associations with COVID-19 severe outcomes without detailing a medical condition, and studies that only reported the incidence or prevalence of a disease without using quantitative estimates (ie, odds ratios or hazards ratios) of risk factors for severe COVID-19 outcomes.

*Screening and data synthesis*

A two-stage study selection process was conducted. First, titles and abstracts were reviewed, followed by full-text articles, which was performed independently by 2 researchers (M.T, and C.C.) to determine eligibility using the inclusion and exclusion criteria, as documented in the flow diagram (**Figure 1**). For preprint publications, a forward search was conducted and data from the final publication, if available, were used for data extraction. Any disagreements during the selection process were resolved through discussion between the 2 reviewers, with a third reviewer (A.K.B.) available to resolve any issues if they arose. Data were extracted from full-text articles, including geographic location of the study, study design, study population, sample size/case numbers, risk factors, comparator groups, and results for all outcomes of interest (reported adjusted odds ratios, hazard ratios, 95% confidence intervals, and p-values where available). Results were then grouped by individual risk factors (eg, age, medical condition) for all outcomes (eg, hospitalization, mortality). Both positively and negatively associated results were tabulated and interpreted through narrative analysis.

Included risk factors were age, sex, obesity, hypertension, diabetes, CVD, cerebrovascular disease, chronic respiratory disease, renal disease (including CKD), hepatobiliary diseases (including chronic liver disease and non-alcoholic fatty liver disease [NAFLD]), cancer and malignancy, immunosuppression, autoimmune disorders (including rheumatic and connective tissue diseases), neurological conditions, mental and behavioural disorders, and ‘other’ medical conditions (with ‘other’ defined as medical conditions included in fewer than 5 studies in the review).

### Supplementary Table 1. Studies assessing age

| **First author** | **Year** | **Country** | **Total** | **Sample** | **Reference group**  **(sample size)** | **Risk factor**  **(sample size)** | **Outcome** | **OR/HR (95% CI)** | ***P*-value** |
| --- | --- | --- | --- | --- | --- | --- | --- | --- | --- |
| Bhatia[1] | 2021 | Australia | 546 | 546 | Younger age (n = NR) | Older age (n = NR) | Mortality | **1.09 (1.07, 1.12)** | <.001 |
| Davis[2] | 2022 | Australia | 794^a^ | 794 | 16–40 yrs (n = 494) | 40–65 yrs (n = 189) | Hospitalization | **1.92 (1.12, 2.30)** | .02 |
|  |  |  |  |  |  | >65 yrs (n = 31) |  | **7.05 (2.64, 18.8)** | <.001 |
| Ellis[3] | 2023 | Australia | 1071^b^ | 1,071 | <70 yrs (n = 84) | Per 5 years (n = NR) | Hospitalization | 1.06 (0.96, 1.17) | NR |
|  |  |  |  |  |  | 70–79 yrs (n = 250) |  | 1.0 (0.5, 2.0) | NR |
|  |  |  |  |  |  | 80–89 yrs (n = 464) |  | 1.4 (0.7, 2.7) | NR |
|  |  |  |  |  |  | ≥90 yrs (n = 273) |  | 1.1 (0.5, 2.3) | NR |
|  |  |  |  |  |  | Per 5 years (n = NR) | Mortality | **1.38 (1.21, 1.57)** | NR |
|  |  |  |  |  |  | 70–79 yrs (n = 250) |  | 1.2 (0.4, 3.4) | NR |
|  |  |  |  |  |  | 80–89 yrs (n = 464) |  | **2.9 (1.1, 7.5)** | NR |
|  |  |  |  |  |  | ≥90 yrs (n = 273) |  | **3.5 (1.3, 9.2)** | NR |
| Liu[4] | 2021 | Australia | 4054 | 4,054 | 30–39 yrs (n = 721) | 20–29 yrs (n = 844) | Severe COVID-19^c^ | 0.43 (0.26, 0.72) | NR |
|  |  |  |  |  |  | 40–49 yrs (n = 507) |  | 1.19 (0.77, 1.82) | NR |
|  |  |  |  |  |  | 50–59 yrs (n = 582) |  | **1.68 (1.15, 2.46)** | NR |
|  |  |  |  |  |  | 60–69 yrs (n = 558) |  | **2.66 (1.86, 3.81)** | NR |
|  |  |  |  |  |  | 70–79 yrs (n = 356) |  | **3.53 (2.44, 5.11)** | NR |
|  |  |  |  |  |  | 80–89 yrs (n = 118) |  | **8.60 (5.76, 12.9)** | NR |
|  |  |  |  |  |  | ≥90 yrs (n = 41) |  | **10.9 (6.46, 18.3)** | NR |
|  |  |  |  |  |  | 20–29 yrs (n = 844) | Very severe COVID-19^d^ | 0.27 (0.10, 0.74) | NR |
|  |  |  |  |  |  | 40–49 yrs (n = 507) |  | 0.80 (0.37, 1.76) | NR |
|  |  |  |  |  |  | 50–59 yrs (n = 582) |  | 1.84 (0.99, 3.42) | NR |
|  |  |  |  |  |  | 60–69 yrs (n = 558) |  | **3.13 (1.75, 5.59)** | NR |
|  |  |  |  |  |  | 70–79 yrs (n = 356) |  | **4.45 (2.49, 7.97)** | NR |
|  |  |  |  |  |  | 80–89 yrs (n = 118) |  | **8.43 (4.44, 16.0)** | NR |
|  |  |  |  |  |  | ≥90 yrs (n = 41) |  | **16.2 (7.77, 33.8)** | NR |
| Muleme[5] | 2023 | Australia | 2876^b^ | 2,876 | Younger age (n = NR) | Older age (n = NR) | Hospitalization | 1.00 (0.99, 1.01) | .98 |
|  |  |  |  |  |  |  | Mortality | **1.05 (1.04, 1.07)** | <.001 |
| Shiel[6] | 2021 | Australia | 517 | 517 | ≤80 yrs (n = NR) | >80 yrs (n = NR) | Mortality | **24.4 (4.1, 145)** | <.001 |
|  |  |  |  |  |  |  | Severe COVID-19^d^ | NS | NS |
|  |  |  |  |  |  |  | Incomplete recovery at 30 days | **5.6 (1.9, 16.7)** | .002 |
| Wang[7] | 2023 | Australia | 1082 | 1082 | <40 yrs (n = NR) | 40–59 yrs (n = NR) | Oxygen requirement | **8.58 (2.84, 25.9)** | <.001 |
|  |  |  |  |  |  | ≥60 yrs (n = NR) |  | **10.2 (3.85, 27.2)** | <.001 |
| Chung[8] | 2021 | Hong Kong | 3074 | 3074 | Younger age (n = NR) | Older age (n = NR) | Severe COVID-19 | **1.08 (1.07, 1.09)** | <.001 |
| Fan [9] | 2021 | Hong Kong | 4834 | 3164 | >60 yrs (n = NR) | 60–79 yrs (n = NR) | Mortality | **14.8 (3.06, 71.2)** | <.001 |
|  |  |  |  |  |  | 70–79 yrs (n = NR) |  | **54.6 (12.5, 239)** | <.001 |
|  |  |  |  |  |  | 80 yrs (n = NR) |  | **163 (38.3, 691)** | <.001 |
| Teoh [10] | 2020 | Hong Kong | 2710 | 814 | Younger age (n = NR) | Older age (n = NR) | Severe COVID-19 | 1.02 (1.00,1.04) | .05 |
| Wong[11] | 2023 | Hong Kong | 32 222 | 22 604 | 45–64 yrs (n = 2574) | 18–44 yrs (n = 683) | Mortality | 0.45 (0.33, 0.59) | NR |
|  |  |  |  |  |  | 65–79 yrs (n = 6091) |  | **1.50 (1.34, 1.68)** | NR |
|  |  |  |  |  |  | ≥80 yrs (n = 12 748) |  | **2.88 (2.59, 3.21)** | NR |
| Yip[12] | 2021 | Hong Kong | 5639 | 5639 | Younger age (n = NR) | Older age (n = NR) | Mortality | **1.09 (1.07, 1.11)** | <.001 |
| Zhou[13] | 2023 | Hong Kong | 6089 | 6089 | Younger age (n = NR) | Older age (n = NR) | Severe COVID-19 | **1.07 (1.06, 1.08)** | <.0001 |
| Zhou[14] | 2020 | Hong Kong | 1043 | 1043 | Younger age (n = NR) | Older age (n = NR) | ICU admission | **1.06 (1.03, 1.09)** | <.0001 |
|  |  |  |  |  | NR (n = NR) | 18–24 yrs (n = 1) |  | 0.23 (0.03, 1.74) | .1542 |
|  |  |  |  |  |  | 25–49 yrs (n = 5) |  | 0.48 (0.17, 1.35) | .1662 |
|  |  |  |  |  |  | 50–64 yrs (n = 6) |  | 1.80 (0.68, 4.79) | .2395 |
|  |  |  |  |  |  | 65–74 yrs (n = 6) |  | **8.29 (3.03, 22.7)** | <.0001 |
|  |  |  |  |  |  | ≥75 yrs (n = 1) |  | 2.22 (0.29, 17.3) | .4463 |
| Zhou[15] | 2020 | Hong Kong | 2774 | 2774 | Younger age (n = NR) | Older age (n = NR) | Mortality | **1.13 (1.09, 1.2)** | <.0001 |
| Fukushima [16] | 2021 | Japan | 234 | 234 | <45 yrs (n = NR) | ≥45 yrs (n = NR) | Critical events | **12.3 (2.98, 67.7)** | NR |
| Fukushima [17] | 2023 | Japan | 2430 | 2430 | <65 yrs (n = NR) | ≥65 yrs (n = NR) | Critical events | **1.78 (1.28, 2.47)** | .0005 |
| Ito[18] | 2023 | Japan | 936 | 936 | ≤29 yrs (n = 512) | 30–39 yrs (n = 139) | Hospitalization | 2.35 (0.76, 7.30) | .139 |
|  |  |  |  |  |  | 40–49 yrs (n = 160) |  | **7.00 (2.94,16.7)** | <.001 |
|  |  |  |  |  |  | ≥50 yrs (n = 125) |  | **14.2 (6.18, 32.6)** | <.001 |
| Matsushita [19] | 2022 | Japan | 23 414 | 23 414 | Younger age (n = NR) | Older age per 10 yrs increase (n = NR) | COVID severity grade 2/3/4/5^e^ | **1.90 (1.81, 2.00)** | NR |
|  |  |  |  |  |  |  | COVID severity grade 3/4/5^e^ | **2.21 (2.07, 2.35)** | NR |
|  |  |  |  |  |  |  | Mortality | **3.16 (2.88, 3.47)** | NR |
| Miyashita [20] | 2022 | Japan | 937 758 | 937 758 | 20–49 yrs (n = 577 256) | 50–64 yrs (n = 185 088) | Severe COVID-19 or death | **6.04 (5.78, 6.32)** | <.001 |
|  |  |  |  |  |  | 65–79 yrs (n = 104 561) |  | **15.0 (14.4, 15.7)** | <.001 |
|  |  |  |  |  |  | 80+ yrs (n = 70 853) |  | **29.2 (27.8, 30.6)** | <.001 |
|  |  |  |  |  |  | 50–64 yrs (n = 185 088) | Mortality | **8.82 (7.76, 10.0)** | <.001 |
|  |  |  |  |  |  | 65–79 yrs (n = 104 561) |  | **56.7 (50.4, 63.8)** | <.001 |
|  |  |  |  |  |  | 80+ yrs (n = 70 853) |  | **190 (169, 213)** | <.001 |
| Muto[21] | 2021 | Japan | 300 | 300 | <65 yrs (n = 182) | ≥65 yrs (n = 118) | Critical/severe COVID-19 | **2.81 (1.43, 5.56)** | .003 |
| Ninomiya[22] | 2021 | Japan | 210 | 210 | Younger age (n = NR) | Older age per 10 yrs increase (n = NR) | Worsened COVID-19/ oxygen administration | **1.54 (1.22, 1.95)** | <.001 |
| Nishida[23] | 2022 | Japan | 393 | 393 | Younger age (n = NR) | Older age (n = NR) | Mortality^f^ | **1.16 (1.08, 1.26)** | <.001 |
|  |  |  |  |  |  |  | Mortality^g^ | **1.12 (1.04, 1.21)** | .023 |
|  |  |  |  |  |  |  | IMV^f^ | **1.04 (1.01, 1.07)** | <.001 |
|  |  |  |  |  |  |  | IMV^g^ | 1.03 (1.00, 1.07) | .002 |
|  |  |  |  |  |  |  | Mortality or IMV^f,h^ | **1.06 (1.02, 1.09)** | .04 |
|  |  |  |  |  |  |  | Mortality or IMV^f,h^ | **1.05 (1.01, 1.08)** | .004 |
| Nishimura [24] | 2023 | Japan | 30 130 | 1097 | ≤34 yrs (n = 292) | 35–49 yrs (n = 128) | Hospitalization | 1.17 (0.83, 1.66) | .371 |
|  |  |  |  |  |  | 50–64 yrs (n = 41) |  | 1.63 (0.97, 2.73) | .065 |
|  |  |  |  |  |  | ≥65 yrs (n = 21) |  | 1.23 (0.64, 2.37) | .54 |
|  |  |  |  |  |  | 35–49 yrs (n = 117) | Oxygen administration | 0.90 (0.63, 1.30) | .583 |
|  |  |  |  |  |  | 50–64 yrs (n = 34) |  | 1.12 (0.63, 1.98) | .704 |
|  |  |  |  |  |  | ≥65 yrs (n = 65) |  | 1.33 (0.66, 2.67) | .419 |
| Numaguchi [25] | 2022 | Japan | 415 | 415 | Younger age (n = NR) | Older age per 10 yrs increase (n = NR) | Severe COVID-19 | **1.56 (1.27, 1.91)** | <.001 |
| Okauchi[26] | 2021 | Japan | 84 | 84 | <65 yrs (n = 58) | ≥65yrs (n = 26) | Oxygen supplementation | **8.8 (1.62, 47.8)** | .012 |
| Otoshi[27] | 2021 | Japan | 300 | 300 | Younger age (n = NR) | Older age (n = NR) | Severe COVID-19 | **1.05 (1.02, 1.08)** | .003 |
| Sakamoto [28] | 2022 | Japan | 6176 | 6176 | ≤39 yrs (n = 3966) | 40–49 yrs (n = 1083) | Hospitalization | **2.24 (1.57, 3.19)** | NR |
|  |  |  |  |  |  | 50–59 yrs (n = 897) |  | **5.07 (3.67, 7.00)** | NR |
|  |  |  |  |  |  | ≥60 yrs (n = 230) |  | **10.2 (6.72, 15.6)** | NR |
| Sato[29] | 2022 | Japan | 500 | 500 | <60 yrs (n = NR) | ≥65 yrs (n = NR) | Mortality or ECMO or IMV or MV or ICU^h^ | **2.84 (1.33, 6.05)** | .007 |
| Takeyama [30] | 2022 | Japan | 2894 | 2894 | <70 yrs (n = | >70 yrs (n = 535) | Mortality | **7.96 (5.05, 12.6)** | <.001 |
| Tanaka[31] | 2021 | Japan | 3192^i^ | 3192 | 60–69 yrs (n = 1140) | 70–79 yrs (n = 1058) | Mortality | **2.62 (1.63, 4.23)** | NR |
|  |  |  |  |  |  | 80–89 yrs (n = 749) |  | **5.99 (3.77, 9.50)** | NR |
|  |  |  |  |  |  | ≥90 yrs (n = 245) |  | **10.2 (6.03, 17.4)** | NR |
| Yamada[32] | 2021 | Japan | 6873 | 1891 | 18–29 yrs (n = 1891) | ≥30 yrs (n = NR) | Oxygen therapy | **3.46 (1.90, 6.28)** | <.001 |
|  |  |  |  |  | 40–49 yrs (n = NR) | 50–59 yrs (n = NR) |  | **1.61 (1.20, 2.16)** | .002 |
|  |  |  |  |  |  | 60–64 yrs (n = NR) |  | **3.62 (2.49, 5.27)** | <.001 |
|  |  |  |  |  | ≥65 yrs (n = 1007) | ≥75yrs (n = NR) |  | **1.98 (1.47, 2.67)** | <.001 |
| Jefferies[33] | 2020 | New Zealand | 1503 | 1495 | 20–34 yrs (n = 506) | 35–49yrs (n = 298) | Severe COVID-19 | 1.2 (0.55, 2.63) | NR |
|  |  |  |  |  |  | 50–64yrs (n = 340) |  | **2.72 (1.40, 5.30)** | NR |
|  |  |  |  |  |  | 65–79yrs (n = 156) |  | **5.42 (2.54, 11.6)** | NR |
|  |  |  |  |  |  | ≥80 yrs (n = 39) |  | **8.25 (2.59, 26.3)** | NR |
| Anlacan[34] | 2023 | Philippines | 10 881 | 10 881 | 19–59yrs (n = 7047) | ≥60 yrs (n = 3834) | Mortality | **2.33 (1.11, 2.57)** | <.001 |
|  |  |  |  |  |  |  | Respiratory failure | **2.73 (2.47, 3.01)** | <.001 |
|  |  |  |  |  |  |  | ICU admission | **2.56 (2.33, 2.82)** | <.001 |
| Espiritu[35] | 2021 | Philippine | 10 881 | 10881 | Lower age (n = NR) | Higher age (n = NR) | Mortality | **1.01 (1.01, 1.01)** | <.001 |
|  |  |  |  |  |  |  | Respiratory failure | 1 (1, 1) | .536 |
|  |  |  |  |  |  |  | ICU admission | 1 (1, 1) | .63 |
| Koh[36] | 2021 | Singapore | 1042 | 1042 | Younger age (n = NR) | Older age (n = NR) | Severe COVID-19 | **1.04 (1.01, 1.07)** | .004 |
| Lim[37] | 2021 | Singapore | 249 | 249 | Younger age (n = NR)* *Clinical Frailty Scale | Older age (n = NR)* *Clinical Frailty Scale | Severe COVID-19 | 1.06 (0.99, 1.12) | .079 |
|  |  |  |  |  |  |  |  | 1.05 (0.97, 1.13) | .211 |
| Ngiam[38] | 2022 | Singapore | 553 | 553 | Older age (n = NR) | Young age (n = NR) | Severe COVID-19 | **1.06 (1.01, 1.11)** | .019 |
| Tan[39] | 2022 | Singapore | 294 | 294 | Younger age (n = NR) | Older age (n = NR) | ICU admission and/or mortality | **1.13 (1.03, 1.27)** | .02 |
|  |  |  |  |  |  |  | Pneumonia | 1.07 (1.00, 1.16) | .049 |
| Tee[40] | 2020 | Singapore | 240 | 201 | <50 yrs (n = 179)* | ≥50 yrs (n = 61) | Pneumonia | **3.13 (1.31, 8.41)** | .0116 |
| Bae[41] | 2021 | South Korea | 1760 | 1232 | <70 yrs (n = NR) | ≥70 yrs (n = NR) | Mortality | **2.49 (1.55, 4.01)** | <.001 |
| Byeon[42] | 2021 | South Korea | 12 646 | 12 646 | ≤49 yrs (n = 7075) | 50–59 yrs (n = 2266) | Mortality | **8.72 (3.20, 23.8)** | NR |
|  |  |  |  |  |  | 60–69 yrs (n = 1743) |  | **19.4 (7.5, 49.8)** | NR |
|  |  |  |  |  |  | 70–79 yrs (n = 806) |  | **64.8 (25.5, 165)** | NR |
|  |  |  |  |  |  | 80+ (n = 453) |  | **151 (58.7, 388)** | NR |
| Chang[43] | 2020 | South Korea | 106 | 106 | Younger age (n = NR) | Older age (n = NR) | Mortality | **1.06 (1.01, 1.11)** | .013 |
| Chang[44] | 2020 | South Korea | 211 | 211 | Younger age (n = NR) | Older age (n = NR) | Severe COVID-19 | 1.06 (0.97, 1.16) | .192 |
| Chang[45] | 2022 | South Korea | 3887 | 3887 | <60 yrs (n = 2367) | ≥60 yrs (n = 1520) | Severe COVID-19 | **3.89 (2.81, 5.37)** | <.001 |
| Chang[46] | 2022 | South Korea | 3122 | 3122 | <60 yrs (n = 1890) | ≥60 yrs (n = 1232) | Severe COVID-19 | **3.62 (2.49, 5.27)** | <.001 |
| Cho[47] | 2021 | South Korea | 7590 | 7590 | Younger age (n = NR) | Older age per 10-yr increase (n = NR) | Mortality | **2.80 (2.40, 3.27)** | <.001 |
| Cho[48] | 2021 | South Korea | 5594 | 3729 | <50 yrs (n = 1782) | 50–69 yrs (n = 1373) | Overall survival | **6.70 (1.09, 43.9)** | .047 |
|  |  |  |  |  |  | ≥70 yrs (n = 574) |  | **26.0 (4.26, 170)** | <.001 |
| Choi[49] | 2021 | South Korea | 7590 | 7590 | Younger age (n = NR) | Older age (n = NR) | Mortality | **1.11 (1.1, 1.13)** | <.001 |
|  |  |  |  |  |  |  | ICU admission | **1.05 (1.04, 1.06)** | <.001 |
| Chung[50] | 2020 | South Korea | 110 | 110 | <70 yrs (n = NR) | ≥70 yrs (n = NR) | Severe + critical outcome^j^ | **7.11 (1.83, 27.6)** | .005 |
| Her[51] | 2021 | South Korea | 5628 | 3940 | All other age groups | 60–69 yrs (n = 654) | Mortality | **3.63 (1.64, 8.01)** | .001 |
|  |  |  |  |  |  | 70–79 yrs (n = 368) |  | **6.12 (2.84, 13.2)** | <.001 |
|  |  |  |  |  |  | ≥80 yrs (n = 236) |  | **21.2 (9.65, 46.74)** | <.001 |
| Huh[52] | 2021 | South Korea | 44 046 | 2805 | Younger age (n = NR) | Older age (n = NR) | Severe COVID-19 | 1.01 (1.00, 1.02) | .02 |
| Hwang[53] | 2020 | South Korea | 340 | 338 | Younger age (n = NR) | Older age (n = NR) | Severe pneumonia | **1.06 (1.03, 1.10)** | <.001 |
|  |  |  |  |  |  |  | Mortality | 1.06 (1.00, 1.13) | .063 |
| Hwang[54] | 2020 | South Korea | 103 | 103 | Younger age (n = NR) | Older age (n = NR) | Mortality | 1.06 (1.00, 1.10) | .039 |
| Jang[55] | 2020 | South Korea | 110 | 110 | <70 yrs (n = NR) | ≥70 yrs (n = NR) | Severe COVID-19 | 3.37 (0.26, 43.6) | .352 |
| Jang[56] | 2021 | South Korea | 5068 | 5068 | 0–59 yrs (n = 3376) | 60–69 yrs (n = NR) | Mortality | **4.4 (2.3, 8.3)** | NR |
|  |  |  |  |  |  | 70–79 yrs (n = NR) |  | **16.3 (9.0, 29.5)** | NR |
|  |  |  |  |  |  | ≥80 yrs (n = 315) |  | **79.4 (43.2, 146)** | NR |
|  |  |  |  |  |  | 60–69 yrs (n = NR) | Oxygen therapy | **3.2 (2.5, 4.0)** | NR |
|  |  |  |  |  |  | 70–79 yrs (n = NR) |  | **6.8 (5.2, 8.8)** | NR |
|  |  |  |  |  |  | ≥80 yrs (n = 315) |  | **18.4 (13.4, 25.3)** | NR |
| Kang[57] | 2021 | South Korea | 4141 | 3827 | Younger age (n = NR) | Older age (per 10-yr increase) (n = NR) | Severe COVID-19 | **2.64 (2.20, 3.15)** | NR |
|  |  |  |  |  |  |  | Mortality | **3.04 (2.45, 3.78)** | NR |
| Kang[58] | 2020 | South Korea | 7341 | 7341 | Younger age (n = NR) | Older age (n = NR) | Mortality | **1.10 (1.09, 1.11)** | <.001 |
|  |  |  |  |  |  |  | Conventional oxygen therapy | **1.07 (1.06, 1.07)** | <.001 |
|  |  |  |  |  |  |  | IMV | **1.06 (1.04, 1.07)** | <.001 |
|  |  |  |  |  |  |  | ECMO | 1.03 (1.00, 1.06) | .105 |
|  |  |  |  |  |  |  | Cardiac arrest | **1.06 (1.04, 1.09)** | <.001 |
|  |  |  |  |  |  |  | Myocardial infraction | 1.00 (1.00, 1.01) | .491 |
|  |  |  |  |  |  |  | Acute heart failure | **1.03 (1.02, 1.04)** | <.001 |
|  |  |  |  |  |  |  | Acute kidney injury | **1.03 (1.01, 1.06)** | .025 |
| Kim[59] | 2020 | South Korea | 9148 | 9148 | 0–49 yrs (n = 5239) | 50–64 yrs (n = 2435) | Mortality | **20.3 (4.72, 87.6)** | NR |
|  |  |  |  |  |  | 65–74 yrs (n = 763) |  | **61.3 (14.2, 264)** | NR |
|  |  |  |  |  |  | ≥75 yrs (n = 711) |  | **377 (92.3, 1542)** | NR |
| Kim[60] | 2021 | South Korea | 75 527 | 1911 | <60 yrs (n = 665) | 61–69 yrs (n = 514) | Severity grade 1 | **2.89 (2.13, 3.93)** | .0685 |
|  |  |  |  |  |  | 70–79 yrs (n = 418) |  | **4.76 (3.47, 6.53)** | .0002 |
|  |  |  |  |  |  | ≥80 yrs (n = 314) |  | **9.69 (6.90, 13.6)** | <.0001 |
|  |  |  |  |  |  | 61–69 yrs (n = 514) | Severity grade 2 | **3.48 (1.84, 6.58)** | .0175 |
|  |  |  |  |  |  | 70–79 yrs (n = 418) |  | **7.48 (4.05, 13.8)** | .0045 |
|  |  |  |  |  |  | ≥80 yrs (n = 314) |  | **24.7 (13.5, 45.1)** | <.0001 |
|  |  |  |  |  |  | 61–69 yrs (n = 514) | Mortality | **3.38 (1.53, 7.46)** | .0033 |
|  |  |  |  |  |  | 70–79 yrs (n = 418) |  | **9.99 (4.76, 21)** | .0008 |
|  |  |  |  |  |  | ≥80 yrs (n = 314) |  | **35.9 (17.3, 74.4)** | <.0001 |
| Kim[61] | 2022 | South Korea | 5624 | 5624 | ≥80 yrs (n = 324) | <60 yrs (n = 3839) | Mortality | **33.4 (12.8, 87.6)** | <.001 |
|  |  |  |  |  |  | 60–69 yrs (n = 916) |  | **10.7 (4.66, 24.7)** | <.001 |
|  |  |  |  |  |  | 70–79 yrs (n = 545) |  | **4.88 (2.39, 9.94)** | <.001 |
|  |  |  |  |  |  | <60 yrs (n = 3839) | Clinical severity score | **2.16 (5.90, 12.6)** | <.001 |
|  |  |  |  |  |  | 60–69 yrs (n = 916) |  | **1.66 (3.52, 7.83)** | <.001 |
|  |  |  |  |  |  | 70–79 yrs (n = 545) |  | **1.13 (2.07, 4.59)** | <.001 |
| Kim[62] | 2020 | South Korea | 2959 | 2959 | Younger age (n = NR) | Older age (n = NR) | Severe COVID-19 | **1.88 (1.44, 2.46)** | <.001 |
| Lee[63] | 2023 | South Korea | 467 | 467 | Younger age (n = NR) | Older age (n = NR) | Mortality | **1.17 (1.12, 1.22)** | <.001 |
| Lee[64] | 2020 | South Korea | 5061 | 5061 | <65 yrs (n = NR) | ≥65 yrs (n = NR) | Mortality | **14.3 (8.24, 24.9)** | <.001 |
| Lee[65] | 2020 | South Korea | 561 | 421 | <50 yrs (n = 142) | 50–59 yrs (n = 100) | Severe pneumonia | 6.63 (0.75, 59) | .09 |
|  |  |  |  |  |  | 60–69 yrs (n = 87) |  | 7.93 (0.91, 69.4) | .061 |
|  |  |  |  |  |  | 70–79 yrs (n = 68) |  | **25 (2.91, 215)** | .003 |
|  |  |  |  |  |  | ≥80 yrs (n = 24) |  | **69.3 (7.45, 645)** | <.001 |
| Lee[66] | 2020 | South Korea | 4742 | 4742 | Younger age (n = NR) | Older age (n = NR) | ICU admission | **1.04 (1.03, 1.05)** | <.05 |
|  |  |  |  | 80^k^ |  |  | Ventilator usage | **1.07 (1.01, 1.13)** | <.05 |
| Lee[67] | 2023 | South Korea | 584 | 584 | Younger age (n = NR) | Older age (n = NR) | Mortality | **1.15 (1.09, 1.22)** | <.001 |
| Lee[68] | 2020 | South Korea | 7272 | 7272 | Younger age (n = NR) | Older age (n = NR) | Respiratory failure | **1.06 (1.05, 1.07)** | <.001 |
|  |  |  |  |  |  |  | Mortality | **1.12 (1.11, 1.14)** | <.001 |
| Lee[69] | 2020 | South Korea | 4610 | 4610 | 40–59 yrs (n = 2478) | ≥60 yrs (n = 2132) | Respiratory failure | **5.05 (2.99, 8.52)** | NR |
|  |  |  |  |  |  |  | Mortality | **12.0 (7.03, 20.5)** | NR |
| Lee[70] | 2020 | South Korea | 7339 | 7339 | Younger age (n = NR) | Older age (n = NR) | Severe COVID-19 | **1.05 (1.04, 1.06)** | <.001 |
|  |  |  |  |  |  |  | Mortality | **1.12 (1.11, 1.14)** | <.001 |
| Lee[71] | 2020 | South Korea | 1005 | 1005 | <64 yrs (n = 598) | ≥65 yrs (n = 407) | Severe COVID-19 (pneumonia) | **4.32 (2.73, 6.84)** | <.001 |
|  |  |  |  |  |  |  | Mortality | **4.96 (2.65, 9.30)** | <.001 |
| Lee[72] | 2022 | South Korea | 129 128 | 8080 | 0–59 yrs (n = 5856) | 60–69 yrs (n = 1200) | ICU admission | **2.34 (1.93, 2.83)** | <.0001 |
|  |  |  |  |  |  | 70–79 yrs (n = 618) |  | **3.69 (2.93, 4.63)** | <.0001 |
|  |  |  |  |  |  | ≥80 yrs (n = 406) |  | **4.9 (3.73, 6.43)** | <.0001 |
|  |  |  |  |  |  | 60–69 yrs (n = 1200) | Mortality | **6.83 (3.91, 11.9)** | <.0001 |
|  |  |  |  |  |  | 70–79 yrs (n = 618) |  | **24.1 (14.2, 40.8)** | <.0001 |
|  |  |  |  |  |  | ≥80 yrs (n = 406) |  | **83.7 (49.4, 142)** | <.0001 |
| Lim[73] | 2021 | South Korea | 146 | 146 | Younger age (n = NR) | Older age (n = NR) | Mortality | 1.07 (1.00, 1.14) | .046 |
| Moon[74] | 2021 | South Korea | 5626 | 4426 | <70 yrs (n = 3838) | ≥70 yrs (n = 588) | Mortality | **16.6 (8.65, 31.7)** | <.001 |
|  |  |  |  |  |  |  | 30-day and 60-day survival | **13.3 (7.11, 24.7)** | <.001 |
| Moon[75] | 2020 | South Korea | 352 | 352 | <70 yrs (n = 255) | ≥70 yrs (n = 97) | Mortality | **18.3 (3.42, 97.6)** | .001 |
| Oh[76] | 2022 | South Korea | 5077 | 2106^l^ | 0–39 yrs (n = 853) | 40–69 yrs (n = 929) | Clinical severity | **8.80 (13.8, 127)** | .001 |
|  |  |  |  |  |  | ≥70 yrs (n = 324) |  | 41.94 (NR) | .001 |
|  |  |  |  | 2971^m^ | 0–39 yrs (n = 848) | 40–69 yrs (n = 1604) |  | 2.21 (0.74, 6.56) | NR |
|  |  |  |  |  |  | ≥70 yrs (n = 519) |  | **11.8 (3.97, 35.3)** | .001 |
| Oh[77] | 2021 | South Korea | 122 040 | 7780 | Younger age (n = NR) | Older age (per 10-yr increase) (n = NR) | Mortality | **2.85 (2.40, 3.38)** | <.001 |
| Oh[78]] | 2021 | South Korea | 122 040 | 7669 | Younger age (n = NR) | Older age (per 10-yr increase) (n = NR) | Mortality | **3.23 (2.70, 3.86)** | <.001 |
| Paek[79] | 2020 | South Korea | 704 | 28 | Younger age (n = NR) | Older age (n = NR) | Mortality | **4.67 (1.25, 17.4)** | .022 |
| Park[80] | 2020 | Korea | 2269 | 2269 | Younger age (n = NR) | Older age (per 10 yrs increase) (n = NR) | Mortality | **2.29 (2.07, 2.53)** | <.001 |
| Park[81] | 2020 | South Korea | 1005 | 289 | Younger age (n = NR) | Older age (n = NR) | Mortality | **1.05 (1.03, 1.08)** | <.001 |
| Seon[82] | 2021 | South Korea | 7713^n^ | 7713 | 0–59 yrs (n = 5492) | 60–69 yrs (n = 1199) | Mortality | **6.92 (3.97, 12.1)** | <.001 |
|  |  |  |  |  |  | 70–79 yrs (n = 617) |  | **27.1 (16.1, 45.6)** | <.001 |
|  |  |  |  |  |  | ≥80 yrs (n = 405) |  | **97.6 (58.3, 163)** | <.001 |
| Seong [83] | 2021 | South Korea | 488 | 488 | <65 yrs (n = 170) | ≥65 yrs (n = 318) | Mortality | **1.03 (1.01, 1.05)** | .008 |
| Shin[84] | 2021 | South Korea | 5571 | 5571 | Younger age (n = NR) | Older age (NR) | Severe COVID-19 | **1.80 (1.66, 1.95)** | <.01 |
|  |  |  |  |  |  |  | Mortality | **3.44 (2.74, 4.31)** | <.01 |
| Song[85] | 2021 | South Korea | 5621 | 5621 | 0–59 yrs (n = 3842) | ≥60 yrs (n = 1779) | Mortality^f^ | **11.7 (4.66, 34.2)** | <.001 |
|  |  |  |  |  |  |  | Fatal adverse outcome^f,o^ | **3.89 (1.86, 8.69)** | <.001 |
|  |  |  |  |  |  |  | Mortality^f^ | **3.53 (1.66, 7.49)** | .001 |
|  |  |  |  |  |  |  | Fatal adverse outcome^g,o^ | **2.64 (1.44, 4.83)** | .002 |
| Wang[86] | 2021 | South Korea | 2800^p^ | 446 | <80 yrs (n = 280) | ≥80 yrs (n = 166) | Mortality | **3.40 (1.91, 6.28)** | <.001 |
| Yun[87] | 2020 | South Korea | 7590 | 7363 | 50–59 yrs (n = NR) | 20–39 yrs (n = NR) | Severe COVID-19 | 0.14 (0.05, 0.41) | <.001 |
|  |  |  |  |  |  | 40–49 yrs (n = NR) |  | 0.08 (0.01, 0.58) | .01 |
|  |  |  |  |  |  | 60–69 yrs (n = NR) |  | **2.50 (1.53, 4.11)** | <.001 |
|  |  |  |  |  |  | 70–79 yrs (n = NR) |  | **5.41 (5.41, 8.86)** | <.001 |
|  |  |  |  |  |  | >80 yrs (n = NR) |  | **15.9 (9.62, 26.2)** | <.001 |
| Huang[88] | 2022 | Taiwan | 239 | 239 | <65yrs (n = NR) | >65 yrs (n = NR) | Mortality | **5.14 (1.06, 24.7)** | .041 |
| Naorungroj [89] | 2023 | Thailand | 2430 | 2430 | <65 yrs (n = NR) | >65 yrs (n = NR) | Mortality | **14.3 (5.76, 35.5)** | <.001 |
| Do[90] | 2023 | Vietnam | 504 | 504 | <60 yrs (n = 247) | 60–70 yrs (n = NR) | Mortality | **2.94 (1.81, 4.79)** | .000 |
|  |  |  |  |  | <70 yrs (n = NR) | ≥71 yrs (n = NR) |  | **3.8 (1.74, 8.3)** | .001 |

Abbreviations: ECMO, extracorporeal membrane oxygenation; ICU, intensive care unit; NR, not reported; IMV, invasive mechanical ventilation; MV, mechanical ventilation; NR, not reported.

^a^Paediatric and adult patients. ^b^Residential aged care facility residents. ^c^Hospitalization or death. ^d^ICU admission or death. ^e^Grade 2: High flow oxygen or non-invasive positive pressure ventilation, Grade 3/4: IMV and ECMO. ^f^Multivariable logistic regression. ^g^Cox regression analysis. ^h^Grade 5: Death; ^i^Aged ≥60 years. ^j^Composite outcome. ^k^ICU admitted cohort. ^l^Male; ^m^Female. ^n^Patients with mental illness. ^o^IMV, multiorgan failure, ECMO, and death. ^p^Aged ≥50 years.

### Supplementary Table 2. Studies assessing sex

| **First author** | **Year** | **Country** | **Total** | **Sample** | **Reference**  **(sample size)** | **Comparator**  **(sample size)** | **Outcome** | **OR/HR (95% CI)** | ***P*-value** |
| --- | --- | --- | --- | --- | --- | --- | --- | --- | --- |
| Ellis[3] | 2023 | Australia | 1071^a^ | 1071 | Female (n = 597) | Male (n = 474) | Hospitalization | **1.7 (1.2, 2.4)** | NR |
|  |  |  |  |  |  |  | Mortality | **2.5 (1.7, 3.6)** | NR |
| Liu[4] | 2021 | Australia | 4054 | 4054 | Female (n = 2019) | Male (n = 2035) | Severe COVID-19^b^ | 1.11 (0.92, 1.33) | NR |
|  |  |  |  |  |  |  | Very severe COVID-19^c^ | **1.40 (1.04, 1.88)** | NR |
| Muleme[5] | 2023 | Australia | 2876^d^ | 2876 | Female (n = NR) | Male (n = NR) | Hospitalization | **1.32 (1.04, 1.68)** | .02 |
|  |  |  |  |  |  |  | Mortality | **2.05 (1.63, 2.58)** | < .001 |
| Chung[8] | 2021 | Hong Kong | 3074 | 3074 | Female (n = 1593) | Male (n = 1481) | Severe COVID-19 | **2.91 (2.16, 3.92)** | < .001 |
| Wong[11] | 2023 | Hong Kong | 32 222 | 22 604 | Male (n = 12 845) | Female (n = 9759) | Mortality | **0.76 (0.72, 0.81)** | NR |
| Zhou[13] | 2023 | Hong Kong | 6089 | 6089 | Female (n = 3059) | Male (n = 3030) | Severe COVID-19 | **1.79 (1.34, 2.38)** | .0001 |
| Zhou[14] | 2020 | Hong Kong | 1043 | 1043 | Female (n = 480) | Male (n = 563) | ICU admission | 2.42 (0.87, 6.78) | <.0001 |
| Fukushima[16] | 2021 | Japan | 234 | 234 | Female (n = 80) | Male (n = 154) | Critical events | **15.6 (2.64, 300)** | NR |
| Fukushima[17] | 2023 | Japan | 2430 | 2430 | Female (n = 753) | Male (n = 1677) | Critical events | **1.67 (1.14, 2.45)** | .008 |
| Ito[18] | 2023 | Japan | 936 | 936 | Female (n = 362) | Male (n = 574) | Hospitalization | 1.45 (0.79, 2.65) | .231 |
| Matsushita[19] | 2022 | Japan | 23 414 | 23 414 | Male (n = 13 360) | Female (n = 10 054) | COVID severity grade 2/3/4/5^e^ | **2.32 (2.02, 2.66)** | NR |
|  |  |  |  |  |  |  | COVID severity grade 3/4/5^e^ | **2.39 (2.04, 2.80)** | NR |
|  |  |  |  |  |  |  | COVID severity grade 5^e^ | **2.20 (1.84, 2.63)** | NR |
| Miyashita[20] | 2022 | Japan | 937 758 | 937 758 | Female (n = 426 723) | Male (n = 511 035) | Severe COVID-19 | **2.08 (2.03, 2.13)** | <.001 |
|  |  |  |  |  |  |  | Mortality | **1.75 (1.69, 1.81)** | <.001 |
| Muto[21] | 2021 | Japan | 300 | 300 | Female (n = 113) | Male (n = 187) | Critical/severe COVID-19 | 1.65 (0.81, 3.40) | .17 |
| Nishida[23] | 2022 | Japan | 393 | 393 | Female (n = 138) | Male (n = 255) | Mortality | 1.30 (0.37, 4.94) | .681 |
|  |  |  |  |  |  |  | IMV | **5.10 (1.35, 33.4)** | .036 |
|  |  |  |  |  |  |  | Mortality or IMV | 1.93 (0.73, 5.76) | .204 |
|  |  |  |  |  |  |  | Mortality | 0.76 (0.21, 2.79) | .678 |
|  |  |  |  |  |  |  | IMV | **4.53 (1.02, 20.2)** | .048 |
|  |  |  |  |  |  |  | Mortality or IMV | 1.68 (0.64, 4.39) | .292 |
| Nishimura[24] | 2023 | Japan | 30 130 | 483 | Female (n = 123) | Male (n = 361) | Hospitalization | 1.09 (0.78, 1.53) | .612 |
|  |  |  |  | 461 | Female (n = 103) | Male (n = 357) | Oxygen administration | 1.18 (0.82, 1.69) | .377 |
| Nojiri[91] | 2023 | Japan | 11 440 | 11 440 | Female (n = 8001) | Male (n = 3439) | Severe COVID-19 | **1.85 (1.67, 2.06)** | <.001 |
|  |  |  |  |  |  |  | Mortality | **2.4 (1.85, 3.14)** | <.001 |
| Numaguchi[25] | 2022 | Japan | 415 | 415 | Female (n = 201) | Male (n = 214) | Severe COVID-19 | **1.88 (1.14, 3.11)** | .013 |
| Sakamoto[28] | 2022 | Japan | 6176 | 6176 | Female (n = 2493) | Male (n = 3683) | Hospitalization | **1.81 (1.35, 2.42)** | NR |
| Sato[29] | 2022 | Japan | 500 | 500 | Female (n = 194) | Male (n = 306) | Mortality or ECMO or IMV or MV or ICU admission | 1.87 (0.82, 4.29) | .14 |
| Takeyama[30] | 2022 | Japan | 2894 | 2894 | Female (n = | Men (n = 1885) | Mortality | 1.02 (0.67, 1.55) | .92 |
| Tanaka[31] | 2021 | Japan | 3192^f^ | 3192 | Female (n = 1538) | Male (n = 1654) | Mortality | **1.56 (1.20, 2.03)** | NR |
| Yamada[32] | 2021 | Japan | 6873 | 1891^g^ | Female (n = NR) | Male (n = NR) | Oxygen therapy | **2.83 (1.34, 6.01)** | .007 |
|  |  |  |  | 1615^h^ |  |  |  | **1.67 (1.23, 2.28)** | .001 |
| Jefferies[33] | 2020 | New Zealand | 1503 | 1495 | Female (n = 831) | Male (n = 664) | Severe COVID-19 | 0.86 (0.55, 1.35) | NR |
| Espiritu[35] | 2021 | Philippine | 10 881 | 10 881 | Female (n = 5099) | Men (n = 5780) | Mortality | 0.96 (0.87, 1.06) | .405 |
|  |  |  |  |  |  |  | Respiratory failure | 0.85 (0.77, 0.95) | .003 |
|  |  |  |  |  |  |  | ICU admission | 0.88 (0.80, 0.98) | .016 |
| Koh[36] | 2021 | Singapore | 1042 | 1042 | Female (n = 48) | Male (n = 994) | Severe COVID-19 | 0.79 (0.34, 1.81) | .571 |
| Lim[37] | 2021 | Singapore | 249 | 249 | Female (n = NR)* *Clinical Frailty Scale | Male (n = NR)* *Clinical Frailty Scale | Severe COVID-19 | **5.94 (1.47, 24)** | .012 |
|  |  |  |  |  | Female (n = NR)* *Frailty Index | Male (n = NR)* *Frailty Index |  | **4.54 (1.25, 16.5)** | .022 |
| Bae[41] | 2021 | South Korea | 1760 | 1232 | Male (n = 750) | Female (n = 482) | Mortality | 1.33 (0.09, 1.96) | .149 |
| Byeon[42] | 2021 | South Korea | 12 646 | 12 646 | Female (n = 7146) | Male (n = 5197) | Mortality | **2.25 (1.77, 2.86)** | NR |
| Chang[45] | 2022 | South Korea | 3887 | 3887 | Female (n = 2436) | Male (n = 1451) | Severe COVID-19 | **2.40 (1.72, 3.34)** | <.001 |
| Chang[46] | 2022 | South Korea | 3122 | 3122 | Female (n = 2163) | Male (n = 959) | Severe COVID-19 | **1.15 (1.47, 3.13)** | <.001 |
| Cho[47] | 2021 | South Korea | 7590 | 7590 | Female (n = 4495) | Male (n = 3095) | Mortality | 0.46 (0.34, 0.63) | <.001 |
| Cho[48] | 2021 | South Korea | 5594 | 3729 | Female (n = 2193) | Male (n = 1536) | Overall survival | 1.35 (0.85, 2.15) | .300 |
| Cho[92] | 2021 | South Korea | 1272^f^ | 1272 | Female (n = 750) | Male (n = 522) | Mortality | **2.28 (1.49, 3.52)** | NR |
|  |  |  |  |  |  |  | Severe COVID-19 | **2.61 (1.76, 3.93)** | NR |
| Choi[49] | 2021 | South Korea | 7590 | 7590 | Male (n = 3095) | Female (n = 4495) | Mortality | **0.39 (0.28, 0.53)** | <.001 |
|  |  |  |  |  |  |  | ICU admission | **0.52 (0.39, 0.69)** | <.001 |
| Her[51] | 2021 | South Korea | 5628 | 3940 | Female (n = 2318) | Male (n = 1622) | Mortality | **1.67 (1.04, 2.67)** | .034 |
| Her[93] | 2022 | South Korea | 5628 | 5628 | Female (n = 3308) | Male (n = 2320) | No limit of activity | 1.10 (0.99, 1.22) | .077 |
|  |  |  |  |  |  |  | Limit of activity but no oxygen | 1.10 (0.74, 1.64) | .625 |
|  |  |  |  |  |  |  | Oxygen with nasal prong | 0.87 (0.67, 1.13) | .306 |
|  |  |  |  |  |  |  | Oxygen with facial mask | 0.73 (0.30, 1.81) | .497 |
|  |  |  |  |  |  |  | Non-invasive ventilation | 1.66 (0.60, 4.60) | .334 |
|  |  |  |  |  |  |  | IMV | 0.38 (0.08, 1.80) | .224 |
|  |  |  |  |  |  |  | Multi-organ failure/ECMO | Inf (0 .00–Inf) | .841 |
|  |  |  |  |  |  |  | Mortality | 0.59 (0.41, 0.85) | .023 |
| Huh[52] | 2021 | South Korea | 44 046 | 2805 | Female (n = 1523) | Male (n = 1282) | Severe COVID-19 | 1.09 (0.92, 1.29) | .35 |
| Hwang[53] | 2020 | South Korea | 340^i^ | 338 | Female (n = 209) | Male (n = 129) | Severe pneumonia | **2.38 (1.49, 3.81)** | <.001 |
|  |  |  |  | 340 | Female (n = 210) | Male (n = 130) | Mortality | **2.58 (1.38, 4.81)** | .003 |
| Jang[55] | 2020 | South Korea | 110 | 110 | Female (n = 62) | Male (n = 48) | Severe COVID-19 | 2.98 (0.23, 38.8) | .404 |
| Jang[56] | 2021 | South Korea | 5068 | 5068 | Female (n = 2102) | Male (n = 2966) | Mortality | **2.3 (1.6, 3.1)** | NR |
|  |  |  |  |  |  |  | Need for oxygen therapy | **1.6 (1.3, 1.9)** | NR |
| Kang[57] | 2021 | South Korea | 4141 | 3827 | Male (n = 1726) | Female (n = 2415) | Severe COVID-19 | **2.85 (1.94, 4.19)** | NR |
|  |  |  |  |  |  |  | Mortality | **2.92 (1.90, 4.48)** | NR |
| Kang[58] | 2020 | South Korea | 7341 | 7341 | Male (n = 4371) | Female (n = 2970) | Mortality | **0.48 (0.37, 0.63)** | <.001 |
|  |  |  |  |  |  |  | Conventional oxygen therapy | **0.59 (0.51, 0.69)** | <.001 |
|  |  |  |  |  |  |  | IMV | **0.44 (0.31, 0.64)** | <.001 |
|  |  |  |  |  |  |  | ECMO | 0.58 (0.24, 1.39) | .22 |
|  |  |  |  |  |  |  | Cardiac arrest | **0.28 (0.14. 0.54)** | <.001 |
|  |  |  |  |  |  |  | Myocardial infraction | **0.74 (0.58, 0.95)** | .018 |
|  |  |  |  |  |  |  | Acute heart failure | 0.91 (0.74, 1.13) | .409 |
|  |  |  |  |  |  |  | Acute kidney injury | **0.38 (0.17, 0.83)** | .017 |
| Kim[94] | 2021 | South Korea | 5621 | 5621 | Female (n = 3304) | Male (n = 2317) | Severe COVID-19 | **1.76 (1.32, 2.34)** | <.001 |
|  |  |  |  |  |  |  | Mortality | **1.55 (1.05, 2.29)** | .027 |
| Kim[59] | 2020 | South Korea | 9148 | 9148 | Female (n = 5529) | Male (n = 3556) | Mortality | **2.18 (1.50, 3.17)** | NR |
| Kim[60] | 2021 | South Korea | 75 527 | 1911 | Male (n = 844) | Female (n = 1067) | Severity grade 1 | **0.66 (0.53, 0.82)** | .0001 |
|  |  |  |  |  |  |  | Severity grade 2 | **0.52 (0.38, 0.72)** | <.0001 |
|  |  |  |  |  |  |  | Mortality | **0.51 (0.36, 0.73)** | .0002 |
| Kim[62] | 2020 | South Korea | 2959 | 2959 | Female (n = 1780) | Male (n = 1179) | Severe COVID-19 | 0.58 (0.30, 1.12) | NR |
| Lee[64] | 2020 | South Korea | 5061 | 5061 | Male (n = 2229) | Female (n = 2832) | Mortality | **0.85 (0.55, 1.33)** | .482 |
| Lee[65] | 2020 | South Korea | 561 | 421 | Female (n = 304) | Male (n = 117) | Severe pneumonia | **3.20 (1.53, 6.77)** | .002 |
| Lee[66] | 2020 | South Korea | 4742 | 4742 | Female (n = 2586) | Male (n = 2156) | ICU admission | **2.08 (1.30, 3.30)** | <.05 |
|  |  |  |  | 80^j^ | Female (n = 30) | Male (n = 50) | Ventilator usage | 3.25 (0.69, 15.4) | NR |
| Lee[67] | 2023 | South Korea | 584 | 584 | Female (n = 305) | Male (n = 279) | Mortality | 1.16 (0.35, 3.87) | .809 |
| Lee[68] | 2020 | South Korea | 7272 | 7272 | Female (n = 4345) | Male (n = 2927) | Respiratory failure | **2.31 (1.58, 3.37)** | <.001 |
|  |  |  |  |  |  |  | Mortality | **2.46 (1.82, 3.33)** | <.001 |
| Lee[69] | 2020 | South Korea | 4610 | 4610 | Female (n = 3900) | Male (n = 1710) | Respiratory failure | **2.06 (1.42, 3.01)** | NR |
|  |  |  |  |  |  |  | Mortality | **1.74 (1.31, 2.30)** | NR |
| Lee[70] | 2020 | South Korea | 7339 | 7339 | Female (n = 4369) | Male (n = 2970) | Severe COVID-19 | **1.60 (1.34, 1.91)** | <.001 |
|  |  |  |  |  |  |  | Mortality | **2.10 (1.51, 2.92)** | <.001 |
| Lee[72] | 2022 | South Korea | 129 128 | 8080 | Male (n = 3244) | Female (n = 4836) | ICU admission | **0.67 (0.58, 0.77)** | <.0001 |
|  |  |  |  |  |  |  | Mortality | **0.41 (0.31, 0.55)** | <.0001 |
| Moon[74] | 2021 | South Korea | 5626 | 4426 | Female (n = 2563) | Male (n = 1863) | Mortality | **2.93 (1.70, 5.06)** | <.001 |
|  |  |  |  |  |  |  | 30-day and 60-day survival | **2.31 (1.46, 3.63)** | <.001 |
| Oh[77] | 2021 | South Korea | 122 040 | 7780 | Female (n = NR) | Male (n = NR) | Mortality | **2.12 (1.55, 2.88)** | <.001 |
| Oh[78] | 2021 | South Korea | 122 040 | 7669 | Female (n = 4645) | Male (n = 3024) | Mortality | **2.48 (1.81, 3.41)** | <.001 |
| Paek[79] | 2020 | South Korea | 704 | 28 | Female (n = 12) | Male (n = 16) | Mortality | 1.06 (0.36, 3.17) | .914 |
| Park[80] | 2020 | Korea | 2269 | 2269 | Female (n = 1455) | Male (n = 814) | Mortality | **2.03 (1.29, 3.20)** | .002 |
| Park[95] | 2021 | Korea | 5621 | 5621 | Female (n = 3304) | Male (n = 2317) | ICU admission | **2.45 (1.63, 3.67)** | <.001 |
|  |  |  |  | 4172^k^ | Female (n = 2556) | Male (n = 1616) | Mortality | **1.55 (1.05, 2.29)** | .03 |
| Seon[82] | 2021 | South Korea | 7713^l^ | 7713 | Male (n = 2304) | Female (n = 4665) | Mortality | **0.40 (0.30, 0.54)** | <.001 |
| Shin[84] | 2021 | South Korea | 5571 | 5571 | Male (n = 2304) | Female (n = 3267) | Severe COVID-19 | **1.39 (1.11, 1.72)** | <.01 |
|  |  |  |  |  |  |  | Mortality | **3.06 (1.99, 4.72)** | <.01 |
| Song[85] | 2021 | South Korea | 5621 | 5621 | Female (n = 3304) | Male (n = 2317) | Mortality^m^ | 1.82 (0.91, 3.65) | .089 |
|  |  |  |  |  |  |  | Fatal adverse outcome^m,n^ | 1.80 (0.97, 3.38) | .063 |
|  |  |  |  |  |  |  | Mortality^o^ | 1.26 (0.79, 2.02) | .337 |
|  |  |  |  |  |  |  | Fatal adverse outcome^n,o^ | 1.33 (0.86, 2.04) | .198 |
| Yun[87] | 2020 | South Korea | 7590 | 7363 | Male (n = 2974) | Female (n = 4389) | Severe COVID-19 | **0.43 (0.33, 0.57)** | <.001 |

Abbreviations: ECMO, extracorporeal membrane oxygenation; ICU, intensive care unit; IMV, invasive mechanical ventilation; MV, mechanical ventilation; NR, not reported.

^a^Paediatric and adult patients. ^b^Hospitalization or death. ^c^ICU or death. ^d^Residential aged care facility residents. ^e^Grade 2: High-flow oxygen or non-invasive positive pressure ventilation, Grade 3/4: IMV and ECMO, Grade 5: Death. ^f^Aged ≥60 years. ^g^Aged 18–39 years. ^h^Aged 40–64 years. ^i^Aged ≥65 years. ^j^ICU admitted cohort. kPatients who are Symptomatic patints. ^l^Patients with mental illness. ^m^Multivariable logistic regression. ^n^IMV, multiorgan failure, ECMO and death. ^o^Cox regression analysis.

### Supplementary Table 3. Studies assessing body mass index ≥25 kg/m^2^/obesity

| **First author** | **Year** | **Country** | **Total** | **Cohort size** | **Reference  (sample size)** | **Comparator  (sample size)** | **Outcome** | **OR/HR (95% CI)** | ***P*-value** |
| --- | --- | --- | --- | --- | --- | --- | --- | --- | --- |
| Davis[2] | 2022 | Australia | 794^a^ | 794 | No obesity (n = 778) | Obesity (n = 16) | Hospitalization | **4.41 (1.26, 15.5)** | .02 |
| Ellis[3] | 2023 | Australia | 1071^b^ | 1071 | BMI <30 (n = 723) | BMI ≥30 (n = 142) | Hospitalization | 1.1 (0.6, 1.7) | NR |
|  |  |  |  |  |  |  | Mortality | 0.7 (0.4, 1.4) | NR |
| Liu[4] | 2021 | Australia | 4054 | 4054 | No obesity (n = 2421) | Obesity (n = 1633) | Severe COVID-19^c^ | 1.20 (0.95, 1.51) | NR |
|  |  |  |  |  |  |  | Very severe COVID-19^d^ | **1.52 (1.05, 2.21)** | NR |
| Zhou[13] | 2023 | Hong Kong | 6089 | 6089 | No obesity (n = 6065) | Obesity (n = 24) | Severe COVID-19 | 1.71 (0.24, 12.2) | .594 |
| Fukushima[17] | 2023 | Japan | 2430 | 2430 | BMI <25 (n = NR) | BMI ≥25 (n = NR) | Critical events | 1.17 (0.85, 1.61) | .33 |
| Ito[18] | 2023 | Japan | 936 | 936 | BMI 18.5–25.0 (n = 457) | BMI ≥25.1 (n = 142) | Hospitalization | **5.48 (1.24, 24.2)** | .025 |
| Lee[96] | 2022 | Japan | 1837 | 1837 | No obesity (n = 1108) | Class 1 obesity: 25.0 ≤ BMI <30.0 (n = 524) | Oxygen therapy | **2.01 (1.56, 2.57)** | NR |
|  |  |  |  |  |  |  | ICU admission | 1.2 (0.9, 1.47) | NR |
|  |  |  |  |  |  |  | IMV | 1.09 (0.78, 1.52) | NR |
|  |  |  |  |  |  |  | Severe COVID-19 | 1.14 (0.88, 1.45) | NR |
|  |  |  |  |  |  | Class 2 obesity: 30.0 ≤ BMI <35.0 (n = 147) | Oxygen therapy | **4.75 (3.08, 7.32)** | NR |
|  |  |  |  |  |  |  | ICU admission | **1.99 (1.32, 2.98)** | NR |
|  |  |  |  |  |  |  | IMV | **1.68 (1.00, 2.83)** | NR |
|  |  |  |  |  |  |  | Severe COVID-19 | **1.81 (1.21, 2.70)** | NR |
|  |  |  |  |  |  | Class 3/4 obesity: BMI ≥35.0 (n = 58) | Oxygen therapy | **3.12 (1.68, 5.77)** | NR |
|  |  |  |  |  |  |  | ICU admission | 1.55 (0.85, 2.86) | NR |
|  |  |  |  |  |  |  | IMV | 1.52 (0.68, 3.39) | NR |
|  |  |  |  |  |  |  | Severe COVID-19 | 1.64 (0.90, 2.91) | NR |
| Ninomiya[22] | 2021 | Japan | 210 | 210 | Overweight (BMI <25) (n = 139) | Overweight (BMI ≥25) (n = 71) | Worsened COVID-19/  oxygen therapy | **3.26 (1.46, 7.27)** | .0038 |
| Nishimura[24] | 2023 | Japan | 30 130 | 1097 | No obesity (BMI <30) (n = 462) | Obesity (BMI ≥30) (n = 21) | Hospitalization | 1.01 (0.47, 2.16) | .99 |
|  |  |  |  |  |  |  | Oxygen administration | 1.7 (0.77, 3.72) | .189 |
| Numaguchi[25] | 2022 | Japan | 415 | 415 | Lower BMI (n = NR) | Higher BMI (n = NR) | Severe COVID-19  (severe or worse) | **1.37 (1.05, 1.79)** | .02 |
| Ogihara[97] | 2022 | Japan | 2690 | 2690 | No obesity (BMI <30) (n = 2233) | Obesity (BMI ≥30) (n = 457) | Mortality or IMV or ECMO | **1.85 (1.39, 2.47)** | <.01 |
| Okauchi[26] | 2021 | Japan | 84 | 84 | (BMI <25) (n = NR) | (BMI ≥25 kg/m²) (n = NR) | Oxygen supplementation | **26.0 (3.54, 190)** | .001 |
| Sakamoto[28] | 2022 | Japan | 6176 | 6176 | BMI 18.5–25.0 (n = 4144) | BMI | Hospitalization | **1.69 (1.28, 2.24)** | NR |
|  |  |  |  |  |  | ≥30 (n = 270) |  | **1.81 (1.15, 2.83)** | NR |
| Yamada[32] | 2021 | Japan | 6873 | 1891^e^ | BMI 18.5–22.9 (n = NR) | BMI 23.0–24.9 (n = NR) | Oxygen therapy | 1.93 (0.80, 4.70) | .146 |
|  |  |  |  |  |  | BMI 25.0–29.9 (n = NR) |  | 2.08 (0.96, 4.52) | .064 |
|  |  |  |  |  |  | BMI ≥30.0 (n = NR) |  | **6.85 (3.18, 14.8**) | <.001 |
|  |  |  |  | 1615^f^ |  | BMI 23.0–24.9 (n = NR) |  | 1.17 (0.77, 1.79) | .457 |
|  |  |  |  |  |  | BMI 25.0–29.9 (n = NR) |  | **2.21 (1.54, 3.17)** | <.001 |
|  |  |  |  |  |  | BMI ≥30.0 (n = NR) |  | **2.57 (1.69, 3.91)** | <.001 |
|  |  |  |  | 1007^g^ |  | BMI 23.0–24.9 (n = NR) |  | 1.00 (0.68, 1.48) | .999 |
|  |  |  |  |  |  | BMI 25.0–29.9 (n = NR) |  | **2.02 (1.38, 2.94)** | <.001 |
|  |  |  |  |  |  | BMI ≥30.0 g/m² (n = NR) |  | 1.77 (0.96, 3.27) | .069 |
| Espiritu[98] | 2022 | Philippines | 4463 | 4463 | BMI 18.5–24.9 (n = 2100) | BMI 25–29.9 (n = 1463) | Mortality | 1.08 (0.71, 1.64) | .724 |
|  |  |  |  |  |  | BMI ≥30 (n = 749) |  | 1.12 (0.72, 1.75) | .605 |
|  |  |  |  |  |  | BMI 25–29.9 (n = 1463) | IMV | 1.00 (0.85, 1.17) | .972 |
|  |  |  |  |  |  | BMI ≥30 (n = 749) |  | 1.16 (0.95, 1.41) | .147 |
|  |  |  |  |  |  | BMI 25–29.9 (n = 1463) | Disease severity at nadir | **1.19 (1.02, 1.38)** | .024 |
|  |  |  |  |  |  | BMI ≥30 (n = 749) |  | **1.46 (1.22, 1.76)** | <.001 |
|  |  |  |  |  |  | BMI 25–29.9 (n = 1463) | ICU admission | 0.95 (0.79, 1.15) | .624 |
|  |  |  |  |  |  | BMI ≥30 (n = 749) |  | 1.21 (0.97, 1.53) | .098 |
| Koh[36] | 2021 | Singapore | 1042 | 1042 | Low BMI | High BMI | Severe COVID-19 | **1.09 (1.02, 1.16)** | .014 |
| Chang[45] | 2022 | South Korea | 3887 | 3887 | Lower BMI (n = NR) | Higher BMI (n = NR) | Severe COVID-19 | 1.01 (0.97, 1.06) | .586 |
| Cho[92] | 2021 | South Korea | 1272 | 1272 | Normal weight  (BMI 18.5–22.9) (n = 488) | Overweight  (BMI 23.0–24.9) (n = 360) | Mortality | 0.50 (0.27, 0.91) | NR |
|  |  |  |  |  |  | Obesity class I  (BMI 25.0–29.9) (n = 326) |  | 1.13 (0.68, 1.89) | NR |
|  |  |  |  |  |  | Obesity class II  (BMI ≥30.0) (n = 31) |  | 1.15 (0.25, 3.77) | NR |
|  |  |  |  |  | Normal weight  (18.5–22.9) (n = 488) | Overweight  (BMI 23.0–24.9) (n = 360) | Severe COVID-19 | 0.55 (0.31, 0.94) | NR |
|  |  |  |  |  |  | Obesity class I  (BMI 25.0–29.9) (n = 326) |  | 1.13 (0.70, 1.83) | NR |
|  |  |  |  |  |  | Obesity class II  (BMI ≥30.0) (n = 31) |  | 1.86 (0.56, 5.16) | NR |
| Kang[57] | 2021 | South Korea | 4141 | 3827 | BMI 24.9 (n = 1005) | BMI 18.5–22.9 (n = 1741) | Severe COVID-19  (Critical illness) | 1.59 (0.94, 2.71) | NR |
|  |  |  |  |  |  | BMI 25.0–29.9 (n = 1011) |  | **2.37 (1.38, 4.07)** | NR |
|  |  |  |  |  |  | BMI ≥30 (n = 193) |  | **4.40 (1.66, 11.7)** | NR |
|  |  |  |  |  |  | BMI 18.5–22.9 (n = 1741) | Mortality | 1.59 (0.88, 2.89) | NR |
|  |  |  |  |  |  | BMI 25.0–29.9 (n = 1011) |  | **2.43 (1.32, 4.47)** | NR |
|  |  |  |  |  |  | BMI ≥30 (n = 193) |  | **4.32 (1.37, 13.6)** | NR |
| Kim[61] | 2022 | South Korea | 5624 | 5624 | BMI ≥30 (n = 208) | BMI 18.5–22.9 (n = 1867) | Mortality | 1.80 (0.48, 6.66) | NR |
|  |  |  |  |  |  | BMI 23–24.9 (n = 1038) |  | **3.98 (1.00, 15.8)** | NR |
|  |  |  |  |  |  | BMI 25–29.9 (n = 1052) |  | 1.39 (0.38, 5.08) | NR |
|  |  |  |  |  |  | BMI 18.5–22.9 (n = 1867) | Clinical Severity Score | 0.55 (1.07, 2.79) | <.05 |
|  |  |  |  |  |  | BMI 23–24.9 (n = 1038) |  | 0.58 (1.09, 2.92) | <.05 |
|  |  |  |  |  |  | BMI 25–29.9 (n = 1052) |  | 0.16 (0.73, 1.91) | NR |
| Kim[99] | 2020 | South Korea | 4069 | 4069 | BMI 20–22.9 (n = 1194) | BMI 23–24.9 (n = 1012) | Severe COVID-19 | 0.72 (0.51, 1.03) | NR |
|  |  |  |  |  |  | BMI 25–29.9 (n = 1279) |  | 1.19 (0.88, 1.60) | NR |
|  |  |  |  |  |  | BMI ≥30 (n = 191) |  | 1.16 (0.63, 2.14) | NR |
|  |  |  |  |  | Metabolically healthy normal weight (n = 1871) | Metabolically unhealthy normal weight (n = 728) | Severe COVID-19 | **1.41 (1.01, 1.98)** | NR |
|  |  |  |  |  |  | Metabolically healthy obesity (n = 595) |  | 1.48 (0.98, 2.23) | NR |
|  |  |  |  |  |  | Metabolically unhealthy obesity (n = 875) |  | **1.77 (1.29, 2.44)** | NR |
|  |  |  |  |  |  | Metabolically unhealthy normal weight (n = 728) | ICU admission | 1.45 (0.95, 2.20) | NR |
|  |  |  |  |  |  | Metabolically healthy obesity (n = 595) |  | 1.59 (0.99, 2.54) | NR |
|  |  |  |  |  |  | Metabolically unhealthy obesity (n = 875) |  | **1.92 (1.31, 2.82)** | NR |
|  |  |  |  |  |  | Metabolically unhealthy normal weight (n = 728) | Use of IMV or ECMO | 1.87 (0.95, 3.65) | NR |
|  |  |  |  |  |  | Metabolically healthy obesity (n = 595) |  | 2.02 (0.94, 4.33) | NR |
|  |  |  |  |  |  | Metabolically unhealthy obesity (n = 875) |  | **2.65 (1.42, 4.94)** | NR |
|  |  |  |  |  |  | Metabolically unhealthy normal weight (n = 728) | Mortality | **1.90 (1.13, 3.19)** | NR |
|  |  |  |  |  |  | Metabolically healthy obesity (n = 595) |  | 1.44 (0.71, 2.94) | NR |
|  |  |  |  |  |  | Metabolically unhealthy obesity (n = 875) |  | **2.22 (1.33, 3.70)** | NR |
| Kim[100] | 2020 | South Korea | 4057 | 4057 | Normal (BMI ≥18.5 to <23) (n = 1668) | Overweight (BMI ≥23 to <25) (n = 953)^h^ | Mortality | 0.80 (0.46, 1.39) | .432 |
|  |  |  |  |  |  | Obese (≥25 ) (n = 1159)^h^ |  | **1.71 (1.10, 2.66)** | .017 |
|  |  |  |  |  |  | Overweight (BMI ≥23 to <25) (n = 953)^g^ |  | 1.13 (0.86, 1.49) | .369 |
|  |  |  |  |  |  | Obese (BMI ≥25 ) (n = 1159)^i^ |  | **1.71 (1.32, 2.21)** | <.001 |
| Kim[62] | 2020 | South Korea | 2959 | 2959 | Normal BMI (n = 1216) | High BMI (n = 1743) | Severe COVID-19 | 1.12 (0.59, 2.11) | NR |
| Oh[76] | 2022 | South Korea | 5077 | 2106 | Normal weight (n = 564) | BMI overweight (n = 452) | Clinical severity | 1.08 (0.57, 2.04) | NR |
|  |  |  |  |  |  | BMI obese (n = 610) | Clinical severity | 1.73 (0.98, 3.07) | NR |
| Shin[84] | 2021 | South Korea | 5571 | 5571 | Lower BMI (n = NR) | Higher BMI (n = NR) | Severe COVID-19 | **1.27 (1.14, 1.42)** | <.01 |
|  |  |  |  |  |  |  | Mortality | 1.13 (0.91, 1.41) | .27 |
| Song[85] | 2021 | South Korea | 5621 | 5621 | BMI <25 (n = 4361) | BMI ≥25 (n = 1260) | Mortality | 0.88 (0.75, 1.05) | .093 |
|  |  |  |  |  |  |  | Fatal adverse outcome^j^ | 0.89 (0.76, 1.07) | .098 |
|  |  |  |  |  | BMI <18.5 (n = NR) | BMI 18.5–22.9 (n = NR) | Mortality | 0.31 (0.18, 0.65) | .001 |
|  |  |  |  |  |  | BMI 23.0–24.9 (n = NR) |  | 0.24 (0.11, 0.52) | <.001 |
|  |  |  |  |  |  | BMI 25.0–29.9 (n = NR) |  | 0.53 (0.26, 1.05) | .069 |
|  |  |  |  |  |  | BMI ≥30.0 (n = NR) |  | 0.56 (0.18, 1.69) | .301 |
|  |  |  |  |  |  | BMI 18.5–22.9 (n = NR) | Fatal adverse outcome^j^ | 0.4 (0.21, 0.75) | .004 |
|  |  |  |  |  |  | BMI 23.0–24.9 (n = NR) |  | 0.29 (0.14, 0.61) | <.001 |
|  |  |  |  |  |  | BMI 25.0–29.9 (n = NR) |  | 0.53 (0.28, 1.03) | .062 |
|  |  |  |  |  |  | BMI ≥30.0 (n = NR) |  | 0.47 (0.17, 1.34) | .157 |

Abbreviations: BMI, body mass index (kg/m^2^); ECMO, extracorporeal membrane oxygenation; ICU, intensive care unit; IMV, invasive mechanical ventilation; MV, mechanical ventilation; NR, not reported.

^a^Paediatric and adult patients. ^b^Residential aged care facility residents. ^c^Hospitalization or death. ^d^ICU admission or death. ^e^Aged 18–39 years. ^f^Aged 40–64 years. ^g^Aged ≥65 years. ^h^Conditional logistic regression. ^i^Cox proportional hazard. ^j^IMV, multiorgan failure, ECMO, or death.

### Supplementary Table 4. Studies assessing hypertension

| **First author** | **Year** | **Country** | **Total** | **Cohort size** | **Reference**  **(sample size)** | **Comparator**  **(sample size)** | **Outcome** | **OR/HR (95%CI)** | ***P*-value** |
| --- | --- | --- | --- | --- | --- | --- | --- | --- | --- |
| Bhatia[1] | 2021 | Australia | 546 | 546 | No hypertension (n = 276) | Hypertension (n = 270) | Mortality | 0.92 (0.48, 1.77) | .812 |
| Davis[2] | 2022 | Australia | 794^a^ | 794 | No hypertension (n = 714) | Hypertension (n = 80) | Hospitalization | 0.88 (0.42, 1.85) | .73 |
| Ellis[3] | 2023 | Australia | 1071^b^ | 1071 | No hypertension (n = 349) | Hypertension (n = 570) | Hospitalization | 1.0 (0.7, 1.5) | NR |
|  |  |  |  |  |  |  | Mortality | 0.9 (0.6, 1.3) | NR |
| Liu[4] | 2021 | Australia | 4054 | 4054 | No hypertension (n = 3541) | Hypertension (n = 513) | Severe COVID-19^c^ | 1.06 (0.85, 1.32) | NR |
|  |  |  |  |  |  |  | Very severe COVID-19^d^ | 0.91 (0.65, 1.27) | NR |
| Yip[101] | 2020 | Hong Kong | 3385 | 1040 | No hypertension (n = 896) | Hypertension (n = 144) | Severe COVID-19 | **2.28 (1.14, 4.54)** | .02 |
| Zhou [13] | 2023 | Hong Kong | 6089 | 6089 | No hypertension (n = 5190) | Hypertension (n = 899) | Severe COVID-19 | **6.76 (5.14, 8.89)** | <.0001 |
| Zhou[14] | 2020 | Hong Kong | 1043 | 1043 | No hypertension (n = 935) | Hypertension (n = 108) | ICU admission | **3.15 (1.25, 5.32)** | .00015 |
| Zhou[15] | 2020 | Hong Kong | 2774 | 2774 | No hypertension (n = 2361) | Hypertension (n = 413) | Mortality | 0.84 (0.36, 1.99) | .856 |
| Fukushima[16] | 2021 | Japan | 234 | 234 | No hypertension (n = 181) | Hypertension (n = 53) | Critical events | **7.15 (2.34, 25.6)** | NR |
| Fukushima[17] | 2023 | Japan | 2430 | 2430 | No hypertension (n = 1567) | Hypertension (n = 863) | Critical events | **1.46 (1.06, 2.00)** | .02 |
| Ito[18] | 2023 | Japan | 936 | 936 | No hypertension (n = 893) | Hypertension (n = 43) | Hospitalization | **3.66 (1.55, 8.69)** | .003 |
| Kurahara[102] | 2021 | Japan | 404 | 404 | No hypertension (n = 228) | Hypertension (n = 176) | Acute respiratory failure requiring oxygenation | 0.89 (0.59, 1.33) | .561 |
| Nishimura[24] | 2023 | Japan | 30 130 | 1097 | No hypertension (n = 305) | Hypertension (n = 178) | Hospitalization | **1.66 (1.07, 2.56)** | .024 |
|  |  |  |  |  | No hypertension (n = 288) | Hypertension (n = 173) | Oxygen administration | 1.01 (0.64, 1.60) | .968 |
| Nojiri[91] | 2023 | Japan | 11 440 | 11 440 | No hypertension (n = 10 877) | Hypertension (n = 563) | Severe COVID-19 | 0.91 (0.70, 1.19) | .5 |
|  |  |  |  | 5980^e^ | No hypertension (n = 5417) | Hypertension (n = 563) | Mortality | 0.98 (0.58, 1.64) | >.9 |
| Sakamoto[28] | 2022 | Japan | 6176 | 6176 | No hypertension (n = 5899) | Hypertension (n = 277) | Hospitalization | 1.27 (0.87, 1.86) | NR |
| Sato[29] | 2022 | Japan | 500 | 500 | No hypertension (n = 332) | Hypertension (n = 168) | Severe COVID-19 | 0.74 (0.63, 2.87) | .45 |
| Takeyama[30] | 2022 | Japan | 2894 | 2894 | No hypertension (n = 2020) | Hypertension (n = 874) | Mortality | 1.28 (0.84, 1.92) | .25 |
| Yamada[32] | 2021 | Japan | 6873 | 1007^f^ | No hypertension (n = NR) | Hypertension (n = NR) | Oxygen therapy | **1.80 (1.34, 2.43)** | <.001 |
| Espiritu[103] | 2023 | Philippine | 10 881 | 10 881 | No hypertension (n = 7234) | Hypertension (n = 3647) | Severe COVID-19 | **1.57 (1.41, 1.75)** | <.001 |
|  |  |  |  |  |  |  | Mortality | **1.33 (1.17, 1.52)** | <.001 |
|  |  |  |  |  |  |  | Respiratory failure | **1.99 (1.75, 2.28)** | <.001 |
|  |  |  |  |  |  |  | ICU admission | **2.16 (1.90, 2.45)** | <.001 |
|  |  |  |  |  |  |  | Mortality | **1.13 (1.01, 1.26)** | .038 |
|  |  |  |  |  |  |  | Respiratory failure | **1.86 (1.65, 2.10)** | <.001 |
|  |  |  |  |  |  |  | ICU admission | **1.99 (1.76, 2.23)** | <.001 |
| Espiritu[35] | 2021 | Philippines | 10 881 | 10 881 | No hypertension (n = 7234) | Hypertension (n = 3647) | Mortality | 0.86 (0.78, 0.95) | .04 |
|  |  |  |  |  |  |  | Respiratory failure | **1.52 (1.36, 1.7)** | .003 |
|  |  |  |  |  |  |  | ICU admission | **1.69 (1.52, 1.89)** | <.001 |
| Tan[39] | 2022 | Singapore | 294 | 294 | No hypertension (n = 269) | Hypertension (n = 25) | ICU admission and/or mortality | 2.85 (0.43, 19.6) | .273 |
|  |  |  |  |  |  |  | Pneumonia | 0.86 (0.06, 9.35) | .907 |
| Byeon[42] | 2021 | South Korea | 12 646 | 12 646 | No diseases of the circulatory system 1 (n = 10 006) | Diseases of the circulatory system 1 (n = 2337) | Mortality | 1.30 (0.96, 1.75) | NR |
|  |  |  |  |  | No diseases of the circulatory system 2 (n = 11 904) | Diseases of the circulatory system 2 (n = 439) | Mortality | 1.18 (0.88, 1.57) | NR |
| Chang[44] | 2020 | South Korea | 211 | 211 | No hypertension (n = 198) | Hypertension (n = 13) | Severe COVID-19 | 2.98 (0.77, 115) | .559 |
| Chang[45] | 2022 | South Korea | 3887^g^ | 3887 | No hypertension (n = 2583) | Hypertension (n = 1304) | Severe COVID-19 | **1.78 (1.33, 2.39)** | <.001 |
| Chang[46] | 2022 | South Korea | 3122^h^ | 3122^h^ | No hypertension (n = 2099) | Hypertension (n = 1023) | Severe COVID-19 | 1.36 (0.96, 1.92) | .084 |
| Cho[47] | 2021 | South Korea | 7590 | 7590 | No hypertension (n = 6127) | Hypertension (n = 1463) | Mortality | **1.89 (1.38, 2.60)** | <.001 |
| Cho[48] | 2021 | South Korea | 5594 | 3729 | No hypertensive (n = 2932) | Hypertensive (n = 795) | Overall survival | 1.21 (0.75, 1.94) | .480 |
| Cho[92] | 2021 | South Korea | 1272^i^ | 1272^i^ | No hypertension (n = 702) | Hypertension (n = 570) | Mortality | **1.96 (1.27, 3.06)** | NR |
|  |  |  |  |  |  |  | Severe COVID-19 | **1.94 (1.29, 2.94)** | NR |
| Chung[50] | 2020 | South Korea | 110 | 110 | No hypertension (n = 73) | Hypertension (n = 37) | Severe COVID-19 | 0.51 (0.13, 2.01) | .333 |
| Huh[52] | 2021 | South Korea | 44 046 | 2805 | No hypertension (n = 1584) | Hypertension (n = 1221) | Severe COVID-19 | 1.18 (0.94, 1.49) | .16 |
| Jang[55] | 2020 | South Korea | 110 | 110 | No hypertension (n = 73) | Hypertension (n = 37) | Severe COVID-19 | 1.00 (0.09, 10.8) | .999 |
| Ji[104] | 2020 | South Korea | 219 961 | 7341 | No isolated hypertension (n = 5713) | Isolated hypertension (n = 1628) | Severe COVID-19 | **1.25 (1.03, 1.51)** | NR |
|  |  |  |  |  | No hypertensive renal disease (n = 7322) | Hypertensive renal disease (n = 19) | Severe COVID-19 | 1.46 (0.48, 4.46) | NR |
| Kang[57] | 2021 | South Korea | 4141 | 3827 | No hypertension (n = 3279) | Hypertension (n = 862) | Severe COVID-19 | 1.64 (1.11, 2.43) | NR |
|  |  |  |  |  |  |  | Mortality | 1.48 (0.96, 2.30) | NR |
| Kang[58] | 2020 | South Korea | 7341 | 7341 | No hypertension | Hypertension (n = 1572) | Mortality | 1.37 (0.99, 1.90) | .057 |
|  |  |  |  |  |  |  | Conventional oxygen therapy | **1.30 (1.09, 1.57)** | .004 |
|  |  |  |  |  |  |  | IMV | **2.04 (1.31, 3.22)** | .002 |
|  |  |  |  |  |  |  | ECMO | **3.17 (1.05, 10.4)** | .047 |
|  |  |  |  |  |  |  | Cardiac arrest | 1.24 (0.60, 2.67) | .566 |
|  |  |  |  |  |  |  | Myocardial infraction | 0.97 (0.68, 1.38) | .884 |
|  |  |  |  |  |  |  | Acute heart failure | 1.28 (0.99, 1.67) | .064 |
|  |  |  |  |  |  |  | Acute kidney injury | **2.67 (1.02, 7.65)** | .054 |
| Kang[105] | 2021 | South Korea | 3788^j^ | 3788 | No hypertension (n = 2598) | Hypertension (n = 1190) | Hospitalization | 1.15 (0.95, 1.38) | .146 |
|  |  |  |  |  |  |  | Oxygen requirement | 1.33 (0.96, 1.83) | .083 |
|  |  |  |  |  |  |  | Severe COVID-19  (severe composite outcome) | **1.67 (1.04, 2.69)** | .034 |
|  |  |  |  |  |  |  | ICU admission | **2.69 (1.33, 5.43)** | .006 |
|  |  |  |  |  |  |  | Ventilator care | **2.85 (1.18, 6.92)** | .02 |
|  |  |  |  |  |  |  | Mortality | 1.24 (0.69, 2.25) | .473 |
| Kim[59] | 2020 | South Korea | 9148 | 9148 | No essential hypertension (n = 7538) | Essential hypertension (n = 1610) | Mortality | 1.32 (0.88, 1.99) | NR |
| Kim[106] | 2022 | South Korea | 129 120 | 8070 | No hypertension (n = NR) | Hypertension (n = 1510) | Severe COVID-19 | **1.63 (1.23, 2.15**) | NR |
| Kim[62] | 2020 | South Korea | 2959 | 2959 | No hypertension (n = 2262) | Hypertension (n = 697) | Severe COVID-19 | 1.26 (0.65, 2.42) | NR |
| Lee[64] | 2020 | South Korea | 5061 | 5061 | No hypertension (n = 4449) | Hypertension (n = 612) | Mortality | 1.24 (0.76, 2.02) | .691 |
| Lee[66] | 2020 | South Korea | 4742 | 4742 | No hypertension (n = 4236) | Hypertension (n = 506) | ICU admission | 1.35 (0.77, 2.38) | NR |
|  |  |  |  | 80^k^ | No hypertension (n = 61) | Hypertension (n = 19) | Ventilator usage | 0.62 (0.12, 3.22) | NR |
| Lee[68] | 2020 | South Korea | 7272 | 7272 | No hypertension (n = 5871) | Hypertension (n = 1401) | Respiratory failure | 1.55 (0.99, 2.43) | .053 |
|  |  |  |  |  |  |  | Mortality | 1.39 (0.99, 1.96) | .053 |
| Lee[70] | 2020 | South Korea | 7339 | 7339 | No hypertension (n = 5966) | Hypertension (n = 1373) | Severe COVID-19 | 1.05 (0.87, 1.27) | .601 |
|  |  |  |  |  |  |  | Mortality | 0.85 (0.61, 1.18) | .323 |
| Oh[76] | 2022 | South Korea | 5077 | 2106^l^ | No hypertension (n = 1633) | Hypertension (n = 473) | Clinical severity | 1.20 (0.79, 1.82) | NR |
|  |  |  |  | 2971^m^ | No hypertension (n = 2308) | Hypertension (n = 663) | Clinical severity | **1.73 (1.08, 2.78)** | .05 |
| Oh[77] | 2021 | South Korea | 122 040 | 7780 | No hypertension (n = NR) | Hypertension (n = NR) | Mortality | 1.36 (0.89, 2.06) | .153 |
| Paek[79] | 2020 | South Korea | 704 | 28 | No hypertension (n = 6) | Hypertension (n = 22) | Mortality | 1.59 (0.45, 5.58) | .47 |
| Shin[84] | 2021 | South Korea | 5571 | 5571 | No hypertension (n = 4375) | Hypertension (n = 1196) | Severe COVID-19 | 1.15 (0.90, 1.47) | .26 |
|  |  |  |  |  |  |  | Mortality | 1.49 (0.95, 2.31) | .08 |
| Song[85] | 2021 | South Korea | 5621 | 5621 | No hypertension (n = 4422) | Hypertension (n = 1199) | Fatal adverse outcome^n,o^ | **1.85 (1.03, 3.35)** | .041 |
|  |  |  |  |  | No hypertension (n = 4422) | Hypertension (n = 1199) | Mortality^n^ | 1.46 (0.93, 2.31) | .102 |
|  |  |  |  |  | No hypertension (n = 4422) | Hypertension (n = 1199) | Fatal adverse outcome^o,p^ | 1.41 (0.94, 2.13) | .096 |
| Wang[86] | 2021 | South Korea | 2800^e^ | 446 | No hypertension (n = 196) | Hypertension (n = 250) | Mortality | 1.13 (0.65, 1.96) | .675 |
| Yun[87] | 2020 | South Korea | 7590 | 7363 | No hypertension (n = 5600) | Hypertension (n = 1763) | Severe COVID-19 | **1.60 (1.14, 2.24)** | <.05 |

Abbreviations: ECMO, extracorporeal membrane oxygenation; ICU, intensive care unit; IMV, invasive mechanical ventilation; NR, not reported.

Diseases of circulatory system 1: Essential (primary) hypertension; Diseases of circulatory system 2: Other hypertensive diseases.

^a^Diseases of circulatory system 1: Essential (primary) hypertension; Diseases of circulatory system 2: Other hypertensive diseases.Paediatric and adult patients. ^b^Residential aged care facility residents. ^c^Hospitalization or death. ^d^ICU or death. ^e^Aged ≥50 years. ^f^Aged ≥65 years. ^g^Aged ≥40 years. ^h^Aged ≥20 years. ^i^Aged ≥60 years. ^j^Aged ≥30 years. ^k^ICU admitted. ^l^Male. ^m^Female. ^n^Multivariable logistic regression. ^o^Cox regression analysis. ^p^IMV, multiorgan failure, ECMO and death.

### Supplementary Table 5. Studies assessing diabetes

| **First author** | **Year** | **Country** | **Total** | **Cohort size** | **Reference**  **(sample size)** | **Comparator**  **(sample size)** | **Outcome** | **OR/HR (95% CI)** | ***P*-value** |
| --- | --- | --- | --- | --- | --- | --- | --- | --- | --- |
| Bhatia[1] | 2021 | Australia | 546 | 546 | No diabetes (n = 403) | Diabetes (n = 143) | Mortality | 1.67 (0.91–3.07) | .097 |
| Davis[2] | 2022 | Australia | 794 | 794 | No diabetes (n = 748) | Diabetes (n = 46) | Hospitalization | 2.10 (0.91–4.81) | .08 |
| Ellis[3] | 2023 | Australia | 1071 | 1071 | No diabetes (n = 659) | Diabetes (n = 256) | Hospitalization | 1.1 (0.7–1.6) | NR |
|  |  |  |  |  |  |  | Mortality | **1.9 (1.3–3.0)** | NR |
| Liu[4] | 2021 | Australia | 4054 | 4054 | No diabetes (n = 3790) | Diabetes (n = 264) | Severe COVID-19^a^ | **1.93 (1.52–2.45)** | NR |
|  |  |  |  |  |  |  | Very severe COVID-19^b^ | **1.88 (1.33–2.67)** | NR |
| Teoh[10] | 2020 | Hong Kong | 2710 | 814 | No diabetes (n = NR) | Diabetes (n = NR) | Severe COVID-19 | **4.77 (2.32–9.84)** | <.001 |
| Wong[11] | 2023 | Hong Kong | 32 222 | 22 604 | No diabetes (n = 17 485) | Diabetes (n = 5119) | Mortality | 0.96 (0.90–1.03) | NR |
| Yip[101] | 2020 | Hong Kong | 3385 | 1040 | No diabetes (n = 959) | Diabetes (n = 81) | Severe COVID-19 | **4.14 (1.96–8.71)** | <.001 |
| Yip[12] | 2021 | Hong Kong | 5639 | 5639 | No diabetes (n = 4460) | Diabetes (n = 1179) | Mortality | **1.94 (1.31–2.88)** | .001 |
| Zhou[13] | 2023 | Hong Kong | 6089 | 6089 | No diabetes (n = 5946) | Diabetes (n = 143) | Severe COVID-19 | **6.14 (4.04–9.33)** | <.0001 |
| Zhou[14] | 2020 | Hong Kong | 1043 | 1043 | No diabetes (n = 989) | Diabetes (n = 54) | ICU admission | **6.17 (2.07–9.36)** | <.0001 |
| Zhou[15] | 2020 | Hong Kong | 2774 | 2774 | No diabetes (n = 2480) | Diabetes (n = 294) | Mortality | 1.81 (0.77–4.36) | .0412 |
| Fukushima[17] | 2023 | Japan | 2430 | 2430 | No diabetes (n = 631) | Prediabetes (n = 993) | Critical events | **2.13 (1.31–3.48)** | .002 |
|  |  |  |  |  |  | Undiagnosed diabetes (n = 244) |  | **4.00 (2.19–7.28)** | <.0001 |
|  |  |  |  |  |  | Diagnosed diabetes (n = 562) |  | **3.96 (2.38–6.58)** | <.0001 |
| Kurahara[102] | 2021 | Japan | 404 | 404 | No diabetes (n = 313) | Diabetes (n = 91) | Acute respiratory failure requiring oxygenation | 1.36 (0.85–2.18) | .204 |
| Miyashita[20] | 2022 | Japan | 937 758 | 937 758 | No diabetes (n = 860 157) | Diabetes (n = 112 102) | Severe COVID-19 | **1.28 (1.25–1.32)** | <.001 |
|  |  |  |  |  |  |  | Mortality | **1.06 (1.02–1.10)** | .002 |
| Muto[21] | 2021 | Japan | 300 | 300 | No diabetes (n = 224) | Diabetes (n = 76) | Critical/severe COVID-19 | 1.87 (0.93–3.73) | .078 |
| Ninomiya[22] | 2021 | Japan | 210 | 210 | No diabetes (n = 187) | Diabetes (n = 23) | Worsened COVID-19/oxygen therapy | **2.85 (1.06–7.7)** | .039 |
| Nishimura[24] | 2023 | Japan | 30 130 | 1097 | No diabetes (n = 396) | Diabetes (n = 87) | Hospitalization | 0.34 (0.19–0.59) | <.001 |
|  |  |  |  |  | No diabetes (n = 365) | Diabetes (n = 96) | Oxygen administration | 0.56 (0.31–0.98) | .044 |
| Nojiri[91] | 2023 | Japan | 11 440 | 11 440 | No diabetes (n = 10 989) | Diabetes (n = 451) | Severe COVID-19 | 1.21 (0.93–1.57) | .2 |
|  |  |  |  | 5980^c^ | No diabetes (NR) | Diabetes (NR) | Mortality | 1.55 (0.92–2.57) | .095 |
| Otoshi[27] | 2021 | Japan | 300 | 300 | No diabetes (n = 232) | Diabetes (n = 68) | Severe COVID-19 | 2.16 (0.98–4.76) | .057 |
| Sakamoto[28] | 2022 | Japan | 6176 | 6176 | No diabetes (n = 6078) | Diabetes (n = 98) | Hospitalization | **1.95 (1.20–3.18)** | NR |
| Sato[29] | 2022 | Japan | 500 | 500 | No diabetes (n = 416) | Diabetes (n = 84) | Mortality or ECMO or IMV or MV or ICU admission | 1.19 (0.54–2.60) | .66 |
| Takeyama[30] | 2022 | Japan | 2894 | 2894 | No diabetes (n = | Diabetes (n = 597) | Mortality | 0.95 (0.61–1.46) | .8 |
| Yamada[32] | 2021 | Japan | 6873 | 1615^d^ | No diabetes (n = NR) | Diabetes (n = NR) | Oxygen therapy | **1.72 (1.21–2.45)** | .003 |
|  |  |  |  | 1007^e^ |  |  |  | **2.02 (1.41–2.89)** | <.001 |
| Jefferies[33] | 2020 | New Zealand | 1503 | 1495 | No diabetes (n = 1438) | Diabetes (n = 57) | Severe COVID-19 | 1.73 (0.82–3.64) | NR |
| Espiritu[107] | 2021 | Philippines | 10 881 | 10 881 | No diabetes (n = 8690) | Diabetes (n = 2191) | Severe COVID-19^f^ | **1.85 (1.65–2.07)** | <.001 |
|  |  |  |  |  |  |  | Mortality^f^ | **1.46 (1.28–1.68)** | <.001 |
|  |  |  |  |  |  |  | Respiratory failure^f^ | **1.67 (1.46–1.90)** | <.001 |
|  |  |  |  |  |  |  | ICU admission^f^ | **1.80 (1.59–2.05)** | <.001 |
|  |  |  |  |  |  |  | Mortality^g^ | **1.19 (1.06–1.33)** | .002 |
|  |  |  |  |  |  |  | Respiratory failure^g^ | **1.51 (1.35–1.69)** | <.001 |
|  |  |  |  |  |  |  | ICU admission^g^ | **1.57 (1.41–1.74)** | <.001 |
| Espiritu[103] | 2021 | Philippines | 10 881 | 10 881 | No diabetes (n = 8690) | Diabetes (n = 2191) | ICU admission | **1.27 (1.14–1.40)** | <.001 |
| Koh[36] | 2021 | Singapore | 1042 | 1042 | Normoglycemic (n = 809) | Diabetes (n = 140) | Severe COVID-19 | **4.24 (12.1–8.55)** | <.001 |
|  |  |  |  |  |  |  | ICU admission | **10.9 (3.54–33.8)** | <.001 |
|  |  |  |  |  |  | Prediabetes (n = 93) | Severe COVID-19 | 0.68 (0.10–2.99) | .611 |
| Tee[40] | 2020 | Singapore | 240 | 201 | No diabetes (n = 200) | Diabetes (n = 19) | Pneumonia | 2.07 (0.42–10.2) | .3783 |
|  |  |  |  |  |  | Prediabetes (n = 21) |  | **10.8 (3.65–32.1)** | <.0001 |
| Bae[41] | 2021 | South Korea | 1760 | 1232 | No diabetes (n = 977) | Diabetes (n = 255) | Mortality | **2.12 (1.46–3.07)** | <.001 |
| Byeon[42] | 2021 | South Korea | 12 646 | 12 646 | No diabetes (n = 11 009) | Diabetes (n = 1334) | Mortality | **1.50 (1.18–1.92)** | NR |
| Chang[43] | 2020 | South Korea | 106 | 106 | No diabetes (n = 64) | Diabetes (n = 42) | Mortality | 1.16 (0.51–2.65) | .724 |
| Chang[44] | 2020 | South Korea | 211 | 211 | No diabetes (n = 207) | Diabetes (n = 4) | Severe COVID-19 | **64.1 (4.59–895)** | .002 |
| Chang[45] | 2022 | South Korea | 3887 | 3887 | No diabetes (n = 3286) | Diabetes (n = 601) | Severe COVID-19 | 1.20 (0.87–1.66) | .261 |
| Chang[46] | 2022 | South Korea | 3122 | 3122 | No diabetes (n = 2659) | Diabetes (n = 463) | Severe COVID-19 | 1.40 (0.98–2.00) | .068 |
| Cho[47] | 2021 | South Korea | 7590 | 7590 | No diabetes (n = 6683) | Diabetes (n = 907) | Mortality | **2.22 (1.63–2.95)** | <.001 |
| Cho[48] | 2021 | South Korea | 5594 | 3729 | No diabetes (n = 3275) | Diabetes (n = 452) | Overall survival | **1.71 (1.08–2.70)** | .610 |
| Cho[92] | 2021 | South Korea | 1272 | 1272 | No diabetes (n = 940) | Diabetes (n = 332) | Mortality | **1.94 (1.26–2.98)** | NR |
|  |  |  |  |  |  |  | Severe COVID-19 | **1.72 (1.15–2.58)** | NR |
| Choi[49] | 2021 | South Korea | 7590 | 7590 | No diabetes (n = 5309) | Diabetes without chronic complications (n = 1775) | Mortality | **1.52 (1.06–2.17)** | .022 |
|  |  |  |  |  |  | Diabetes with chronic complications (n = 506) |  | **1.82 (1.26–2.64)** | .002 |
|  |  |  |  |  |  | Diabetes without chronic complications (n = 1775) | ICU admission | 1.29 (0.92–1.82) | .140 |
|  |  |  |  |  |  | Diabetes with chronic complications (n = 506) |  | **1.81 (1.24–2.64)** | .002 |
| Chung[50] | 2020 | South Korea | 110 | 110 | No diabetes (n = 81) | Diabetes (n = 29) | Severe COVID-19 | **10.8 (3.0–38.7)** | <.001 |
| Her[51] | 2021 | South Korea | 5628 | 3940 | No diabetes (n = 3458) | Diabetes (n = 482) | Mortality | **2.1 (1.33–3.31)** | .001 |
| Huh[52] | 2021 | South Korea | 44 046 | 2805 | No diabetes (n = 1880) | Diabetes (n = 925) | Severe COVID-19 | **1.24 (1.00–1.54)** | .05 |
| Hwang[54] | 2020 | South Korea | 103 | 103 | No diabetes (n = 14) | Diabetes (n = 35) | Mortality | 2.97 (0.94–9.39) | .063 |
| Jang[55] | 2020 | South Korea | 110 | 110 | No diabetes (n = 81) | Diabetes (n = 29) | Severe COVID-19 | **19.2 (1.90–193.4)** | .012 |
| Ji[104] | 2020 | South Korea | 219 961 | 7341 | No diabetes (n = 6298) | Diabetes (n = 1043) | Severe COVID-19 | **1.25 (1.01–1.54)** | NR |
| Kang[57] | 2021 | South Korea | 4141 | 3827 | No diabetes (n = 3640) | Diabetes (n = 501) | Severe COVID-19 | **1.95 (1.32–2.86)** | NR |
|  |  |  |  |  |  |  | Mortality | **2.38 (1.50–3.51)** | NR |
| Kim[59] | 2020 | South Korea | 9148 | 9148 | No diabetes (n = 8239) | Diabetes (n = 909) | Mortality | **1.82 (1.25–2.67)** | NR |
| Kim[60] | 2021 | South Korea | 75 527 | 1911 | No diabetes (n = 1009) | Diabetes (n = 902) | Severity grade 1 | **1.46 (1.17–1.81)** | .0007 |
|  |  |  |  |  |  |  | Severity grade 2 | **1.63 (1.17–2.27)** | .0038 |
|  |  |  |  |  |  |  | Mortality | **1.79 (1.24–2.57)** | .0018 |
| Kim[106] | 2022 | South Korea | 129 120 | 8070 | No diabetes (n = NR) | Diabetes (n = 1724) | Severe COVID-19 | **1.43 (1.09–1.87)** | NR |
| Kim[61] | 2022 | South Korea | 5624 | 5624 | No diabetes (n = 4933) | Diabetes (n = 691) | Mortality | **0.38 (0.21–0.67)** | <.01 |
|  |  |  |  |  |  |  | Clinical severity score | **-0.62 (0.42–0.70)** | <.001 |
| Kim[108] | 2020 | South Korea | 1082 | 1082 | No diabetes (n = 847) | Diabetes (n = 235) | Mortality | **3.53 (2.30–5.41)** | NR |
|  |  |  |  | 470^h^ | No diabetes (n = 235) | Diabetes (n = 235) | Mortality | **2.40 (1.38–4.15)** | NR |
| Kim[62] | 2020 | South Korea | 2959 | 2959 | No diabetes (n = 2528) | Diabetes (n = 431) | Severe COVID-19 | 1.44 (0.72–2.85) | NR |
| Lee[64] | 2020 | South Korea | 5061 | 5061 | No diabetes (n = 4642) | Diabetes (n = 419) | Mortality | 1.52 (0.91–2.55) | .110 |
| Lee[66] | 2020 | South Korea | 4742 | 4742 | No diabetes (n = 4391) | Diabetes (n = 351) | ICU admission | 1.08 (0.58–2.02) | NR |
|  |  |  |  | 80^i^ | No diabetes (n = 66) | Diabetes (n = 14) | Ventilator usage | 1.24 (0.17–9.17) | NR |
| Lee[68] | 2020 | South Korea | 7272 | 7272 | No diabetes (n = 6231) | Diabetes (n = 1041) | Respiratory failure | 1.44 (0.93–2.24) | .099 |
|  |  |  |  |  |  |  | Mortality | **2.02 (1.44–2.84)** | <.001 |
| Lee[70] | 2020 | South Korea | 7339 | 7339 | No diabetes (n = 6482) | Diabetes (n = 857) | Severe COVID-19 | **1.36 (1.11–1.68)** | .003 |
|  |  |  |  |  |  |  | Mortality | **2.17 (1.55–3.03)** | <.001 |
| Lee[71] | 2020 | South Korea | 1005 | 1005 | No diabetes (n = 814) | Diabetes (n = 191) | Severe COVID-19 (pneumonia) | **2.26 (1.43–3.58)** | .001 |
|  |  |  |  |  |  |  | Mortality | **2.29 (1.44–3.64)** | <.001 |
| Moon[74] | 2021 | South Korea | 5626 | 4426 | No diabetes (n = 3920) | Diabetes (n = 506) | Mortality | **3.23 (1.88–5.55)** | <.001 |
|  |  |  |  |  |  |  | 30-day and 60-day survival | **2.00 (1.10–4.69)** | .002 |
| Moon[109] | 2020 | South Korea | 5307 | 5307 | No diabetes (n = 4537) | Diabetes (n = 770) | Hospitalization | 1.07 (0.72–1.59) | .735 |
|  |  |  |  |  |  |  | Oxygen supply | **1.35 (1.1–1.66)** | .004 |
|  |  |  |  |  |  |  | Ventilator support | **1.93 (1.27–2.92)** | <.001 |
|  |  |  |  |  |  |  | Mortality | **2.66 (1.9–3.73)** | <.001 |
| Moon[75] | 2020 | South Korea | 352 | 352 | No diabetes (n = 295) | Diabetes (n = 57) | Mortality | **5.45 (1.54–19.3)** | .001 |
| Oh[76] | 2022 | South Korea | 5077 | 2106^j^ | No diabetes (n = 1794) | Diabetes (n = 312) | Clinical severity | **1.81 (1.18–2.77)** | .01 |
|  |  |  |  | 2971^k^ | No diabetes (n = 2622) | Diabetes (n = 349) |  | **1.87 (1.15–3.02)** | .05 |
| Oh[77] | 2021 | South Korea | 122 040 | 7780 | No diabetes without chronic complication (n = NR) | Diabetes without chronic complication (n = NR) | Mortality | **1.87 (1.35–2.59)** | <.001 |
|  |  |  |  |  | No diabetes with chronic complication (n = NR) | Diabetes with chronic complication (n = NR) | Mortality | **1.61 (1.06–2.45)** | .027 |
| Oh[78] | 2021 | South Korea | 122 040 | 7669 | No diabetes without chronic complication (n = 6091) | Diabetes without chronic complication (n = 1578) | Mortality | **1.74 (1.13–2.69)** | .013 |
|  |  |  |  |  | No diabetes with chronic complication (n = 7366) | Diabetes with chronic complication (n = 303) | Mortality | **1.74 (1.13–2.69)** | .013 |
| Paek[79] | 2020 | South Korea | 704 | 28 | No diabetes (n = 12) | Diabetes (n = 16) | Mortality | 2.47 (0.90–6.78) | .079 |
| Park[81] | 2020 | South Korea | 1005 | 289 | No diabetes on admission (n = 196) | Diabetes on admission (n = 93) | Mortality | **1.92 (1.18–3.11)** | .009 |
| Seong[83] | 2021 | South Korea | 488 | 318 | No diabetes (n = 202) | Diabetes (n = 116) | Mortality | **1.62 (1.03–2.56)** | .037 |
| Shin[84] | 2021 | South Korea | 5571 | 5571 | No diabetes (n = 4885) | Diabetes (n = 686) | Severe COVID-19 | 1.08 (0.82–1.42) | .6 |
|  |  |  |  |  |  |  | Mortality | **2.26 (1.46–3.49)** | <.01 |
| Song[85] | 2021 | South Korea | 5621 | 5621 | No diabetes (n = 4932) | Diabetes (n = 689) | Mortality^f^ | **2.73 (1.44–5.22)** | .002 |
|  |  |  |  |  |  |  | Fatal adverse outcome^fl^ | **1.84 (1.0–3.37)** | .049 |
|  |  |  |  |  |  |  | Mortality^g,l^ | 1.44 (0.92–2.26) | .114 |
|  |  |  |  |  |  |  | Fatal adverse outcome^g,l^ | 1.17 (0.77–1.77) | .467 |
| Wang[86] | 2021 | South Korea | 2800 | 446 | No diabetes (n = 314) | Diabetes (n = 132) | Mortality | **2.32 (1.32–4.10)** | .004 |
| Yun[87] | 2020 | South Korea | 7590 | 7363 | No diabetes (n = 5816) | Diabetes (n = 1547) | Severe COVID-19 | **1.55 (1.55–2.06)** | <.05 |
| Do[90] | 2023 | Vietnam | 504 | 504 | No diabetes (n = 378) | Diabetes (n = 126) | Mortality | **2.17 (1.27–3.71)** | .004 |

Abbreviations: ECMO, extracorporeal membrane oxygenation; HFNC, high-flow nasal cannula; HR, hazard ratio; ICU, intensive care unit; IMV, invasive mechanical ventilation; MV, mechanical ventilation; NR, not reported; OR, odds ratio.

^a^Hospitalization or death. ^b^ICU admission or death. ^c^Aged ≥50 years. ^d^Aged 40–64 years. ^e^Aged ≥65 years. ^f^Conditional logistic regression. ^g^Cox proportional hazard. ^h^Propensity score matched subgroup. ^i^ICU-admitted cohort. ^j^Male. ^k^Female. ^l^IMV multiorgan failure, ECMO and death.

### Supplementary Table 6. Studies assessing chronic respiratory disease

| **First author** | **Year** | **Country** | **Total** | **Cohort size** | **Reference**  **(sample size)** | **Comparator**  **(sample size)** | **Outcome** | **OR/HR (95% CI)** | ***P*-value** |
| --- | --- | --- | --- | --- | --- | --- | --- | --- | --- |
| Bhatia[1] | 2021 | Australia | 546 | 546 | No COPD (n = 500) | COPD (n = 46) | Mortality | **2.27 (1.06–4.85)** | .035 |
| Ellis[3] | 2023 | Australia | 1071^a^ | 1071 | No chronic lung disease (n = 734) | Chronic lung disease (n = 180) | Hospitalization | 1.4 (0.9–2.1) | NR |
|  |  |  |  |  | No asthma (n = 841) | Asthma (n = 72) |  | **2.2 (1.2–3.8)** | NR |
|  |  |  |  |  | No chronic lung disease (n = 734) | Chronic lung disease (n = 180) | Mortality | **1.7 (1.1–2.7)** | NR |
|  |  |  |  |  | No asthma (n = 841) | Asthma (n = 72) |  | 0.5 (0.2–1.3) | NR |
| Liu[4] | 2021 | Australia | 4054 | 4054 | No COPD (n = 3761) | COPD (n = 293) | Severe COVID-19^b^ | **1.81 (1.43–2.29)** | NR |
|  |  |  |  |  | No asthma (n = 3638) | Asthma (n = 416) |  | 0.91 (0.68–1.22) | NR |
|  |  |  |  |  | No COPD (n = 3761) | COPD (n = 293) | Very severe COVID-19^c^ | **1.68 (1.18–2.38)** | NR |
|  |  |  |  |  | No asthma (n = 3638) | Asthma (n = 416) |  | 0.86 (0.53–1.41) | NR |
| Wang[7] | 2023 | Australia | 1082 | 1082 | No COPD (n = 1010) | COPD (n = 72) | Oxygen requirement | **4.19 (1.60–11)** | .003 |
| Zhou[13] | 2023 | Hong Kong | 6089 | 6089 | No COPD (n = 6008) | COPD (n = 81) | Severe COVID-19 | 0.88 (0.22–3.54) | .857 |
| Zhou[14] | 2020 | Hong Kong | 1043 | 1043 | No respiratory disease (n = 813) | Respiratory disease (n = 230) | ICU admission | **8.15 (1.85–14.4)** | <.0001 |
| Kurahara[102] | 2021 | Japan | 404 | 404 | No underlying pulmonary condition (n = 318) | Underlying pulmonary condition (n = 86) | Acute respiratory failure requiring oxygenation | 1.23 (0.76–2.01) | .402 |
| Miyashita[20] | 2022 | Japan | 937 758 | 937 758 | No asthma (n = 856 205) | Asthma (n = 81 553) | Severe COVID-19 | **1.04 (1.01–1.08)** | .03 |
|  |  |  |  |  | No COPD (n = 930 485) | COPD (n = 7273) |  | **1.19 (1.11–1.27)** | <.001 |
|  |  |  |  |  | No (other) chronic pulmonary disease (n = 881 819) | Other chronic pulmonary disease (n = 55 939) |  | **1.15 (1.11–1.19)** | <.001 |
|  |  |  |  |  | No asthma (n = 817 831) | Asthma (n = 81 553) | Mortality | 0.92 (0.87–0.96) | <.001 |
|  |  |  |  |  | No COPD (n = 856 205) | COPD (n = 7273) |  | **1.21 (1.12–1.31)** | <.001 |
|  |  |  |  |  | No (other) chronic pulmonary disease (n = 930 485) | Other chronic pulmonary disease (n = 55 939) |  | **1.16 (1.11–1.22)** | <.001 |
| Nishimura[24] | 2023 | Japan | 30 130 | 1097 | No COPD (n = 381) | COPD (n = 102) | Hospitalization | 0.39 (0.17–0.89) | .024 |
|  |  |  |  |  | No asthma (n = 366) | Asthma (n = 117) |  | 0.86 (0.46–1.62) | .648 |
|  |  |  |  |  | No COPD (n = 348) | COPD (n = 113) | Oxygen administration | 1.75 (0.78–3.93) | .178 |
|  |  |  |  |  | No asthma (n = 339) | Asthma (n = 122) |  | 0.75 (0.39–1.47) | .407 |
| Nojiri[91] | 2023 | Japan | 11 440 | 11 440 | No COPD (n = 11 398) | COPD (n = 42) | Severe COVID-19 | 1.78 (0.93–3.47) | .085 |
|  |  |  |  |  | No asthma (n = 11 300) | Asthma (n = 140) | Severe COVID-19 | 1.07 (0.74–1.53) | .7 |
|  |  |  |  | 5980^d^ | No COPD (n = 5938) | COPD (n = 42) | Mortality | 2.18 (0.78–5.47) | .11 |
|  |  |  |  |  | No asthma (n = 5840) | Asthma (n = 140) | Mortality | 0.9 (0.37–1.95) | .8 |
| Sakamoto[28] | 2022 | Japan | 6176 | 6176 | No COPD (n = 5854) | COPD (n = 322) | Hospitalization | 1.27 (0.78–2.07) | NR |
| Sato[29] | 2022 | Japan | 500 | 500 | No COPD (n = 472) | COPD (n = 28) | Mortality or ECMO or IMV or MV or ICU admission | 0.97 (0.29–3.26) | .96 |
| Takeyama[30] | 2022 | Japan | 2894 | 2894 | No respiratory disease (n = | Respiratory disease (n = 298) | Mortality | 0.76 (0.421.38) | .37 |
| Jefferies[33] | 2020 | New Zealand | 1503 | 1495 | No chronic lung condition (n = 1366) | Chronic lung condition (n = 129) | Severe COVID-19 | 1.12 (0.56–2.23) | NR |
| Chang[43] | 2020 | South Korea | 106 | 106 | No chronic lung disease (n = 99) | Chronic lung disease (n = 7) | Mortality | 1.69 (0.63–4.56) | .302 |
| Chang[44] | 2022 | South Korea | 3887 | 3887 | No asthma (n = 3675) | Asthma (n = 212) | Severe COVID-19 | 1.33 (0.84–2.12) | .222 |
| Chang[46] | 2022 | South Korea | 3122 | 3122 | No asthma (n = 2942) | Asthma (n = 180) | Severe COVID-19 | 1.14 (0.68–1.90) | .627 |
| Cho[47] | 2021 | South Korea | 7590 | 7590 | No chronic pulmonary disease (n = 6632) | Chronic pulmonary disease (n = 958) | Mortality | **1.88 (1.38–2.58)** | <.001 |
| Cho[48] | 2021 | South Korea | 5594 | 3729 | No chronic pulmonary disease (n = 3628) | Chronic pulmonary disease (n = 101) | Overall survival | 1.76 (0.86–3.63) | .250 |
| Cho[92] | 2021 | South Korea | 1272 | 1272 | No lung disease (n = 1211) | Lung disease (n = 61) | Mortality | 2.13 (0.96–4.41) | NR |
|  |  |  |  |  |  |  | Severe COVID-19 | 1.91 (0.89–3.86) | NR |
| Choi[49] | 2021 | South Korea | 7590 | 7590 | No chronic pulmonary disease (n = 4202) | Chronic pulmonary disease (n = 3388) | Mortality | 1.21 (0.87–1.68) | .264 |
|  |  |  |  |  |  |  | ICU admission | 1.09 (0.81–1.47) | .585 |
| Chung[50] | 2020 | South Korea | 110 | 110 | No chronic lung disease (n = 106) | Chronic lung disease (n = 4) | Severe COVID-19 | 2.06 (0.15–28.8) | .592 |
| Huh[52] | 2021 | South Korea | 44 046 | 2805 | No chronic lung disease (n = 2036) | Chronic lung disease (n = 769) | Severe COVID-19 | 1.21 (0.99–1.47) | .06 |
|  |  |  |  |  | No asthma and allergic rhinitis (n = 1122) | Asthma and allergic rhinitis (n = 1683) |  | 0.92 (0.76–1.11) | .4 |
| Hwang[54] | 2020 | South Korea | 103 | 103 | No chronic lung disease (n = 96) | Chronic lung disease (n = 7) | Mortality | **13.7 (2.08–90)** | .007 |
| Ji[104] | 2020 | South Korea | 219 961 | 7341 | No chronic upper respiratory disease (n = 2911) | Chronic upper respiratory disease (n = 4430) | Severe COVID-19 | 0.90 (0.74–1.09) | NR |
|  |  |  |  |  | No chronic lower respiratory disease (n = 5702) | Chronic lower respiratory disease (n = 1639) |  | 1.22 (1.00–1.47) | NR |
|  |  |  |  |  | No environmental lung disease (n = 7330) | Environmental lung disease (n = 11) |  | 2.64 (0.54–12.8) | NR |
|  |  |  |  |  | No interstitial lung disease (n = 7329) | Interstitial lung disease (n = 12) |  | 5.33 (0.98–29.1) | NR |
|  |  |  |  |  | No pulmonary vascular disease (n = 7330) | Pulmonary vascular disease (n = 11) |  | 0.58 (0.13–2.62) | NR |
| Jung[110] | 2021 | South Korea | 20 330 | 4066 | No asthma (n = 3704) | Severe asthma (n = 40) | Severe COVID-19 | 0.50 (0.14–1.80) | .288 |
|  |  |  |  |  | No COPD (n = 3902) | Mild COPD (n = 101) |  | 0.99 (0.51–1.95) | .980 |
|  |  |  |  |  |  | Severe COPD (n = 63) |  | **2.23 (1.08–4.6)** | .030 |
|  |  |  |  |  | No asthma (n = 3704) | Mild asthma (n = 322) | Mortality | 0.85 (0.45–1.60) | .605 |
|  |  |  |  |  |  | Severe asthma (n = 40) |  | 0.70 (0.13–3.68) | .672 |
|  |  |  |  |  | No COPD (n = 3902) | Mild COPD (n = 101) |  | 1.10 (0.42–2.89) | .851 |
|  |  |  |  |  |  | Severe COPD (n = 63) |  | **3.06 (1.14–8.20)** | .026 |
| Kim[60] | 2021 | South Korea | 75 527 | 1911 | No COPD (n = 46) | COPD (n = 1865) | Severity grade 1 | 1.44 (0.76–2.69) | .2611 |
|  |  |  |  |  |  |  | Severity grade 2 | 0.92 (0.41–2.05) | .8334 |
|  |  |  |  |  |  |  | Mortality | 1.04 (0.46–2.34) | .9345 |
| Kim[106] | 2022 | South Korea | 129 120 | 8070 | No pulmonary disease (n = NR) | Pulmonary disease (n = 3338) | Severe COVID-19 | **1.72 (1.35–2.19)** | NR |
| Kim[62] | 2020 | South Korea | 2959 | 2959 | No COPD (n = 2931) | COPD (n = 28) | Severe COVID-19 | 1.11 (0.18–6.77) | NR |
|  |  |  |  |  | No asthma (n = 2879) | Asthma (n = 80) |  | 0.70 (0.14–3.54) | NR |
| Lee[64] | 2020 | South Korea | 5061 | 5061 | No pulmonary disease (n = 4632) | Pulmonary disease (n = 429) | Mortality | 0.59 (0.27–1.3) | .187 |
| Lee[66] | 2020 | South Korea | 4742 | 4742 | No COPD (n = 4342) | COPD (n = 400) | ICU admission | 0.74 (0.33–1.65) | NR |
|  |  |  |  | 80^e^ | No COPD (n = 73) | COPD (n = 7) | Ventilator usage | 0.44 (0.02–9.62) | NR |
| Lee[67] | 2023 | South Korea | 584 | 584 | No COPD (n = 569) | COPD (n = 15) | Mortality | 3.45 (0.5–23.9) | .209 |
| Lee[68] | 2020 | South Korea | 7272 | 7272 | No asthma (n = 6586) | Asthma (n = 686) | Respiratory failure | 0.99 (0.58–1.70) | .997 |
|  |  |  |  | 686 | Mild asthma (n = 614) | Moderate to severe asthma (n = 72) |  | 0.66 (0.15–2.82) | .581 |
|  |  |  |  | 7272 | No asthma (n = 6586) | Asthma (n = 686) | Mortality | 1.06 (0.71–1.59) | .759 |
|  |  |  |  | 686 | Mild asthma (n = 614) | Moderate to severe asthma (n = 72) |  | 1.33 (0.54–3.30) | .526 |
| Lee[69] | 2020 | South Korea | 4610 | 4610 | No COPD (n = 4469) | COPD (n = 141) | Respiratory failure | 1.03 (0.48–2.25) | NR |
|  |  |  |  |  |  |  | Mortality | **1.80 (1.11–2.93)** | NR |
| Lee[70] | 2020 | South Korea | 7339 | 7339 | No COPD (n = 7258) | COPD (n = 81) | Severe COVID-19 | 1.02 (0.57–1.82) | .96 |
|  |  |  |  |  | No asthma (n = 6972) | Asthma (n = 367) | Severe COVID-19 | 1.22 (0.89–1.68) | .223 |
|  |  |  |  |  | No COPD (n = 7258) | COPD (n = 81) | Mortality | 1.39 (0.65–2.97) | .397 |
|  |  |  |  |  | No asthma (n = 6972) | Asthma (n = 367) | Mortality | 0.68 (0.38–1.21) | .19 |
| Lee[71] | 2020 | South Korea | 1005 | 1005 | No COPD (n = 990) | COPD (n = 14) | Mortality | **4.52 (1.85–11.0)** | .001 |
| Lim[73] | 2021 | South Korea | 146 | 146 | No COPD (n = 138) | COPD (n = 8) | Mortality | **8.07 (1.2–54.5)** | .032 |
| Oh[76] | 2022 | South Korea | 5077 | 2106^f^ | No asthma (n = 2060) | Asthma (n = 46) | Clinical severity | 0.68 (0.18–2.64) | NR |
|  |  |  |  |  | No COPD (n = 2082) | COPD (n = 24) |  | 2.55 (0.84–7.78) | NR |
|  |  |  |  | 2971^g^ | No asthma (n = 2896) | Asthma (n = 75) |  | 1.78 (0.70–4.48) | NR |
|  |  |  |  |  | No COPD (n = 2,956) | COPD (n = 15) |  | 1.27 (0.29–5.59) | NR |
| Oh[77] | 2021 | South Korea | 122 040 | 7780 | No chronic respiratory diseases (n = NR) | Chronic respiratory diseases (n = NR) | Mortality | 1.19 (0.86–1.64) | .299 |
|  |  |  |  |  | No COPD (n = NR) | COPD (n = NR) |  | **1.56 (1.06–2.2)** | .024 |
|  |  |  |  |  | No asthma (n = NR) | Asthma (n = NR) |  | 1.03 (0.76–1.41) | .834 |
|  |  |  |  |  | No interstitial lung disease (n = NR) | Interstitial lung disease (n = NR) |  | 1.83 (0.74–4.55) | .193 |
|  |  |  |  |  | No lung disease due to external agent (n = NR) | Lung disease due to external agent (n = NR) |  | **3.54 (1.70–7.38)** | <.001 |
|  |  |  |  |  | No obstructive sleep apnea (n = NR) | Obstructive sleep apnea (n = NR) |  | 0.47 (0.06–3.94) | .486 |
| Oh[78] | 2021 | South Korea | 122 040 | 7669 | No chronic pulmonary disease (n = 5215) | Chronic pulmonary disease (n = 2454) | Mortality | **1.69 (1.23–2.33)** | .001 |
| Park[80] | 2020 | Korea | 2269 | 2269 | No bronchial asthma (n = = 2202) | Bronchial asthma (n = 67) | Mortality | 2.13 (0.74–6.13) | .158 |
|  |  |  |  |  | No COPD (n = 2,238) | COPD (n = 31) |  | 1.05 (0.26–4.30) | .937 |
| Seong[83] | 2021 | South Korea | 488 | 318 | No COPD (n = 306) | COPD (n = 12) | Mortality | **4.2 (1.87–9.84)** | .001 |
| Shin[84] | 2021 | South Korea | 5571 | 5571 | No asthma (n = 5443) | Asthma (n = 128) | Severe COVID-19^h^ | 1.13 (0.60–2.12) | .71 |
|  |  |  |  |  | No COPD (n = 5531) | COPD (n = 40) | Severe COVID-19^i^ | **3.19 (1.35–7.52)** | <.01 |
|  |  |  |  |  | No asthma (n = 5443) | Asthma (n = 128) | Mortality^h^ | 2.20 (0.86–5.59) | .1 |
|  |  |  |  |  | No COPD (n = 5531) | COPD (n = 40) | Mortality^i^ | 1.39 (0.35–5.59) | .64 |
| Song[85] | 2021 | South Korea | 5621 | 5621 | No COPD (n = 5581) | COPD (n = 40) | Mortality^h^ | 3.50 (0.63–1.62) | .126 |
|  |  |  |  |  | No COPD (n = 5,581) | COPD (n = 40) | Fatal adverse outcome^h,j^ | 4.77 (0.98–19.6) | .039 |
|  |  |  |  |  | No asthma (n = 5493) | Asthma (n = 128) | Mortality^h^ | 1.35 (0.54–3.33) | .521 |
|  |  |  |  |  | No COPD (n = 5581) | COPD (n = 40) | Mortality^h^ | 1.46 (0.49–4.37) | .501 |
|  |  |  |  |  | No asthma (n = 5493) | Asthma (n = 128) | Fatal adverse outcome^i,j^ | 1.01 (0.41–2.44) | .99 |
|  |  |  |  |  | No COPD (n = 5581) | COPD (n = 40) | Fatal adverse outcome^i,j^ | 1.35 (0.49–3.73) | .566 |
| Yang[111] | 2021 | South Korea | 7340 | 7340 | No asthma (n = 537) | Asthma (n = 537) | Severe COVID-19 | **1.62 (1.01–2.67)** | NR |
|  |  |  |  |  | No allergic asthma (n = 515) | Allergic asthma (n = 515) |  | 1.40 (0.83–2.41) | NR |
|  |  |  |  |  | No nonallergic asthma (n = 290) | Nonallergic asthma (n = 290) |  | **4.09 (1.69–10.5)** | NR |
| Yun[87] | 2020 | South Korea | 7590 | 7363 | No COPD (n = 5542) | COPD (n = 1821) | Severe COVID-19 | 1.13 (0.85–1.50) | .39 |

Abbreviations: COPD, chronic obstructive pulmonary disease; ECMO, extracorporeal membrane oxygenation; HFNC, high-flow nasal cannula; HR, hazard ratio; ICU, intensive care unit; IMV, invasive mechanical ventilation; MV, mechanical ventilation; NR, not reported; OR, adds ratio.

^a^Residential aged care facility residents. ^b^Hospitalization or death. ^c^ICU admission or death. ^d^Aged ≥50 years. ^e^ICU-admitted population. ^f^Male. ^g^Female. ^h^Conditional logistic regression. ^i^Cox proportional hazard. ^j^IMV, multiorgan failure, ECMO, and death.

### Supplementary Table 7. Studies assessing cardiovascular conditions

| **First author** | **Year** | **Country** | **Total** | **Cohort size** | **Reference Group**  **(sample size)** | **Condition**  **(sample size)** | **Outcome** | **OR/HR (95% CI)** | ***P*-value** |
| --- | --- | --- | --- | --- | --- | --- | --- | --- | --- |
| Bhatia[1] | 2021 | Australia | 546 | 546 | No HF or cardiomyopathy (n = 511) | HF or cardiomyopathy (n = 35) | Mortality | **2.71 (1.13–6.53)** | .026 |
|  |  |  |  |  | No severe valvular disease (n = 533) | Severe valvular disease (n = 13) |  | 1.95 (0.51–7.44) | .33 |
|  |  |  |  |  | No CAD (n = 482) | CAD (n = 64) |  | 1.22 (0.58–2.56) | .604 |
|  |  |  |  |  | No AF or flutter (n = 492) | AF or flutter (n = 54) |  | 0.73 (0.33–1.61) | .435 |
| Davis[2] | 2022 | Australia | 794^a^ | 794 | No cardiovascular comorbidities (n = 778) | Cardiovascular comorbidities (n = 16) | Hospitalization | 1.47 (0.37–5.89) | .58 |
| Ellis[3] | 2023 | Australia | 1071^b^ | 1071 | No IHD (n = 663) | IHD (n = 256) | Hospitalization | 1.3 (0.9–1.9) | NR |
|  |  |  |  |  | No AF (n = 713) | AF (n = 198) |  | 1.1 (0.8–1.8) | NR |
|  |  |  |  |  | No HF (n = 770) | HF (n = 143) |  | **1.7 (1.1–2.7)** | NR |
|  |  |  |  |  | No IHD (n = 663) | IHD (n = 256) | Mortality | 0.9 (0.6–1.5) | NR |
|  |  |  |  |  | No AF (n = 713) | AF (n = 198) |  | 1.2 (0.7–1.9) | NR |
|  |  |  |  |  | No HF (n = 770) | HF (n = 143) |  | **2.0 (1.2–3.3)** | NR |
| Liu[4] | 2021 | Australia | 4054 | 4054 | No IHD (n = 3940) | IHD (n = 114) | Severe COVID-19^c^ | **1.52 (1.1–2.1)** | NR |
|  |  |  |  |  |  |  | Very severe COVID-19^d^ | 1.17 (0.70–1.94) | NR |
| Wang[7] | 2023 | Australia | 1082 | 1082 | No IHD (n = 918) | IHD (n = 164) | Oxygen requirement | **1.91 (1.05–3.49)** | .034 |
| Wong[11] | 2023 | Hong Kong | 32 222 | 22 604 | No CVDs (n = 12 617) | CVDs (n = 9987) | Mortality | **1.47 (1.39–1.56)** | NR |
| Zhou[13] | 2023 | Hong Kong | 6089 | 6089 | No HF (n = 6057) | HF (n = 32) | Severe COVID-19 | **4.68 (1.74–12.6)** | .0023 |
|  |  |  |  |  | No AF (n = 6000) | AF (n = 89) |  | **5.81 (3.37–10.0)** | <.0001 |
|  |  |  |  |  | No ventricular tachycardia/fibrillation (n = 6058) | Ventricular tachycardia/fibrillation (n = 31) |  | **14.3 (7.30–27.9)** | <.0001 |
|  |  |  |  |  | No acute MI (n = 6015) | Acute MI (n = 74) |  | **9.16 (5.57–15.06)** | <.0001 |
|  |  |  |  |  | No IHD (n = 5893) | IHD (n = 196) |  | **6.67 (4.59–9.68)** | <.0001 |
|  |  |  |  |  | No peripheral vascular disease (n = 6062) | Peripheral vascular disease (n = 27) |  | **6.73 (2.50–18.1)** | .0002 |
| Zhou[14] | 2020 | Hong Kong | 1043 | 1043 | No CVD (n = 1013) | CVD (n = 10) | ICU admission | 3.12 (0.81–10.1) | <.0001 |
| Zhou[15] | 2020 | Hong Kong | 2774 | 2774 | No CVD (n = 2578) | CVD (n = 196) | Mortality | 1.12 (0.47–2.66) | <.0001 |
| Fukushima[17] | 2023 | Japan | 2430 | 2430 | No CVD (n = 2177) | CVD (n = 253) | Critical events | 1.29 (0.83–2.00) | .25 |
| Kurahara[102] | 2021 | Japan | 404 | 404 | No CVD (n = 374) | CVD (n = 30) | Acute respiratory failure requiring oxygenation | **3.72 (1.55–8.96)** | .003 |
| Miyashita[20] | 2022 | Japan | 937 758 | 937 758 | No MI (n = 928 742) | MI (n = 9016) | Severe COVID-19 | 1.01 (0.95–1.08) | .7 |
|  |  |  |  |  | No congestive HF (n = 879 983) | Congestive HF (n = 57 775) |  | **1.28 (1.24–1.33)** | <.001 |
|  |  |  |  |  | No peripheral vascular disease (n = 903 990) | Peripheral vascular disease (n = 33 768) |  | 1.02 (0.98–1.06) | .28 |
|  |  |  |  |  | No MI (n = 928 742) | MI (n = 9016) | Mortality | 0.99 (0.92–1.07) | .78 |
|  |  |  |  |  | No congestive HF (n = 879 983) | Congestive HF (n = 57 775) |  | **1.36 (1.31–1.42)** | <.001 |
|  |  |  |  |  | No peripheral vascular disease (n = 903 990) | Peripheral vascular disease (n = 33 768) |  | 0.99 (0.95–1.04) | .76 |
| Nishimura[24] | 2023 | Japan | 30 130 | 1097 | No CVD (n = 381) | CVD (n = 102) | Hospitalization | **2.38 (1.16–4.85)** | .017 |
|  |  |  |  |  | No CVD (n = 355) | CVD (n = 106) | Oxygen administration | 1.71 (0.83–3.54) | .147 |
| Nojiri[91] | 2023 | Japan | 11 440 | 11 440 | No MI (n = 11 195) | MI (n = 245) | Severe COVID-19 | 0.78 (0.55–1.10) | .2 |
|  |  |  |  |  | No cardiac arrhythmia (n = 11 245) | Cardiac arrhythmia (n = 195) |  | 0.78 (0.55–1.10) | 1.2 |
|  |  |  |  |  | No peripheral artery disease (n = 11 243) | Peripheral artery disease (n = 197) |  | 0.97 (0.68–1.36) | .9 |
|  |  |  |  | 5,980^e^ | No MI (n = NR) | MI (n = NR) | Mortality | 0.64 (0.32–1.24) | .2 |
|  |  |  |  |  | No cardiac arrhythmia (n = NR) | Cardiac arrhythmia (n = 195) |  | 0.97 (0.5–1.81) | >.9 |
|  |  |  |  |  | No peripheral artery disease (n = NR) | Peripheral artery disease (n = NR) |  | 1.13 (0.58–2.08) | .7 |
| Sakamoto[28] | 2022 | Japan | 6176 | 6176 | No heart disease (n = 6117) | Heart disease (n = 59) | Hospitalization | 0.74 (0.29–1.88) | NR |
| Takeyama[30] | 2022 | Japan | 2894 | 2894 | No heart disease (n = 2639) | Heart disease (n = 255) | Mortality | **1.69 (1.03–2.76)** | .04 |
| Yamada[32] | 2021 | Japan | 6873 | 1007 | No congestive HF (n = NR) | Congestive HF (n = NR) | Oxygen therapy | **2.13 (1.04–4.36)** | .039 |
| Jefferies[33] | 2020 | New Zealand | 1503 | 1495 | No CVD (including hypertension) (n = 1383) | CVD (including hypertension) (n = 112) | Severe COVID-19 | 1.28 (0.71–2.31) | NR |
| Bae[41] | 2021 | South Korea | 1760 | 1232 | No HF (n = 1206) | HF (n = 26) | Mortality | 1.81 (0.94–3.51) | .077 |
| Byeon[42] | 2021 | South Korea | 12 646 | 12 646 | No diseases of the circulatory system 3 (n = 11 516) | Diseases of the circulatory system 3 (n = 827) | Mortality | **1.31 (1.02–1.69)** | NR |
|  |  |  |  |  | No diseases of the circulatory system 5 (n = 11 637) | Diseases of the circulatory system 5 (n = 706) |  | 1.11 (0.85–1.44) | NR |
| Chang[45] | 2022 | South Korea | 3887 | 3887 | No CAD (n = 3678) | CAD (n = 209) | Severe COVID-19 | 1.21 (0.79–1.85) | .386 |
| Chang[46] | 2022 | South Korea | 3122 | 3122 | No CAD (n = 2961) | CAD (n = 161) | Severe COVID-19 | 1.03 (0.62–1.71) | .926 |
|  |  |  |  |  | No AF (n = 3061) | AF (n = 61) |  | **2.21 (1.16–4.21)** | .016 |
|  |  |  |  |  | No HF (n = 2963) | HF (n = 159) |  | **1.85 (1.15–2.96)** | .011 |
| Cho[47] | 2021 | South Korea | 7590 | 7590 | No chronic HF (n = 7378) | Chronic HF (n = 212) | Mortality | **2.14 (1.42–3.23)** | <.001 |
|  |  |  |  |  | No MI (n = NR) | MI (n = NR) |  | 2.25 (0.99–5.10) | .052 |
|  |  |  |  |  | No peripheral vascular disease (n = NR) | Peripheral vascular disease (n = NR) |  | 1.34 (0.91–1.98) | .144 |
|  |  |  |  |  | No CVD (n = NR) | CVD (n = NR) |  | 1.21 (0.84–1.74) | .301 |
| Cho[48] | 2021 | South Korea | 5594 | 3729 | No chronic cardiac disease (n = 3582) | Chronic cardiac disease (n = 147) | Overall survival | 1.15 (0.61–2.15) | .570 |
| Cho[92] | 2021 | South Korea | 1272 | 1272 | No heart disease (n = 1145) | Heart disease (n = 127) | Mortality | 1.69 (0.94–2.92) | NR |
|  |  |  |  |  | No heart disease (n = 1145) | Heart disease (n = 127) | Severe COVID-19 | **1.78 (1.04–2.96)** | NR |
| Choi[49] | 2021 | South Korea | 7590 | 7590 | No MI (n = 7391) | MI (n = 199) | Mortality | 0.62 (0.34–1.12) | .106 |
|  |  |  |  |  | No congestive HF (n = 7083) | Congestive HF (n = 507) |  | **1.72 (1.21–2.46)** | .003 |
|  |  |  |  |  | No peripheral vascular disease (n = 6526) | Peripheral vascular disease (n = 1064) |  | 1.06 (0.76–1.48) | .721 |
|  |  |  |  |  | No MI (n = 7391) | MI (n = 199) | ICU admission | 0.7 (0.36–1.34) | .275 |
|  |  |  |  |  | No congestive HF (n = 7083) | Congestive HF (n = 507) |  | 1.21 (0.81–1.8) | .347 |
|  |  |  |  |  | No peripheral vascular disease (n = 6526) | Peripheral vascular disease (n = 1064) |  | 1.1 (0.79–1.54) | .587 |
| Huh[52] | 2021 | South Korea | 44 046 | 2805 | No chronic heart disease (n = 2342) | Chronic heart disease (n = 463) | Severe COVID-19 | **1.31 (1.04–1.65)** | .02 |
| Hwang[54] | 2020 | South Korea | 103 | 103 | No CVD (n = 91) | CVD (n = 12) | Mortality | 2.56 (0.54–12.2) | .239 |
| Ji[104] | 2020 | South Korea | 219 961 | 7341 | No IHD (n = 7035) | IHD (n = 306) | Severe COVID-19 | 0.81 (0.6–1.11) | NR |
|  |  |  |  |  | No HF and cardiomyopathy (n = 7075) | HF and cardiomyopathy (n = 266) |  | 1.3 (0.93–1.82) | NR |
|  |  |  |  |  | No cardiac arrhythmia (n = 7140) | Cardiac arrhythmia (n = 201) |  | 1.26 (0.87–1.82) | NR |
| Kim[59] | 2020 | South Korea | 9148 | 9148 | No acute MI (n = 9085) | Acute MI (n = 63) | Mortality | **2.38 (1.03–5.49)** | NR |
|  |  |  |  |  | No other IHDs (n = 8778) | Other IHDs (n = 370) |  | **1.71 (1.09–2.66)** | NR |
|  |  |  |  |  | No HF (n = 8999) | HF (n = 149) |  | **3.17 (1.88–5.34)** | NR |
| Kim[60] | 2021 | South Korea | 75 527 | 1911 | No CVD (n = 101) | CVD (n = 1810) | Severity grade 1 | 0.98 (0.62–1.56) | .9361 |
|  |  |  |  |  | No IHD (n = 192) | IHD (n = 1719) |  | 0.99 (0.71–1.38) | .9361 |
|  |  |  |  |  | No CVD (n = 101) | CVD (n = 1810) | Severity grade 2 | 0.93 (0.48–1.78) | .8206 |
|  |  |  |  |  | No IHD (n = 192) | IHD (n = 1719) |  | 0.97 (0.62–1.53) | .8956 |
|  |  |  |  |  | No CVD (n = 101) | CVD (n = 1810) | Mortality | 0.88 (0.43–1.77) | .7089 |
|  |  |  |  |  | No IHD (n = 192) | IHD (n = 1719) |  | 0.93 (0.57–1.52) | .7828 |
| Kim[106] | 2022 | South Korea | 129 120 | 8070 | No CVD (n = NR) | CVD (n = 1330) | Severe COVID-19 | **1.54 (1.17–2.04)** | NR |
| Kim[62] | 2020 | South Korea | 2959 | 2959 | No HF (n = 2925) | HF (n = 34) | Severe COVID-19 | 1.81 (0.40–8.27) | NR |
|  |  |  |  |  | No CAD (n = 2847) | CAD (n = 112) | Severe COVID-19 | 0.66 (0.19–2.23) | NR |
| Lee[64] | 2020 | South Korea | 5061 | 5061 | No CVD (n = 5012) | CVD (n = 49) | Mortality | **2.32 (1.05–5.09)** | .037 |
| Lee[66] | 2020 | South Korea | 4742 | 4742 | No IHD (n = 4545) | IHD (n = 197) | ICU admission | 0.88 (0.39–2.02) | NR |
|  |  |  |  | 80^f^ | No IHD (n = 73) | IHD (n = 7) | Ventilator usage | 1.1 (0.1–12.0) | NR |
| Lee[68] | 2020 | South Korea | 7272 | 7272 | No IHD (n = 6958) | IHD (n = 314) | Respiratory failure | 0.88 (0.47–1.63) | .694 |
|  |  |  |  |  | No HF (n = 7118) | HF (n = 154) |  | 1.50 (0.74–3.05) | .256 |
|  |  |  |  |  | No IHD (n = 6958) | IHD (n = 314) | Mortality | 0.90 (0.57–1.44) | .686 |
|  |  |  |  |  | No HF (n = 7118) | HF (n = 154) |  | **1.98 (1.17–3.33)** | .01 |
| Lee[70] | 2020 | South Korea | 7339 | 7339 | No cardiovascular/cerebrovascular diseases (n = 6884) | Cardiovascular/cerebrovascular diseases (n = 455) | Severe COVID-19 | 0.98 (0.75–1.27) | .847 |
|  |  |  |  |  |  |  | Mortality | 0.82 (0.56–1.22) | .33 |
| Oh[76] | 2022 | South Korea | 5077 | 2106^g^ | No HF (n = 2086) | HF (n = 20) | Clinical severity | 1.78 (0.55–5.74) | NR |
|  |  |  |  |  | No chronic cardiac disease (n = 2020) | Chronic cardiac disease (n = 86) |  | 1.36 (0.68–2.72) | NR |
|  |  |  |  | 2971^h^ | No HF (n = 2935) | HF (n = 36) |  | 2.04 (0.82–5.04) | NR |
|  |  |  |  |  | No chronic cardiac disease (n = 2885) | Chronic cardiac disease (n = 86) |  | 1.01 (0.42–2.46) | NR |
| Oh[77] | 2021 | South Korea | 122 040 | 7780 | No peripheral vascular disease (n = NR) | Peripheral vascular disease (n = NR) | Mortality | 1.19 (0.81–1.76) | .76 |
|  |  |  |  |  | No congestive HF (n = NR) | Congestive HF (n = NR) |  | **1.91 (1.38–2.66)** | <.001 |
|  |  |  |  |  | No MI (n = NR) | MI (n = NR) |  | 0.79 (0.47–1.33) | .374 |
| Oh[78] | 2021 | South Korea | 122 040 | 7669 | No peripheral vascular disease (n = 7215) | Peripheral vascular disease (n = 454) | Mortality | 1.13 (0.76–1.69) | .549 |
|  |  |  |  |  | No congestive HF (n = 7001) | Congestive HF (n = 668) |  | **1.89 (1.35–2.66)** | <.001 |
|  |  |  |  |  | No MI (n = 7325) | MI (n = 344) |  | 0.96 (0.56–1.64) | .879 |
| Park[80] | 2020 | South Korea | 2269 | 2269 | No CV risk/CVD (n = 1315) | With CV risk/CVD (n = 954) | Mortality | **1.79 (1.07–3.01)** | .027 |
| Shin[84] | 2021 | South Korea | 5571 | 5571 | No HF (n = 5513) | HF (n = 58) | Severe COVID-19 | 1.96 (0.90–4.28) | .09 |
|  |  |  |  |  | No cardiac conduction disease (n = 5392) | Cardiac conduction disease (n = 179) |  | 1.03 (0.64–1.63) | .91 |
|  |  |  |  |  | No HF (n = 5513) | HF (n = 58) | Mortality | **3.15 (1.22–8.15)** | .02 |
|  |  |  |  |  | No cardiac conduction disease (n = 5392) | Cardiac conduction disease (n = 179) |  | 0.94 (0.46–1.89) | .86 |
| Song[85] | 2021 | South Korea | 5621 | 5621 | No HF (n = 5563) | HF (n = 58) | Mortality^i^ | 2.94 (0.68–11.6) | .133 |
|  |  |  |  |  |  |  | Fatal adverse outcome^i,k^ | 2.46 (0.63–8.5) | .174 |
|  |  |  |  |  |  |  | Mortality^j^ | 1.76 (0.8–3.83) | .158 |
|  |  |  |  |  | No cardiac disorder (n = 5442) | Cardiac disorder (n = 179) | Mortality^j^ | 1.34 (0.64–2.81) | .433 |
|  |  |  |  |  | No HF (n = 5563) | HF (n = 58) | Fatal adverse outcome^j,k^ | 1.90 (0.92–3.92) | .083 |
|  |  |  |  |  | No cardiac disorder (n = 5442) | Cardiac disorder (n = 179) | Fatal adverse outcome^j,k^ | 1.69 (0.89–3.22) | .111 |
| Huang[88] | 2022 | Taiwan | 239 | 239 | No CAD (n = 204) | CAD (n = 35) | Mortality | 1.76 (0.49–6.24) | .378 |
| Sano[112] | 2022 | Japan | 673 | 673 | Preexisting AF (n = 618) | Preexisting AF (n = 55) | In-hospital mortality | 2.09 (0.90–4.82) | .09 |

Abbreviations: AF, atrial fibrillation; CAD, coronary artery disease; CVD, cardiovascular disease; ECMO, extracorporeal membrane oxygenation; HF, heart failure; HR, hazard ratio; ICU, intensive care unit; IHD, ischemic heart disease; IMV, invasive mechanical ventilation; MV, mechanical ventilation; NR, not reported; OR, odds ratio.

Diseases of Circulatory system 3: Acute myocardial infarction; other ischemic heart diseases; conduction disorders and cardiac arrhythmias; heart failure; other heart diseases; Diseases of Circulatory system 5: Pulmonary embolism; atherosclerosis; other peripheral vascular diseases; arterial embolism and thrombosis; other diseases of arteries, arterioles and capillaries; phlebitis, thrombophlebitis, venous embolism and thrombosis.

^a^Paediatric and adult patients. ^b^Residential aged care facility residents. ^c^Hospitalization or death. ^d^ICU or death. ^e^Aged ≥50 years. ^f^ICU-admitted population. ^g^Male. ^h^Female. ^i^Conditional logistic regression. ^j^IMV, multiorgan failure, ECMO, and death. ^k^Cox proportional hazard.

### Supplementary Table 8. Studies assessing cerebrovascular disease

| **First author** | **Year** | **Country** | **Total** | **Cohort size** | **Reference**  **(sample size)** | **Comparator**  **(sample size)** | **Outcome** | **OR/HR (95% CI)** | ***P*-value** |
| --- | --- | --- | --- | --- | --- | --- | --- | --- | --- |
| Bhatia[1] | 2021 | Australia | 546 | 546 | No stroke or TIA (n = 506) | Stroke or TIA (n = 40) | Mortality | 1.33 (0.57– 3.07) | .51 |
| Ellis[3] | 2023 | Australia | 1071^a^ | 1071 | No cerebrovascular disease (n = 663) | Cerebrovascular disease (n = 251) | Hospitalization | 1.1 (0.7–1.6) | NR |
|  |  |  |  |  |  |  | Mortality | **1.6 (1.0–2.4)** | NR |
| Liu[4] | 2021 | Australia | 4054 | 4054 | No cerebrovascular disease (n = 4013) | Cerebrovascular disease (n = 41) | Severe COVID-19^b^ | 1 48 (0.93–2.37) | NR |
|  |  |  |  |  |  |  | Very severe COVID-19^c^ | 1.28 (0.65–2.52) | NR |
| Zhou[13] | 2023 | Hong Kong | 6089 | 6089 | No stroke or TIA (n = 5968) | Stroke or TIA (n = 121) | Severe COVID-19 | **8.85 (5.86–13.4)** | <.0001 |
| Miyashita[20] | 2022 | Japan | 937 758 | 937 758 | No cerebrovascular disease (n = 883 708) | Cerebrovascular disease (n = 54 050) | Severe COVID-19 | **1.06 (1.02–1.09)** | .001 |
|  |  |  |  |  |  |  | Mortality | **1.08 (1.04–1.13)** | <.001 |
| Nishimura[24] | 2023 | Japan | 30 130 | 1097 | No cerebrovascular disease (n = 373) | Cerebrovascular disease (n = 110) | Hospitalization | **2.29 (1.12–4.66)** | .023 |
|  |  |  |  |  |  |  | Oxygen administration | **2.54 (1.19–5.43)** | .016 |
| Nojiri[91] | 2023 | Japan | 11 440 | 11 440 | No cerebrovascular disease (n = 11 225) | Cerebrovascular disease (n = 215) | Severe COVID-19 | 0.63 (0.44–0.89) | .01 |
|  |  |  |  | 5980^d^ | No cerebrovascular disease (n = 5735) | Cerebrovascular disease (n = 215) | Mortality | 0.55 (0.27–1.05) | .081 |
| Sato[29] | 2022 | Japan | 500 | 500 | No history of cerebrovascular or cardiovascular disease (n = 462) | History of cerebrovascular or cardiovascular disease (n = 38) | Mortality or ECMO or IMV or MV or ICU admission | 1.32 (0.48–3.66) | .59 |
| Yamada[32] | 2021 | Japan | 6873 | 1007 | No cerebrovascular disease (n = NR) | Cerebrovascular disease (n = NR) | Oxygen therapy | **1.66 (1.02–2.68)** | .04 |
| Byeon[42] | 2021 | South Korea | 12 646 | 12 646 | No diseases of the circulatory system 4 (n = 11 696) | Diseases of the circulatory system 4 (n = 647) | Mortality | 1.16 (0.88–1.52) | NR |
| Chang[45] | 2022 | South Korea | 3887 | 3887 | No stroke (n = 3731) | Stroke (n = 156) | Severe COVID-19 | 1.25 (0.80–1.96) | .326 |
| Chang[46] | 2022 | South Korea | 3122 | 3122 | No stroke (n = 2996) | Stroke (n = 126) | Severe COVID-19 | 1.37 (0.82–2.28) | .231 |
| Choi[49] | 2021 | South Korea | 7590 | 7590 | No cerebrovascular disease (n = 6803) | Cerebrovascular disease (n = 787) | Mortality | 0.78 (0.54–1.13) | .184 |
|  |  |  |  |  |  |  | ICU admission | 1.03 (0.71–1.51) | .876 |
| Hwang[54] | 2020 | South Korea | 103 | 103 | No stroke (n = 99) | Stroke (n = 4) | Mortality | 0.28 (0.02–3.75) | .335 |
| Ji[104] | 2020 | South Korea | 219 961 | 7341 | No transient cerebral ischemia, stroke, cerebral hemorrhage (n = 6854) | Transient cerebral ischemia, stroke, cerebral hemorrhage (n = 487) | Severe COVID-19 | 0.87 (0.67–1.13) | NR |
| Kim[60] | 2021 | South Korea | 5527 | 1911 | No cerebrovascular disease (n = 279) | Cerebrovascular disease (n = 1632) | Severity grade 1 | 1.16 (0.87–1.54) | .3179 |
|  |  |  |  |  |  |  | Severity grade 2 | 1.08 (0.73–1.58) | .7028 |
|  |  |  |  |  |  |  | Mortality | 1.23 (0.82–1.84) | .3121 |
| Kim[106] | 2022 | South Korea | 129 120 | 8070 | No stroke (n = NR) | Stroke (n = 238) | Severe COVID-19 | 1.36 (0.72–2.54) | NR |
| Lee[64] | 2020 | South Korea | 5061 | 5061 | No cerebrovascular disease (n = 5042) | Cerebrovascular disease (n = 19) | Mortality | 0.47 (0.07–3.44) | .458 |
| Lee[70] | 2020 | South Korea | 7339 | 7339 | No cardiovascular/ cerebrovascular diseases (n = 6884) | Cardiovascular/ cerebrovascular diseases (n = 455) | Severe COVID-19 | 0.98 (0.75–1.27) | .847 |
|  |  |  |  |  |  |  | Mortality | 0.82 (0.56–1.22) | .33 |
| Oh[77] | 2021 | South Korea | 122 040 | 7780 | No cerebrovascular disease (n = NR) | Cerebrovascular disease (n = NR) | Mortality | 0.57 (0.38–0.87) | .009 |
| Oh[78] | 2021 | South Korea | 122 040 | 7669 | No cerebrovascular disease (n = 7191) | Cerebrovascular disease (n = 478) | Mortality | 0.73 (0.48–1.10) | .131 |
| Seong[83] | 2021 | South Korea | 488 | 318 | No cerebrovascular disease (n = 276) | Cerebrovascular disease (n = 42) | Mortality | 1.49 (0.78–2.85) | .227 |
| Yun[87] | 2020 | South Korea | 7590 | 7363 | No cerebrovascular disease (n = 6730) | Cerebrovascular disease (n = 633) | Severe COVID-19 | 0.87 (0.64–1.18) | .37 |
| Huang[88] | 2022 | Taiwan | 239 | 239 | No cerebrovascular accident (n = 227) | Cerebrovascular accident (n = 12) | Mortality | **3.62 (1.25–10.4)** | .017 |

Abbreviations: ECMO, extracorporeal membrane oxygenation; HFNC, high-flow nasal cannula; HR, hazard ratio; ICU, intensive care unit; IMV, invasive mechanical ventilation; MV, mechanical ventilation; NR, not reported; OR, odds ratio; TIA, transient ischemic attack.

Diseases of the circulatory system 4: Intracranial hemorrhage; cerebral infarction; stroke, not speciﬁed as hemorrhage or infarction; other cerebrovascular diseases.

^a^Aged care facility residents. ^b^Hospitalization or death. ^c^ICU admission or death. ^d^Aged ≥50 years.

### Supplementary Table 9. Studies assessing renal disease including chronic kidney disease

| **First author** | **Year** | **Country** | **Total** | **Cohort size** | **Reference (sample size)** | **Comparator (sample size)** | **Outcome** | **OR/HR (95% CI)** | ***P*-value** |
| --- | --- | --- | --- | --- | --- | --- | --- | --- | --- |
| Bhatia[1] | 2021 | Australia | 546 | 546 | No CKD (eGFR ≥60) (n = 500) | CKD (eGFR <60) (n = 46) | Mortality | **2.33 (1.02–5.32)** | .044 |
| Ellis[3] | 2023 | Australia | 1071^a^ | 1071 | No CKD (n = 676) | CKD (n = 237) | Hospitalization | **1.7 (1.1–2.6)** | NR |
|  |  |  |  |  |  |  | Mortality | 1.5 (0.9–2.3) | NR |
| Liu[4] | 2021 | Australia | 4054 | 4054 | No CKD (n = 4020) | CKD (n = 34) | Severe COVID-19^b^ | 1.38 (0.82–2.31) | NR |
|  |  |  |  |  |  |  | Very severe COVID-19^c^ | 1.87 (0.95–3.68) | NR |
| Fan[9] | 2021 | Hong Kong | 4834 | 3164 | No renal disease (n = NR) | Renal disease (n = NR) | Mortality | **2.68 (1.62–4.44)** | <.001 |
| Yip[12] | 2021 | Hong Kong | 5639 | 5639 | No kidney disease (n = 5501) | Kidney disease (n = 138) | Mortality | **2.27 (1.48–3.48)** | <.001 |
| Zhou[14] | 2020 | Hong Kong | 1043 | 1043 | No CKD (n = 1022) | CKD (n = 21) | ICU admission | **4.87 (2.66–9.71)** | .0009 |
| Zhou[15] | 2020 | Hong Kong | 2774 | 2774 | No kidney disease (n = 2641) | Kidney disease (n = 133) | Mortality | 1.25 (0.42–2.88) | .0032 |
| Fukushima[17] | 2023 | Japan | 2430 | 2430 | No CKD (n = 2239) | CKD (n = 191) | Critical events | **2.82 (1.85–4.30)** | <.0001 |
| Kurahara[102] | 2021 | Japan | 404 | 404 | No CKD (n = 373) | CKD (n = 31) | Acute respiratory failure requiring oxygenation | 1.45 (0.67–3.14) | .347 |
| Miyashita[20] | 2022 | Japan | 937 758 | 937 758 | No renal disease (n = 917 120) | Renal disease (n = 20 638) | Severe COVID-19 | **1.48 (1.42–1.55)** | <.001 |
|  |  |  |  |  |  |  | Mortality | **1.67 (1.59–1.75)** | <.001 |
| Nishimura[24] | 2023 | Japan | 30 130 | 1097 | No CKD (n = 394) | CKD (n = 89) | Hospitalization | 0.70 (0.34–1.46) | .339 |
|  |  |  |  |  |  |  | Oxygen administration | 1.01 (0.48–2.09) | .983 |
| Nojiri[91] | 2023 | Japan | 11 440 | 11 440 | No CKD (n = 11 348) | CKD (n = 92) | Severe COVID-19 | **1.6 (1.01–2.53)** | .043 |
|  |  |  |  | 5980^d^ | No CKD (n = NR) | CKD (n = NR) | Mortality | 2.2 (0.99–4.58) | .042 |
| Sato[29] | 2022 | Japan | 500 | 500 | No kidney dysfunction (n = 329) | Kidney dysfunction (n = 171) | Severe COVID-19 | **2.35 (1.14–4.86)** | .02 |
| Bae[41] | 2021 | South Korea | 1760 | 1232 | No CKD (n = 1206) | CKD (n = 26) | Mortality | **2.35 (1.25–4.43)** | .008 |
| Chang[45] | 2022 | South Korea | 3887 | 3887 | No CKD (n = 3609) | CKD (n = 278) | Severe COVID-19 | 1.27 (0.86–1.88) | .225 |
| Chang[46] | 2022 | South Korea | 3122 | 3122 | No CKD (n = 2921) | CKD (n = 201) | Severe COVID-19 | 1.21 (0.76–1.92) | .429 |
| Cho[47] | 2021 | South Korea | 7590 | 7590 | No renal disease (n = 7531) | Renal disease (n = 59) | Mortality | **4.95 (2.37–10.3)** | <.001 |
| Cho[48] | 2021 | South Korea | 5594 | 3729 | No chronic renal failure (n = 3691) | Chronic renal failure (n = 36) | Overall survival | **3.48 (1.39–8.85)** | .045 |
| Cho[92] | 2021 | South Korea | 1272 | 1272 | No CKD (n = 1246) | CKD (n = 26) | Mortality | **3.55 (1.30–9.20)** | NR |
|  |  |  |  |  |  |  | Severe COVID-19 | **4.45 (1.73–11.2)** | NR |
| Choi[49] | 2021 | South Korea | 7590 | 7590 | No renal disease (n = 7428) | Renal disease (n = 162) | Mortality | **2.3 (1.37–3.86)** | .002 |
|  |  |  |  |  |  |  | ICU admission | 1.43 (0.81–2.53) | .213 |
| Huh[52] | 2021 | South Korea | 36 705 | 2805 | No CKD (n = 2653) | CKD (n = 152) | Severe COVID-19 | **1.51 (1.05–2.17)** | .02 |
| Ji[104] | 2020 | South Korea | 219 961 | 7341 | No hypertensive renal disease (n = 7322) | Hypertensive renal disease (n = 19) | Severe COVID-19 | 1.46 (0.48–4.46) | NR |
|  |  |  |  |  | No glomerular disease (n = 7265) | Glomerular disease (n = 76) |  | 1.03 (0.55–1.94) | NR |
|  |  |  |  |  | No renal tubular interstitial disease (n = 7304) | Renal tubular interstitial disease (n = 37) |  | 1.62 (0.7–3.77) | NR |
|  |  |  |  |  | No CKD and ESRD (n = 7269) | CKD and ESRD (n = 72) |  | **2.11 (1.16–3.83)** | NR |
| Kang[57] | 2021 | South Korea | 4141 | 3827 | No CKD (n = 4097) | CKD (n = 44) | Severe COVID-19 | **3.12 (1.36–7.19)** | NR |
|  |  |  |  |  |  |  | Mortality | **2.74 (1.11–6.76)** | NR |
| Kang[58] | 2020 | South Korea | 7341 | 7341 | No CKD (n = 7088) | Nondialysis CKD (n = 239) | Mortality | 0.79 (0.49–1.26) | .318 |
|  |  |  |  |  | No CKD (n = 7088) | Dialysis-dependent CKD (n = 14) |  | **2.96 (1.09–8.06)** | .033 |
|  |  |  |  |  | Nondialysis CKD (n = 239) | Dialysis-dependent CKD (n = 14) |  | **3.77 (1.29–11.1)** | .016 |
|  |  |  |  |  | No CKD (n = 7088) | Nondialysis CKD (n = 239) | Oxygen therapy | 1.01 (0.72–1.42) | .944 |
|  |  |  |  |  | No CKD (n = 7088) | Dialysis-dependent CKD (n = 14) |  | **4.80 (1.51–6.11)** | .008 |
|  |  |  |  |  | Nondialysis CKD (n = 239) | Dialysis-dependent CKD (n = 14) |  | **4.74 (1.45–16.4)** | .011 |
|  |  |  |  |  | No CKD (n = 7088) | Nondialysis CKD (n = 239) | IMV | 0.61 (0.27–1.25) | .207 |
|  |  |  |  |  | No CKD (n = 7088) | Dialysis-dependent CKD (n = 14) |  | **9.82 (2.47–33.2)** | <.001 |
|  |  |  |  |  | Nondialysis CKD (n = 239) | Dialysis-dependent CKD (n = 14) |  | **16 (3.59–64.5)** | <.001 |
|  |  |  |  |  | No CKD (n = 7088) | Nondialysis CKD (n = 239) | ECMO | 0.4 (0.02–2.49) | .443 |
|  |  |  |  |  | No CKD (n = 7088) | Dialysis-dependent CKD (n = 14) |  | 8.62 (0.44–54.1) | .053 |
|  |  |  |  |  | Nondialysis CKD (n = 239) | Dialysis-dependent CKD (n = 14) |  | 19.8 (0.74–533) | .041 |
|  |  |  |  |  | No CKD (n = 7088) | Nondialysis CKD (n = 239) | Cardiac arrest | 0.70 (0.22–1.86) | .508 |
|  |  |  |  |  | No CKD (n = 7088) | Dialysis-dependent CKD (n = 14) |  | 5.24 (0.27–31.0) | .129 |
|  |  |  |  |  | Nondialysis CKD (n = 239) | Dialysis-dependent CKD (n = 14) |  | 7.47 (0.36–56.8) | .086 |
|  |  |  |  |  | No CKD (n = 7088) | Nondialysis CKD (n = 239) | Myocardial infarction | 1.33 (0.73–2.32) | .331 |
|  |  |  |  |  |  | Dialysis-dependent CKD (n = 14) |  | 1.44 (0.08–7.53) | .727 |
|  |  |  |  |  | Nondialysis CKD (n = 239) | Dialysis-dependent CKD (n = 14) |  | 1.08 (0.06–6.03) | .94 |
|  |  |  |  |  | No CKD (n = 7088) | Nondialysis CKD (n = 239) | Acute heart failure | 0.74 (0.43–1.23) | .264 |
|  |  |  |  |  |  | Dialysis-dependent CKD (n = 14) |  | 1.57 (0.24–6.05) | .562 |
|  |  |  |  |  | Nondialysis CKD (n = 239) | Dialysis-dependent CKD (n = 14) |  | 2.12 (0.31–8.80) | .354 |
| Kim[59] | 2020 | South Korea | 9148 | 9148 | No renal tubulointerstitial diseases (n = 8870) | Renal tubulointerstitial diseases (n = 278) | Mortality | **2.48 (1.36–4.53)** | NR |
|  |  |  |  |  | No renal failure (n = 9076) | Renal failure (n = 72) | Mortality | **3.07 (1.43–6.61)** | NR |
| Kim[106] | 2022 | South Korea | 129 120 | 8070 | No kidney disease (n = NR) | Kidney disease (n = 172) | Severe COVID-19 | **5.59 (2.48–12.6)** | NR |
| Kim[62] | 2020 | South Korea | 2959 | 2959 | No CKD (n = 2922) | CKD (n = 37) | Severe COVID-19 | 2.68 (0.65–11.1) | NR |
| Lee[64] | 2020 | South Korea | 5061 | 5061 | No CKD (n = 5036) | CKD (n = 25) | Mortality | 1.26 (0.31–5.18) | .752 |
| Lee[70] | 2020 | South Korea | 7339 | 7339 | No CKD (n = 7291) | CKD (n = 48) | Severe COVID-19 | **3.40 (1.67–6.92)** | .001 |
|  |  |  |  |  |  |  | Mortality | **3.11 (1.33–7.3)** | .009 |
| Lee[71] | 2020 | South Korea | 1005 | 1005 | No chronic renal disease (n = 987) | Chronic renal disease (n = 16) | Severe COVID-19 | **4.20 (1.20–14.7)** | .025 |
|  |  |  |  |  |  |  | Mortality | **4.52 (1.85–11.0)** | 0.001 |
| Oh[76] | 2022 | South Korea | 5077 | 2106^e^ | No CKD (n = 2080) | CKD (n = 26) | Clinical severity | 1.77 (0.60–5.26) | NR |
|  |  |  |  | 2971^f^ | No CKD (n = 2943) | CKD (n = 28) |  | **5.09 (1.87–13.9)** | .001 |
| Oh[77] | 2021 | South Korea | 122 040 | 7780 | No renal disease (n = NR) | Renal disease (n = NR) | Mortality | 1.47 (0.87–2.47) | .148 |
| Oh[78] | 2021 | South Korea | 122 040 | 7669 | No renal disease (n = 7537) | Renal disease (n = 132) | Mortality | 1.57 (0.93–2.64) | .093 |
| Park[80] | 2020 | Korea | 2269 | 2269 | No CKD (n = 2232) | CKD (n = 37) | Mortality | 2.19 (0.85–5.63) | .104 |
| Shin[84] | 2021 | South Korea | 5571 | 5571 | No CKD (n = 5516) | CKD (n = 55) | Severe COVID-19 | **2.58 (1.19–5.61)** | .01 |
|  |  |  |  |  |  |  | Mortality | **5.35 (2.00–14.3)** | <.01 |
| Song[85] | 2021 | South Korea | 5621 | 5621 | No CKD (n = 5596) | CKD (n = 55) | Mortality | 1.52 (0.68–3.42) | .312 |
|  |  |  |  |  |  |  | Fatal adverse outcome^g^ | 1.55 (0.72–3.32) | .261 |
| Wang[86] | 2021 | South Korea | 2800^d^ | 446 | No CKD (n = 423) | CKD (n = 23) | Mortality | **4.62 (1.56–14.6)** | .007 |
| Huang[88] | 2022 | Taiwan | 239 | 239 | No CKD (n = 218) | CKD (n = 21) | Mortality | 1.31 (0.32–5.30) | .697 |

Abbreviations: CKD, chronic kidney disease; ECMO, extracorporeal membrane oxygenation; eGFR, estimated glomerular filtration rate (mL/min/1.73 m^2^); ESRD, end-stage renal disease; HFNC, high-flow nasal cannula; HR, hazard ratio; ICU, intensive care unit; IMV, invasive mechanical ventilation; MV, mechanical ventilation; NR, not reported; OR, odds ratio.

^a^Residential aged care facility residents. ^b^Hospitalization or death. ^c^ICU admission or death. ^d^Aged ≥50 years. ^e^Male. ^f^Female. ^g^IMV, multiorgan failure, ECMO, and death.

### Supplementary Table 10. Studies assessing hepatobiliary disorders

| **First author** | **Year** | **Country** | **Total** | **Cohort size** | **Reference  (sample size)** | **Comparator  (sample size)** | **Outcome** | **OR/HR (95%CI)** | ***P*-value** |
| --- | --- | --- | --- | --- | --- | --- | --- | --- | --- |
| Yip[12] | 2021 | Hong Kong | 5639 | 5639 | No liver cirrhosis (n = 5560) | Liver cirrhosis (n = 79) | Mortality | **2.36 (1.20–4.63)** | .013 |
|  |  |  |  |  | No HBV (n = 4927) | Current HBV infection (n = 353) | Mortality | 1.09 (0.52–2.27) | .829 |
|  |  |  |  |  |  | Past HBV infection (n = 359) | Mortality | 1.05 (0.65–1.69) | .836 |
| Zhou[13] | 2023 | Hong Kong | 6089 | 6089 | No liver disease (n = 6057) | Liver diseases (n = 32) | Severe COVID-19 | 2.16 (0.54–8.72) | .278 |
| Fukushima[17] | 2023 | Japan | 2430 | 2430 | No chronic liver disease  (n  = 2319) | Chronic liver disease (n = 111) | Critical events | 0.83 (0.40–1.73) | .63 |
| Miyashita[20] | 2022 | Japan | 937 758 | 937 758 | No liver disease (n = 869 960) | Liver disease (n = 77 601) | Severe COVID-19 | **1.07 (1.04–1.11)** | <.001 |
|  |  |  |  |  | No liver disease (n = 869 960) | Liver disease (n = 77 601) | Mortality | 0.96 (0.92–1.00) | .04 |
| Nishimura[24] | 2023 | Japan | 30 130 | 1097 | No chronic hepatitis or cirrhosis (n = 379) | Chronic hepatitis or cirrhosis  (n = 104) | Hospitalization | 1.84 (0.85–3.97) | .122 |
|  |  |  |  |  | No chronic hepatitis or cirrhosis (n = 355) | Chronic hepatitis or cirrhosis  (n = 106) | Oxygen therapy | 0.55 (0.24–1.24) | .148 |
| Sakamoto[28] | 2022 | Japan | 6176 | 6176 | No liver disease (n = 6160) | Liver disease (n = 16) | Hospitalization | 2.32 (0.56–9.58) | NR |
| Cho[47] | 2021 | South Korea | 7590 | 7590 | No liver disease (n = 6982) | Liver disease (n = 608) | Mortality | **1.50 (1.02–2.20)** | .040 |
| Choi[49] | 2021 | South Korea | 7590 | 7590 | No mild liver disease (n = 4843) | Mild liver disease (n = 2747) | Mortality | 0.856 (0.610–1.200) | .366 |
|  |  |  |  |  | No moderate or severe liver disease (n = 7564) | Moderate or severe liver disease (n = 26) | Mortality | 1.482 (0.328–6.694) | .609 |
|  |  |  |  |  | No mild liver disease (n = 4843) | Mild liver disease (n = 2747) | ICU admission | 0.985 (0.718–1.352) | .926 |
|  |  |  |  |  | No moderate or severe liver disease (n = 7564) | Moderate or severe liver disease (n = 26) | ICU admission | 1.364 (0.301–6.188) | .687 |
| Huh[52] | 2021 | South Korea | 7341 | 2805 | No chronic liver disease  (n = 1857) | Chronic liver disease (n = 948) | Severe COVID-19 | 0.88 (0.73–1.07) | .2 |
| Jeon[113] | 2021 | South Korea | 234 427 | 732 | Liver cirrhosis (n = 67) | No liver cirrhosis (n = 333) | Severe COVID-19 | 1.082 (0.416–2.817) | .870 |
|  |  |  |  |  | Liver cirrhosis (n = 67) | No liver cirrhosis (n = 333) | Oxygen therapy | 1.095 (0.421–2.844) | .850 |
|  |  |  |  |  | Liver cirrhosis (n = 67) | No liver cirrhosis (n = 333) | Mortality | 6.426 (0.733–56.368) | .090 |
| Ji[104] | 2020 | South Korea | 219 961 | 7341 | No hepatitis B virus acute and chronic (n = 7226) | Hepatitis B virus acute and chronic (n = 115) | Severe COVID-19 | 1.584 (0.920–2.728) | NR |
|  |  |  |  |  | No hepatitis C virus acute and chronic (n = 7324) | Hepatitis C virus acute and chronic (n = 17) | Severe COVID-19 | 0.473 (0.120–1.861) | NR |
|  |  |  |  |  | No non-B, non-C hepatitis  (n = 6729) | Non-B, non-C hepatitis (n = 612) | Severe COVID-19 | 0.834 (0.643–1.080) | NR |
|  |  |  |  |  | No liver cirrhosis (n = 7297) | Liver cirrhosis (n = 44) | Severe COVID-19 | 1.031 (0.469–2.265) | NR |
|  |  |  |  |  | No hepatic failure (n = 7335) | Hepatic failure (n = 6) | Severe COVID-19 | 0.592 (0.082–4.289) | NR |
|  |  |  |  |  | No biliary disease (n = 7212) | Biliary disease (n = 129) | Severe COVID-19 | 1.186 (0.744–1.892) | NR |
| Kim[106] | 2022 | South Korea | 129 120 | 8070 | No hepatobiliary disease (n = NR) | Hepatobiliary disease (n = 2152) | Severe COVID-19 | 1.01 (0.78–1.31) | NR |
| Kim[62] | 2020 | South Korea | 2959 | 2959 | No chronic liver disease  (n = 2913) | Chronic liver disease (n = 46) | Severe COVID-19 | 0.14 (0.01–3.06) | NR |
| Lee[70] | 2020 | South Korea | 7339 | 7339 | No chronic liver disease  (n = 6694) | Chronic liver disease (n = 645) | Severe COVID-19 | 0.86 (0.66–1.13) | .291 |
|  |  |  |  |  | No chronic liver disease  (n = 6694) | Chronic liver disease (n = 645) | Mortality | 0.87 (0.50–1.51) | .609 |
| Lee[71] | 2020 | South Korea | 1005 | 1005 | No liver cirrhosis (n = 958) | Liver cirrhosis (n = 47) | Severe COVID-19 | **4.52 (1.20–17.02)** | .026 |
|  |  |  |  |  | No liver cirrhosis (n = 958) | Liver cirrhosis (n = 47) | Mortality | **2.86 (1.04–9.30)** | .042 |
| Oh[76] | 2022 | South Korea | 5077 | 2106^a^ | No chronic liver disease  (n = 2060) | Chronic liver disease (n = 46) | Clinical severity | 1.22 (0.45–3.32) | NR |
|  |  |  |  | 2971^b^ | No chronic liver disease  (n = 2937) | Chronic liver disease (n = 34) | Clinical severity | 0.56 (0.07–4.46) | NR |
| Oh[77] | 2021 | South Korea | 122 040 | 7780 | No moderate or severe liver disease (n = NR) | Moderate or severe liver disease (n = NR) | Mortality | **5.12 (1.32–19.9)** | .018 |
|  |  |  |  |  | No mild liver disease (n = NR) | Mild liver disease (n = NR) | Mortality | 0.80 (0.58–1.10) | .17 |
| Oh[78] | 2021 | South Korea | 122 040 | 7669 | No moderate or severe liver disease (n = 7654) | Moderate or severe liver disease (n = 15) | Mortality | 3.50 (0.87–14.12) | .078 |
|  |  |  |  |  | No mild liver disease (n = 5924) | Mild liver disease (n = 1745) | Mortality | 0.78 (0.56–1.09) | .15 |
| Seong[83] | 2021 | South Korea | 488 | 318^c^ | No liver cirrhosis (n = 314) | Liver cirrhosis (n = 4) | Mortality | 1.29 (0.27–6.15) | .752 |
| Shin[84] | 2021 | South Korea | 5571 | 5571 | No chronic liver disease  (n = 5166) | Chronic liver disease (n = 82) | Severe COVID-19 | 1.72 (0.87–3.40) | .12 |
|  |  |  |  |  | No chronic liver disease  (n = 5166) | Chronic liver disease (n = 82) | Mortality | 1.26 (0.32–5.02) | .74 |
| Yoo[114] | 2021 | South Korea | 76 495 | 34 842^d^ | No NAFLD (n = 17 421) | NAFLD (n = 17 421) | Severe COVID-19 | **1.41 (1.08–1.83)** | NR |
|  |  |  |  |  | No NAFLD (n = 17 421) | NAFLD (n = 17 421) | Mortality | 1.30 (0.55–3.09) | NR |
|  |  |  |  | 39 450^e^ | No NAFLD (n = 19 725) | NAFLD (n = 19 725) | Severe COVID-19 | **1.35 (1.05–1.71)** | NR |
|  |  |  |  |  | No NAFLD (n = 19 725) | NAFLD (n = 19 725) | Mortality | 1.65 (0.80–3.40) | NR |
|  |  |  |  | 17 660^f^ | No NAFLD (n = 8830) | NAFLD (n = 8830) | Severe COVID-19 | **1.39 (1.01–1.92**) | NR |
|  |  |  |  |  | No NAFLD (n = 8830) | NAFLD (n = 8830) | Mortality | 0.95 (0.34–2.62) | NR |

Abbreviations: HBV, hepatitis B virus; NAFLD, non-alcoholic fatty liver disease; NR, not reported.

^a^Male. ^b^Female. ^c^Aged ≥65 years. ^d^HSI-NAFLD (hepatic steatosis index) cohort. ^e^FLI-NAFLD (fatty liver index) cohort. ^f^Claim-based NAFLD cohort.

### Supplementary Table 11. Studies assessing cancer or malignancy

| **First author** | **Year** | **Country** | **Total** | **Cohort size** | **Reference  (sample size)** | **Comparator  (sample size)** | **Outcome** | **OR/HR (95% CI)** | | ***P*-value** |
| --- | --- | --- | --- | --- | --- | --- | --- | --- | --- | --- |
| Ellis[3] | 2023 | Australia | 1071^a^ | 1071 | No history of cancer  (n = 765) | History of cancer (n = 147) | Hospitalization | 1.3 (0.8–2.1) | | NR |
|  |  |  |  |  |  |  | Mortality | 1.4 (0.8–2.2) | | NR |
| Liu[4] | 2021 | Australia | 44 054 | 4054 | No cancer in the last year  (n = 4041) | Cancer in last year (n = 13) | Severe COVID-19^b^ | 2.23 (0.91–5.46) | | NR |
|  |  |  |  |  |  |  | Very severe COVID-19^c^ | 2.30 (0.72–7.40) | | NR |
| Fan[9] | 2021 | Hong Kong | 4834 | 3164 | No malignancy (n = NR) | Malignancy (n = NR) | Mortality | **1.86 (1.02–3.41)** | | .043 |
| Yip[12] | 2021 | Hong Kong | 5639 | 5639 | No malignant tumor  (n = 5395) | Malignant tumor (n = 244) | Mortality | **1.80 (1.17–2.77)** | | .007 |
| Zhou[13] | 2023 | Hong Kong | 6089 | 6089 | No cancer (n = 5947) | Any cancer (n = 142) | Severe COVID-19 | **3.77 (1.63–8.72)** | | .0019 |
|  |  |  |  |  |  | Lung cancer (n = 9) |  | 3.19 (0.88–11.5) | | .0766 |
|  |  |  |  |  |  | Gastrointestinal cancer  (n = 14) |  | **3.79 (1.12–12.9)** | | .0325 |
|  |  |  |  |  |  | Breast cancer (n = 27) |  | **2.73 (0.76–9.79)** | | .1222 |
|  |  |  |  |  |  | Genitourinary cancer (n = 8) |  | 2.63 (0.67–10.3) | | .1659 |
|  |  |  |  |  |  | Colorectal cancer (n = 11) |  | **5.07 (1.5–17.2)** | | .0091 |
|  |  |  |  |  |  | Other cancers (n = 85) |  | 1.34 (0.58–3.11) | | .4897 |
| Fukushima[16] | 2021 | Japan | 234 | 234 | No malignancy (n = 202) | Malignancy (n = 32) | Critical events | **6.74 (1.96–26.9)** | | NR |
| Kurahara[102] | 2021 | Japan | 404 | 404 | No malignant disease  (n = 380) | Malignant disease (n = 24) | Acute respiratory failure requiring oxygenation | 1.92 (0.8–4.65) | | .146 |
| Miyashita[20] | 2022 | Japan | 937 758 | 937 758 | No malignancy (n = 894 904) | Any malignancy (n = 42 854) | Severe COVID-19 | **1.07 (1.03–1.11)** | | < .001 |
|  |  |  |  |  |  |  | Mortality | **2.19 (1.99–2.42)** | | < .001 |
|  |  |  |  |  | No metastatic solid tumours (n = 934 044) | Metastatic solid tumours  (n = 3269) | Severe COVID-19 | **1.31 (1.25–1.37)** | | < .001 |
|  |  |  |  |  |  |  | Mortality | **3.31 (2.97–3.69)** | | < .001 |
| Muto[21] | 2021 | Japan | 300 | 300 | No malignancy (n = 271) | Malignancy (n = 29) | Critical/severe COVID-19 | 0.54 (0.19–1.54) | | .25 |
| Nishimura[24] | 2023 | Japan | 1097 | 483 | No malignant tumor  (n = 380) | Malignant tumor (n = 103) | Hospitalization | 0.46 (0.23–0.92) | | .029 |
|  |  |  |  | 461 | No malignant tumor  (n = 337) | Malignant tumor (n = 124) | Oxygen administration | **3.43 (1.69–6.97)** | | < .001 |
| Nojiri[91] | 2023 | Japan | 11 440 | 11 440 | No cancer (n = 11 047) | Cancer (n = 393) | Severe COVID-19 | 0.75 (0.58–0.95) | | .019 |
|  |  |  |  | 5980^d^ | No cancer (n = NR) | Cancer (n = NR) | Mortality | 0.95 (0.58–1.52) | | .8 |
| Takeyama[30] | 2022 | Japan | 2894 | 2894 | No active cancer (n = | Active cancer (n = 60) Mortality | **3.20 (1.44–7.09)** | .004 |  |  |
| Yamada[32] | 2021 | Japan | 6873 | 1891 | No malignancy (n = NR) | Malignancy (n = NR) | Oxygen therapy | **13.6 (1.33–138)** | | .028 |
| Jefferies[33] | 2020 | New Zealand | 1503 | 1495 | No malignancy (n = 1482) | Malignancy (n = 13) | Severe COVID-19 | 2.36 (0.52–10.8) | | NR |
| Espiritu[115] | 2022 | Philippine | 10 881 | 10 881 | No malignancy (n = 10 637) | Malignancy (n = 244) | Severe COVID-19 | **1.75 (1.32–2.33)** | | NR |
|  |  |  |  |  |  |  | Mortality | **1.72 (1.37–2.16)** | | NR |
|  |  |  |  |  |  |  | Respiratory failure | **1.65 (1.31–2.08)** | | NR |
|  |  |  |  |  |  |  | ICU admission | **1.57 (1.24–1.97)** | | NR |
| Bae[41] | 2021 | South Korea | 1760 | 1232 | No malignancy (n = 1186) | Malignancy (n = 46) | Mortality | 1.65 (0.87–3.14) | | .125 |
| Baek[116] | 2021 | South Korea | 6435 | 5186 | No malignancy (n = 1843) | Malignancy (n = 3343) | Mortality | 1.24 (0.92–1.66) | | .153 |
|  |  |  |  |  |  |  | Conventional oxygen therapy | 1.02 (0.88–1.18) | | .829 |
|  |  |  |  |  |  |  | High-flow nasal cannula | **1.42 (1.35–1.49)** | | .210 |
|  |  |  |  |  |  |  | IMV | **1.25 (1.17–1.34)** | | .043 |
|  |  |  |  |  |  |  | Vasopressor use | 1.23 (0.91–1.67) | | .179 |
|  |  |  |  |  |  |  | Renal replacement therapy | **1.51 (1.33–1.72)** | | .053 |
|  |  |  |  |  |  |  | Acute heart failure | **1.45 (1.21–1.75)** | | < .001 |
|  |  |  |  |  | No solid tumor (n = 1917) | Solid tumors (n = 3269) | Mortality | 1.26 (0.94–1.69) | | .120 |
|  |  |  |  |  |  |  | Conventional oxygen therapy | 1.03 (0.89–1.20) | | .653 |
|  |  |  |  |  |  |  | High-flow nasal cannula | 0.84 (0.62–1.13) | | .253 |
|  |  |  |  |  |  |  | IMV | 0.67 (0.45–1.00) | | .051 |
|  |  |  |  |  |  |  | Vasopressor use | 1.26 (0.93–1.71) | | .14 |
|  |  |  |  |  |  |  | Renal replacement therapy | 0.35 (0.12–1.03) | | .058 |
|  |  |  |  |  |  |  | Acute heart failure | **1.41 (1.17–1.71)** | | .003 |
| Byeon[42] | 2021 | South Korea | 12 646 | 12 646 | No neoplasms 1 (n = 12 313) | Neoplasms 1 (n = 30) | Mortality | **3.46 (1.83–6.56)** | | NR |
|  |  |  |  |  | No neoplasms 2 (n = 12 263) | Neoplasms 2 (n = 80) |  | **2.00 (1.17–3.41)** | | NR |
|  |  |  |  |  | No neoplasms 3 (n = 12 146) | Neoplasms 3 (n = 197) |  | 1.15 (0.59–2.22) | | NR |
|  |  |  |  |  | No neoplasms 4 (n = 12 322) | Neoplasms 4 (n = 21) |  | 2.17 (0.65–7.23) | | NR |
| Chang[45] | 2022 | South Korea | 3887 | 3887 | No malignancy (n = 3554) | Malignancy (n = 333) | Severe COVID-19 | 1.36 (0.93–1.99) | | .115 |
| Chang[46] | 2022 | South Korea | 3122 | 3122 | No malignancy (n = 2878) | Malignancy (n = 244) | Severe COVID-19 | 1.37 (0.88–2.14) | | .169 |
| Cho[47] | 2021 | South Korea | 7590 | 7590 | No cancer (n = 7327) | Cancer (n = 263) | Mortality | **1.88 (1.17–3.02)** | | .009 |
| Cho[48] | 2021 | South Korea | 5594 | 3729 | No cancer in active treatment (n = 3636) | Cancer in active treatment (n = 90) | Overall survival | 1.83 (0.72–4.71) | | .300 |
| Cho[92] | 2021 | South Korea | 1272 | 1272 | No cancer (n = 1219) | Cancer (n = 53) | Mortality | **3.54 (1.65–7.17)** | | NR |
|  |  |  |  |  |  |  | Severe COVID-19 | **2.68 (1.26–5.38)** | | NR |
| Choi[49] | 2021 | South Korea | 7590 | 7590 | No malignancy (n = 7033) | Malignancy (n = 557) | Mortality | **1.52 (1.02–2.26)** | | .039 |
|  |  |  |  |  |  |  | ICU admission | 1.41 (0.95–2.09) | | .138 |
|  |  |  |  |  | No metastatic solid tumor  (n = 7529) | Metastatic solid tumor  (n = 61) | Mortality | 2.11 (0.79–5.67) | | .092 |
|  |  |  |  |  |  |  | ICU admission | 1.81 (0.70–4.67) | | .219 |
| Chung[50] | 2020 | South Korea | 110 | 110 | No malignancy (n = 104) | Malignancy (n = 6) | Severe COVID-19 | 0.47 (0.03–6.73) | | .575 |
| Her[51] | 2021 | South Korea | 5628 | 3940 | No malignancy (n = 3839) | Malignancy (n = 101) | Mortality | **2.78 (1.14–6.79)** | | .025 |
| Huh[52] | 2021 | South Korea | 44 046 | 2805 | No malignancy (n = 2591) | Malignancy (n = 214) | Severe COVID-19 | 1.12 (0.82–1.54) | | .47 |
| Ji[104] | 2020 | South Korea | 432 581 | 7341 | No solid organ malignancy, except respiratory and thyroid (n = 7118) | Solid organ malignancy, except respiratory and thyroid (n = 223) | Severe COVID-19 | 0.95 (0.65–1.38) | | NR |
|  |  |  |  |  | No respiratory tract cancer (n = 7313) | Respiratory tract cancer (n = 28) |  | 1.89 (0.80–4.45) | | NR |
|  |  |  |  |  | No thyroid cancer (n = 7261) | Thyroid cancer (n = 80) |  | 0.83 (0.41–1.68) | | NR |
|  |  |  |  |  | No hematologic cancer  (n = 7333) | Hematologic cancer (n = 8) |  | 0.45 (0.06–3.57) | | NR |
| Kang[57] | 2021 | South Korea | 4141 | 4141 | No malignancy (n = 4034) | Malignancy (n = 107) | Severe COVID-19 | **2.28 (1.12–4.62)** | | NR |
|  |  |  |  |  |  |  | Mortality | **3.01 (1.44–6.28)** | | NR |
| Kim[59] | 2020 | South Korea | 9148 | 9148 | No malignant neoplasm of prostate (n = 9123) | Malignant neoplasm of prostate (n = 25) | Mortality | **2.88 (1.01–8.22)** | | NR |
| Kim[60] | 2021 | South Korea | 75 527 | 1911 | No malignancy (n = 136) | Malignancy (n = 1775) | Severity grade 1 | 1.17 (0.79–1.73) | | .4253 |
|  |  |  |  |  |  |  | Severity grade 2 | 1.36 (0.82–2.24) | | .2299 |
|  |  |  |  |  |  |  | Mortality | 1.45 (0.85–2.44) | | .17 |
| Kim[106] | 2022 | South Korea | 129 120 | 8070 | No malignancy (n = NR) | Malignancy (n = 536) | Severe COVID-19 | **1.84 (1.15–2.94)** | | NR |
| Kim[61] | 2022 | South Korea | 5624 | 5624 | Malignancy (n = 145) | No malignancy (n = 5479) | Mortality | **0.20 (0.07–0.55)** | | < .01 |
|  |  |  |  |  |  |  | Clinical Severity score | **-0.85(0.27–0.69)** | | < .01 |
| Kim[62] | 2020 | South Korea | 2959 | 2959 | No cancer (n = 2864) | Cancer (n = 95) | Severe COVID-19 | 2.67 (0.75–9.54) | | NR |
| Lee[64] | 2020 | South Korea | 5061 | 5061 | No malignancy (n = 5022) | Malignancy (n = 39) | Mortality | **4.33 (1.53–12.2)** | | .006 |
|  |  |  |  | 98^e^ | No cancer (n = 87) | Cancer (n = 11) | Mortality | 2.54 (0.66–9.71) | | .228 |
|  |  |  |  |  |  |  | HFNC or MV | 3.55 (0.98–12.8) | | .071 |
| Lee[66] | 2020 | South Korea | 4742 | 4742 | No malignancy (n = 4458) | Malignancy (n = 284) | ICU admission | 1.46 (0.77–2.79) | | NR |
|  |  |  |  | 80^f^ | No malignancy (n = 67) | Malignancy (n = 13) | Ventilator usage | 0.94 (0.14–6.41) | | NR |
| Lee[68] | 2020 | South Korea | 7272 | 7272 | No malignancy (n = 6989) | Malignancy (n = 283) | Respiratory failure | 1.18 (0.60–2.29) | | .621 |
|  |  |  |  |  |  |  | Mortality | 1.01 (0.59–1.72) | | .965 |
| Lee[70] | 2020 | South Korea | 7339 | 7339 | No malignancy (n = 7177) | Malignancies (n = 162) | Severe COVID-19 | 1.26 (0.83–1.91) | | .271 |
|  |  |  |  |  |  |  | Mortality | 1.04 (0.56–1.93) | | .895 |
| Moon[74] | 2021 | South Korea | 5626 | 4426 | No malignancy (n = 4319) | Malignancy (n = 107) | Mortality | **3.98 (1.45–11)** | | .007 |
|  |  |  |  |  |  |  | 30-day and 60-day survival | **2.27 (1.10–4.69)** | | .027 |
| Moon[75] | 2020 | South Korea | 352 | 352 | No malignancy (n = 337) | Malignancy (n = 15) | Mortality | **8.79 (1.44–53.8)** | | .019 |
| Oh[76] | 2022 | South Korea | 5077 | 2106 | No malignancy (n = 2057) | Malignancy (n = 49) | Clinical severity | **2.69 (1.27–5.69)** | | .01 |
|  |  |  |  | 2971 | No malignancy (n = 2880) | Malignancy (n = 91) | Clinical severity | 0.86 (0.25–2.90) | | NR |
| Oh[77] | 2021 | South Korea | 122 040 | 7780 | No lung cancer (n = NR) | Lung cancer (n = NR) | Mortality | 1.82 (0.80–4.14) | | .154 |
|  |  |  |  |  | No malignancy (n = NR) | Malignancy (n = NR) |  | 1.07 (0.78–1.46) | | .694 |
|  |  |  |  |  | No metastatic solid tumor  (n = NR) | Metastatic solid tumor  (n = NR) |  | 1.37 (0.85–2.19) | | .192 |
| Oh[78] | 2021 | South Korea | 122 040 | 7669 | No metastatic solid tumor  (n = 5328) | Metastatic solid tumor  (n = 2341) | Mortality | 1.02 (0.73–1.42) | | .905 |
|  |  |  |  |  | No malignancy (n = 7337) | Malignancy (n = 332) | Mortality | **1.96 (1.26–3.05)** | | .003 |
| Park[80] | 2020 | Korea | 2269 | 2269 | No malignancy (n = 2181) | Malignancy (n = 88) | Mortality | 2.12 (0.94–4.76) | | .067 |
| Shin[84] | 2021 | South Korea | 5571 | 5570 | No malignancy (n = 5427) | Malignancy (n = 143) | Severe COVID-19 | 1.17 (0.66–2.06) | | .59 |
|  |  |  |  |  |  |  | Mortality | **3.38 (1.59–7.17)** | | < .01 |
| Song[85] | 2021 | South Korea | 5621 | 5621 | No malignancy (n = 5478) | Malignancy (n = 143) | Mortality^g^ | **4.13 (1.26–12.6)** | | .015 |
|  |  |  |  |  |  |  | Fatal adverse outcome^h^ | **3.16 (1–9.23)** | | .042 |
|  |  |  |  |  |  |  | Mortality^g^ | **3.05 (1.49–6.25)** | | .002 |
|  |  |  |  |  |  |  | Fatal adverse outcome^g,h^ | **2.67 (1.35–5.31)** | | .005 |
| Yang[117] | 2021 | South Korea | 8070 | 8070 | No lung cancer(n = 8033) | Lung cancer (n = 37) | Severe COVID-19 | **2.24 (1.08–4.74)** | | NR |

Abbreviations: ECMO, extracorporeal membrane oxygenation; HFNC, high-flow nasal cannula; ICU, intensive care unit; IMV, invasive mechanical ventilation; MV, mechanical ventilation; NR, not reported.

Neoplasms 1: Malignant neoplasm of larynx; malignant neoplasm of trachea, bronchus and lung; other malignant neoplasms of respiratory and intrathoracic organs; Neoplasms 2: Malignant neoplasm of cervix uteri; malignant neoplasm of other and unspeciﬁed parts of uterus; other malignant neoplasms of female genital organs; malignant neoplasm of prostate; other malignant neoplasms of male genital organs; malignant neoplasm of bladder; other malignant neoplasms of urinary tract; Neoplasms 3: Malignant neoplasm of other, ill-deﬁned, secondary, unspeciﬁed and multiple sites; Neoplasms 4: Hodgkin’s disease; Non-Hodgkin’s lymphoma; leukemia; other malignant neoplasms of lymphoid, hematopoietic and related tissue.

^a^Residential aged care facility residents. ^b^Hospitalization or death. ^c^ICU admission or death. ^d^Aged ≥50 years. ^e^ICU population. ^f^Conditional logistic regression. ^g^Cox proportional hazard. ^h^IMV, multiorgan failure, ECMO, and death.

### Supplementary Table 12. Studies assessing immunosuppression

| **First author** | **Year** | **Country** |  | **Cohort size** | **Reference (sample size)** | **Comparator**  **(sample size)** | **Outcome** | **OR/HR (95% CI)** | ***P*-value** |
| --- | --- | --- | --- | --- | --- | --- | --- | --- | --- |
| Liu[4] | 2021 | Australia | 4054 | 4054 | No immunosuppressive condition (n = 3925) | Immunosuppressive condition (n = 129) | Severe COVID-19^a^ | **1.66 (1.19–2.33)** | NR |
|  |  |  |  |  |  |  | Very severe COVID-19^b^ | **2.20 (1.35–3.57)** | NR |
| Nishimura[24] | 2023 | Japan | 30 130 | 1097 | No immunodeficiency  (n = 381) | Immunodeficiency (n = 102) | Hospitalization | **2.6 (1.38–4.88)** | .003 |
|  |  |  |  |  | No immunodeficiency  (n = 370) | Immunodeficiency (n = 91) | Oxygen administration | 0.51 (0.25–1.06) | .072 |
| Okauchi[26] | 2021 | Japan | 84 | 84 | No lymphocytopenia (≥1000/μL) (n = NR) | Lymphocytopenia  (< 1000/μL) (n = NR) | Oxygen supplementation | **29.4 (4.08–212)** | < .001 |
| Sato[29] | 2022 | Japan | 500 |  | No immunosuppressed status (n = 416) | Immunosuppressed status  (n = 28) | Mortality or ECMO or IMV or MV or ICU admission | 1.54 (0.53–4.48) | .42 |
| Baek[116] | 2021 | South Korea | 6435 | 5186 | Non-immunocompromised (n = 5188) | Immunocompromised  (n = 5186) (IPTW) | Mortality | **2.09 (1.62–2.68)** | < .001 |
|  |  |  |  |  |  |  | Conventional oxygen therapy | **1.19 (1.05–1.35)** | .007 |
|  |  |  |  |  |  |  | HFNC | **1.28 (1.00–1.65)** | .055 |
|  |  |  |  |  |  |  | IMV | **1.06 (1.05–1.07)** | .018 |
|  |  |  |  |  |  |  | ECMO | **2.17 (1.02–4.63)** | .045 |
|  |  |  |  |  |  |  | Vasopressor use | 1.04 (0.79–1.38) | .773 |
|  |  |  |  |  |  |  | Renal replacement therapy | 1.26 (0.61–2.63) | .534 |
|  |  |  |  |  |  |  | Acute heart failure | **1.39 (1.18–1.64)** | < .001 |
| Choi[49] | 2021 | South Korea | 7590 | 7590 | No AIDS (n = 7583) | AIDS (n = 7) | Mortality | 7.08 (0.46–109) | .160 |
|  |  |  |  |  |  |  | ICU admission | 0 (0–1000) | .983 |
| Ji[104] | 2020 | South Korea | 219 961 | 7341 | No bone marrow dysfunction (n = 7317) | Bone marrow dysfunction  (n = 24) | Severe COVID-19 | 1.13 (0.41–3.12) | NR |
|  |  |  |  |  | No immune deficiency, HIV infection (n = 7337) | Immune deficiency, HIV infection (n = 4) |  | 2.34 (0.19–28.8) | NR |
|  |  |  |  |  |  |  |  | 2.34 (0.19–28.8) | NR |
| Lee[63] | 2023 | South Korea | 467 | 467 | No immunocompromised condition (n = 365) | Moderately to severely immunocompromised condition (n = 102) | Mortality | **18.4 (5.80–58.3**) | < .001 |
| Lee[70] | 2020 | South Korea | 7339 | 7339 | No HIV infection (n = 7335) | HIV infection (n = 4) | Severe COVID-19 | 5.92 (0.43–81.2) | .183 |
|  |  |  |  |  |  |  | Mortality | **107 (6.38– > 999)** | .001 |
| Oh[77] | 2021 | South Korea | 122 040 | 7780 | No AIDS/HIV (n = NR) | AIDS/HIV (n = NR) | Mortality | 1.43 (0.11–19.4) | .788 |
| Oh[78] | 2021 | South Korea | 114 371 | 7669 | No AIDS/HIV (n = 7659) | AIDS/HIV (n = 10) | Mortality | 1.19 (0.09–15.6) | .893 |

Abbreviations: ECMO, extracorporeal membrane oxygenation; HFNC, high-flow nasal cannula; ICU, intensive care unit; IMV, invasive mechanical ventilation; MV, mechanical ventilation; NR, not reported.

^a^Hospitalization or death. ^b^ICU or death.

### Supplementary Table 13. Studies assessing autoimmune disorders (rheumatic and connective tissue)

| **First author** | **Year** | **Country** | **Total** | **Cohort size** | **Reference  (sample size)** | **Comparator  (sample size)** | **Outcome** | **OR/HR (95%CI)** | ***P*-value** |
| --- | --- | --- | --- | --- | --- | --- | --- | --- | --- |
| Kurahara[102] | 2021 | Japan | 404 | 404 | No connective tissue disease (n = 392) | Connective tissue disease  (n = 12) | Acute respiratory failure requiring oxygenation | 0.97 (0.31–3.12) | .965 |
| Miyashita[20] | 2022 | Japan | 937 758 | 937 758 | No rheumatic disease  (n = 881 819) | Rheumatic disease  (n = 13 889) | Severe COVID-19 | **1.19 (1.12–1.26)** | < .001 |
|  |  |  |  |  |  |  | Mortality | **1.20 (1.11–1.30)** | < .001 |
| Choi[47] | 2021 | South Korea | 7590 | 7590 | No rheumatologic disease  (n = NR) | Rheumatologic disease  (n = NR) | Mortality | 1.35 (0.54–3.38) | .519 |
| Choi[49] | 2021 | South Korea | 7590 | 7590 | No rheumatologic disease  (n = 7029) | Rheumatologic disease  (n = 561) | Mortality | 1.14 (0.7–1.87) | .592 |
|  |  |  |  |  |  |  | ICU admission | 1.23 (0.79–1.91) | .362 |
| Huh[52] | 2021 | South Korea | 44 046 | 2805 | No RA, SLE, GCA, JIA  (n = 2760) | RA, SLE, GCA, JIA (n = 135) | Severe COVID-19 | 0.72 (0.47–1.10) | .13 |
|  |  |  |  |  | No connective tissue disease (n = 2794) | Connective tissue disease (n = 11) | Severe COVID-19 | 1.76 (0.50–6.21) | .38 |
| Ji[104] | 2020 | South Korea | 219 961 | 7341 | No rheumatic arthritis  (n = 7155) | Rheumatic arthritis (n = 186) | Severe COVID-19 | 0.76 (0.48–1.20) | NR |
|  |  |  |  |  | No systemic connective tissue disease (n = 7302) | Systemic connective tissue disease (n = 39) | Severe COVID-19 | 1.19 (0.44–3.18) | NR |
| Kim[59] | 2020 | South Korea | 9148 | 9148 | No arthrosis (n = 7248) | Arthrosis (n = 1900) | Mortality | **1.59 (1.05–2.43**) | NR |
| Kim[106] | 2022 | South Korea | 129 120 | 8070 | No autoimmune disease  (n = NR) | Autoimmune disease (n = 358) | Severe COVID-19 | 2.45 (0.92–6.52) | NR |
| Kim[62] | 2020 | South Korea | 2959 | 2959 | No rheumatoid disease  (n = 2934) | Rheumatoid disease (n = 25) | Severe COVID-19 | 1.46 (0.07–31.7) | NR |
| Oh[76] | 2022 | South Korea | 5077 | 2106 | No rheumatic/ autoimmune disease (n = 2094) | Rheumatic/ autoimmune disease (n = 12) | Clinical severity | **6.69 (1.60–28.0)** | .01 |
| Oh[77] | 2021 | South Korea | 122 040 | 7780 | No rheumatic disease  (n = NR) | Rheumatic disease (n = NR) | Mortality | 0.58 (0.30–1.12) | .107 |
| Oh[78] | 2021 | South Korea | 122 040 | 7669 | No rheumatic disease  (n = 7437) | Rheumatic disease (n = 232) | Mortality | 0.39 (0.18–0.84) | .016 |
| Shin[84] | 2021 | South Korea | 5571 | 5571 | No rheumatic disease/autoimmune disorder (n = 4204) | Rheumatic disease/ autoimmune disorder (n = 38) | Severe COVID-19 | 1.63 (0.64–4.17) | .31 |
|  |  |  |  |  |  |  | Mortality | 1.55 (0.18–13.2) | .69 |
| Shin[118] | 2021 | South Korea | 133 609 | 31 905 | No autoimmune inflammatory rheumatic disease (n = 23 683) | Autoimmune inflammatory rheumatic disease (n = 8297) | Severe COVID-19 | **1.26 (1.02–1.59)** | .041 |
|  |  |  |  |  |  |  | Mortality | **1.69 (1.01–2.84)** | .046 |
|  |  |  |  | 27 933 | No inflammatory arthritis  (n = 20 834) | Inflammatory arthritis  (n = 7099) | Severe COVID-19 | **1.27 (1.01–1.63)** | .049 |
|  |  |  |  |  |  |  | Mortality | **1.81 (1.02–3.18)** | .04 |
|  |  |  |  |  | No connective tissue disease (n = 5541) | Connective tissue disease  (n = 1896) | Severe COVID-19 | **1.71 (1.06–2.71)** | .025 |
|  |  |  |  |  |  |  | Mortality | 1.87 (0.71–4.85) | .2 |

Abbreviations: GCA, giant cell arteritis; ICU, intensive care unit; JIA, juvenile inflammatory arthritis; NR, not reported; RA, rheumatoid arthritis; SLE, systemic lupus erythematosus.

### Supplementary Table 14. Studies assessing neurological conditions

| **First author** | **Year** | **Country** | **Total** | **Cohort size** | **Reference  (sample size)** | **Comparator  (sample size)** | **Outcomes** | **OR/HR** | ***P*-value** |
| --- | --- | --- | --- | --- | --- | --- | --- | --- | --- |
| Ellis[3] | 2023 | Australia | 1071^a^ | 1071 | No impairment (n = 257) | Mild impairment (n = 252) | Hospitalization | 1.1 (0.7–1.9) | NR |
|  |  |  |  |  |  |  | Mortality | 0.8 (0.4–1.6) | NR |
|  |  |  |  |  |  | Diagnosed dementia  (n = 420) | Hospitalization | **1.9 (1.2–3.0)** | NR |
|  |  |  |  |  |  |  | Mortality | **2.2 (1.3–3.7)** | NR |
| Fan[9] | 2021 | Hong Kong | 3164 | 3164 | No traumatic brain injury related disorders (n = NR) | Traumatic brain injury related disorders (n = NR) | Mortality | **1.86 (1.02–3.41)** | .043 |
| Yip[12] | 2021 | Hong Kong | 5639 | 5639 | No nervous system disease (n = 5375) | Nervous system disease  (n = 264) | Mortality | **2.13 (1.45–3.13)** | < .001 |
| Zhou[13] | 2023 | Hong Kong | 6089 | 6089 | No dementia and Alzheimer (n = 6064) | Dementia and Alzheimer  (n = 25) | Severe COVID-19 | **6.37 (2.36–17.2)** | .0003 |
| Kurahara[102] | 2021 | Japan | 404 | 404 | No dementia (n = 325) | Dementia (n = 79) | Acute respiratory failure requiring oxygen | 0.37 (0.21–0.64) | < .001 |
| Miyashita[20] | 2022 | Japan | 937 758 | 937 758 | No dementia (n = 909 935) | Dementia (n = 27 823) | Severe COVID-19 | **1.27 (1.23–1.32)** | < .001 |
|  |  |  |  |  |  |  | Mortality | **1.58 (1.52–1.65)** | < .001 |
|  |  |  |  |  | No hemiplegia or paraplegia (n = 825 656) | Hemiplegia or paraplegia  (n = 3714) | Severe COVID-19 | **1.18 (1.07–1.30)** | .001 |
|  |  |  |  |  |  |  | Mortality | **1.23 (1.10–1.38)** | < .001 |
| Nojiri[91] | 2023 | Japan | 11 440 | 11 440 | No dementia (n = 11 360) | Dementia (n = 80) | Severe COVID-19 | 0.96 (0.57–1.58) | .9 |
|  |  |  |  |  | No Parkinson disease  (n = 11 410) | Parkinson disease (n = 30) |  | 1.08 (0.48–2.34) | .8 |
|  |  |  |  | 5980^b^ | No dementia (n = 5900) | Dementia (n = 80) | Mortality | 0.98 (0.39–2.17) | > .9 |
|  |  |  |  |  | No Parkinson disease  (n = 5950) | Parkinson disease (n = 30) |  | **3.57 (1.08–10.2)** | .024 |
| Anlacan[34] | 2023 | Philippines | 10 881 | 10 881 | No dementia (n = NR) | Dementia (n = 38) | Mortality | **1.86 (1.21–2.87)** | .005 |
|  |  |  |  |  |  |  | Respiratory failure | **3.14 (1.92–5.15)** | < .001 |
|  |  |  |  |  |  |  | ICU admission | **3.46 (2.18–5.51)** | < .001 |
| Bae[41] | 2021 | South Korea | 1760 | 1232 | No dementia (n = 1112) | Dementia (n = 120) | Mortality | **3.06 (2.02–4.63)** | < .001 |
| Byeon[42] | 2021 | South Korea | 12 646 |  | No mental and behavioral disorders 1 (n = 11 876) | Mental and behavioral disorders 1 (n = 467) | Mortality | **1.38 (1.03–1.84)** | NR |
|  |  |  |  |  | No mental and behavioral disorders 3 (n = 11 367) | Mental and behavioral disorders 3 (n = 976) |  | 1.30 (0.97–1.75) | NR |
|  |  |  |  |  | No diseases of the nervous system 1 (n = 11 799) | Diseases of the nervous system 1 (n = 544) |  | 1.07 (0.79–1.45) | NR |
| Cho[47] | 2021 | South Korea | 7590 |  | No dementia (n = NR) | Dementia (n = NR) | Mortality | **1.91 (1.35–2.70)** | < .001 |
|  |  |  |  |  | No hemiplegia (n = NR) | Hemiplegia (n = NR) | Mortality | 1.50 (0.81–2.76) | .195 |
| Cho[48] | 2021 | South Korea | 5594 | 3729 | No dementia (n = 3359) | Dementia (n = 150) | Overall survival | **3.92 (2.33–6.61)** | < .001 |
| Cho[92] | 2021 | South Korea | 1272 | 1272 | No dementia (n = 1155) | Dementia (n = 117) | Mortality | **5.55 (3.28–9.33)** | NR |
|  |  |  |  |  |  |  | Severe COVID-19 | **4.55 (2.72–7.55)** | NR |
| Choi[49] | 2021 | South Korea | 7590 | 7590 | No dementia (n = 7022) | Dementia (n = 568) | Mortality | **1.6 (1.11–2.31)** | .012 |
|  |  |  |  |  |  |  | ICU admission | 0.77 (0.51–1.16) | .216 |
|  |  |  |  |  | No paralysis (n = 7445) | Paralysis (n = 145) | Mortality | 1.7 (0.98–2.96) | .059 |
|  |  |  |  |  |  |  | ICU admission | 1.1 (0.57–2.15) | .776 |
| Her[51] | 2021 | South Korea | 5628 | 3940 | No dementia (n = 3767) | Dementia (n = 173) | Mortality | **2.67 (1.49–4.78)** | < .001 |
| Hwang[53] | 2020 | South Korea | 340 | 338 | No dementia (n = 289) | Dementia (n = 49) | Severe pneumonia | 1.38 (0.71–2.66) | .340 |
|  |  |  |  |  |  |  | Mortality | 1.43 (0.66–3.13) | .368 |
| Hwang[54] | 2020 | South Korea | 103 | 103 | No Alzheimer's dementia  (n = 92) | Alzheimer's dementia  (n = 11) | Mortality | **7.7 (1.5–39.6)** | .015 |
| Ji[104] | 2020 | South Korea | 219 961 | 7341 | No Parkinsonism and movement disorder  (n = 7076) | Parkinsonism and movement disorder  (n = 265) | Severe COVID-19 | 0.78 (0.55–1.12) | NR |
|  |  |  |  |  | No Alzheimer and degenerative disease  (n = 7134) | Alzheimer and degenerative disease (n = 207) |  | 0.7 (0.49–0.99) | NR |
|  |  |  |  |  | No dementia (n = 6973) | Dementia (n = 368) |  | 1.16 (0.86–1.57) | NR |
|  |  |  |  |  | No Parkinsonism and movement disorder  (n = 7076) | Parkinsonism and movement disorder  (n = 265) |  | 0.78 (0.55–1.12) | NR |
|  |  |  |  |  | No multiple sclerosis  (n = 7335) | Multiple sclerosis (n = 6) |  | 1.79 (0.25–12.9) | NR |
|  |  |  |  |  | No epilepsy (n = 7210) | Epilepsy (n = 131) |  | 1.24 (0.76–2.01) | NR |
|  |  |  |  |  | No neurosis (n = 6410) | Neurosis (n = 931) |  | 1.05 (0.84–1.31) | NR |
|  |  |  |  |  | No mental retardation, development disorder  (n = 7304) | Mental retardation, development disorder  (n = 37) |  | 0.86 (0.27–2.67) | NR |
| Huh[52] | 2021 | South Korea | 44 046 | 2805 | No chronic neurologic disease (n = 2029) | Chronic neurologic disease (n = 776) | Severe COVID-19 | **1.30 (1.06–1.60)** | .01 |
| Kang[57] | 2021 | South Korea | 4141 | 3827 | No dementia (n = 3706) | Dementia (n = 121) | Severe COVID-19 | **2.13 (1.25–3.63)** | NR |
|  |  |  |  |  |  |  | Mortality | **2.50 (1.43–4.36)** | NR |
| Kim[94] | 2020 | South Korea | 9148 | 9148 | No nerve, nerve root and plexus disorders (n = 8432) | Nerve, nerve root and plexus disorders (n = 716) | Mortality | **1.72 (1.13–2.64)** | NR |
| Kim[106] | 2022 | South Korea | 129 120 | 8070 | No dementia (n = NR) | Dementia (n = 442) | Severe COVID-19 | **2.92 (1.91–4.47)** | NR |
|  |  |  |  |  | No neurologic disease  (n = NR) | Neurologic disease (n = 238) | Severe COVID-19 | 0.89 (0.45–1.75) | NR |
| Kim[61] | 2022 | South Korea | 5624 | 5624 | Dementia (n = 224) | No dementia (n = 5400) | Mortality | **0.21 (0.10–0.44)** | < .001 |
|  |  |  |  |  |  |  | Clinical severity score | **-1.30 (0.17–0.43)** | < .001 |
| Kim[62] | 2020 | South Korea | 2959 | 2959 | No dementia (n = 2846) | Dementia (n = 113) | Severe COVID-19 | **3.51 (1.33–9.29)** | NR |
| Lee[119] | 2020 | South Korea | 814 | 781 | Nonmental disorder  (n = 440) | Dementia (n = 93) | Mortality | 1.71 (0.89–3.19) | .1014 |
| Moon[74] | 2021 | South Korea | 5626 | 4426 | No dementia (n = 4305) | Dementia (n = 121) | Mortality | **8.37 (4.25–16.5)** | < .001 |
|  |  |  |  |  |  |  | 30-day and 60-day survival | **4.45 (2.69–7.36)** | < .001 |
| Oh[76] | 2022 | South Korea | 5077 | 2106^c^ | No dementia (n = 2038) | Dementia (n = 68) | Clinical severity | **4.09 (2.14–7.82)** | .001 |
|  |  |  |  | 2971^d^ | No dementia (n = 2820) | Dementia (n = 151) | Clinical severity | **3.08 (1.81–5.23)** | .001 |
| Oh[77] | 2021 | South Korea | 122 040 | 7780 | No dementia (n = NR) | Dementia (n = NR) | Mortality | **1.61 (1.11–2.32)** | .011 |
|  |  |  |  |  | No hemiplegia or paraplegia (n = NR) | Hemiplegia or paraplegia  (n = NR) | Mortality | **1.92 (1.03–3.59)** | .04 |
| Park[80] | 2020 | South Korea | 2269 | 2269 | No chronic neurological disorder (n = 2254) | Chronic neurological disorder (n = 15) | Mortality | **10.8 (2.27–51.5)** | .003 |
| Oh[78] | 2021 | South Korea | 122 040 | 7669 | No dementia (n = 7251) | Dementia (n = 418) | Mortality | **1.87 (1.27–2.73)** | .001 |
|  |  |  |  |  | No hemiplegia or paraplegia (n = 7565) | Hemiplegia or paraplegia  (n = 104) | In-hospital mortality | 1.59 (0.83–3.07) | .163 |
| Seon[82] | 2021 | South Korea | 7713 | 7713^e^ | No dementia (n = 7518) | Dementia (n = 195) | Mortality | **2.12 (1.39–3.22)** | .001 |
| Shin[84] | 2021 | South Korea | 5571 | 5571 | No dementia (n = 5021) | Dementia (n = 224) | Severe COVID-19 | 0.84 (0.50–1.42) | .52 |
|  |  |  |  |  |  |  | Mortality | **2.62 (1.45–4.72)** | < .01 |
| Song[85] | 2021 | South Korea | 5621 | 5621 | No dementia (n = 5397) | Dementia (n = 224) | Mortality^f^ | **11.6 (4.96–27.9)** | < .001 |
|  |  |  |  |  |  |  | Fatal adverse outcome^f,h^ | **7.92 (3.55–17.8)** | < .001 |
|  |  |  |  |  |  |  | Mortality^g^ | **6.38 (3.76–10.8)** | < .001 |
|  |  |  |  |  |  |  | Fatal adverse outcome^g,h^ | **5.41 (3.24–9.02)** | < .001 |
| Wang[86] | 2021 | South Korea | 2800^b^ | 446 | No dementia (n = 233) | Dementia (n = 233) | Mortality | **3.05 (1.80–5.30)** | < .001 |

Abbreviations: ECMO, extracorporeal membrane oxygenation; HFNC, high-flow nasal cannula; ICU, intensive care unit; IMV, invasive mechanical ventilation; MV, mechanical ventilation; NR, not reported.

Mental and behavioral disorders 1: Dementia; Parkinson’s disease; Alzheimer’s disease Mental and behavioral disorders; 2: Nerve, nerve root and plexus disorders; other diseases of the nervous system; 3: Other mental and behavioral disorders; inﬂammatory diseases of the central nervous system; multiple sclerosis; Diseases of the nervous system 1: Epilepsy; transient cerebral ischemic attacks and related syndromes; cerebral palsy and other paralytic syndromes Diseases of the nervous system.

^a^Residential aged care facility residents. ^b^Aged ≥50 years. ^c^Male. ^d^Female. ^e^Patients with mental illness. ^f^Multivariable logistic regression. ^g^Cox regression analysis. ^h^IMV, multiorgan failure, ECMO, and death.

### Supplementary Table 15. Studies assessing mental and behavioural disorders

| **First author** | **Year** | **Country** | **Total** | **Cohort size** | **Reference**  **(sample size)** | **Comparator**  **(sample size)** | **Outcome** | **OR/HR (95% CI)** | ***P*-value** |
| --- | --- | --- | --- | --- | --- | --- | --- | --- | --- |
| Nojiri[91] | 2023 | Japan | 11 440 | 11 440 | No schizophrenia  (n = 11 396) | Schizophrenia (n = 44) | Severe COVID-19 | 1.24 (0.63–2.43) | .5 |
|  |  |  |  |  | No depression anxiety  (n = 11 302) | Depression anxiety (n = 138) |  | 0.85 (0.57–1.25) | .4 |
|  |  |  |  | 5980^a^ | No schizophrenia (n = 5936) | Schizophrenia (n = 44) | Mortality | 0.36 (0.05–1.38) | .2 |
|  |  |  |  |  | No depression anxiety  (n = 5842) | Depression anxiety (n = 138) |  | 1.05 (0.46–2.19) | .9 |
| Byeon[42] | 2021 | South Korea | 12 646 | 12 646 | No mental and behavioral disorders 2 (n = 10 333) | Mental and behavioral disorders 2 (n = 2010) | Mortality | 1.09 (0.84–1.42) | NR |
| Jeon[120] | 2020 | South Korea | 230 565 | 3608 | No mental disorders  (n = 2865) | Schizophrenia, schizotypal and delusional disorders  (n = 162) | Severe COVID-19 | 2.69 (0.62–11.7) | NR |
|  |  |  |  |  |  |  | Mortality | 2.71 (0.11–66.2) | NR |
|  |  |  |  |  |  | Mood disorders (n = 280) | Severe COVID-19 | 1.48 (0.70–3.13) | NR |
|  |  |  |  |  |  |  | Mortality | **3.57 (1.36–9.38)** | NR |
|  |  |  |  |  |  | Mental disorders (n = 743) | Severe COVID-19 | 1.17 (0.76–1.81) | NR |
|  |  |  |  |  |  |  | Mortality | **1.84 (1.07–3.15)** | NR |
| Ji[104] | 2020 | South Korea | 219 961 | 7341 | No mental disorder of substance use (n = 7255) | Mental disorder of substance use (n = 86) | Severe COVID-19 | 0.52 (0.27–0.99) | NR |
|  |  |  |  |  | No schizophrenia (n = 7078) | Schizophrenia (n = 263) |  | 1.27 (0.84–1.92) | NR |
|  |  |  |  |  | No mood disorder (n = 6545) | Mood disorder (n = 796) |  | 1.04 (0.81–1.33) | NR |
|  |  |  |  |  | No personality disorder  (n = 7328) | Personality disorder (n = 13) |  | 1.4 (0.38–5.12) | NR |
| Kim [59] | 2020 | South Korea | 9148 | 9148 | No schizophrenia schizotypal and delusional disorders  (n = 9117) | Schizophrenia schizotypal and delusional disorders  (n = 231) | Mortality | **2.25 (1.06–4.77)** | NR |
| Kim[106] | 2022 | South Korea | 129 120 | 8070 | No psychotic disorder  (n = NR) | Psychotic disorder (n = 2134) | Severe COVID-19 | **1.29 (1.01–1.66)** | NR |
| Lee[119] | 2020 | South Korea | 814^b^ | 781 | No mental disorder (n = 545) | Mental disorder (n = 236) | Mortality | 1.57 (0.95–2.56) | .0733 |
| Lee[121] | 2020 | South Korea | 94 116 | 2640 | No mental illness (n = 1320) | Any mental illness (n = 1320) | Severe COVID-19 | **1.27 (1.01–1.66)** | < .05 |
|  |  |  |  |  |  | Other mental illness  (n = 946) |  | 0.98 (0.70–1.37) | < .05 |
|  |  |  |  |  |  | Severe mental illness  (n = 374) |  | 2.27 (1.50–3.41) | < .05 |
|  |  |  |  |  |  | Any mental illness (n = 1320) | Mortality | 1.38 (1.00–1.95) | < .05 |
|  |  |  |  |  |  |  | ICU admission | 1.18 (0.82–1.70) | NR |
|  |  |  |  |  |  |  | IMV | 1.28 (0.72–2.27) | NR |
| Seon[82] | 2021 | South Korea | 7713^c^ | 7713 | No mental illness (n = 6976) | Mental illness (n = 737) | Mortality | **2.18 (1.57–3.04)** | < .001 |
|  |  |  |  |  | No severe mental illness  (n = 7523) | Severe mental illness  (n = 190) |  | **3.53 (1.82–6.83)** | < .001 |
|  |  |  |  |  | No depression disorder  (n = 7578) | Depression disorder  (n = 135) |  | 1.87 (0.82–4.26) | .136 |
|  |  |  |  |  | No anxiety and OCD  (n = 7610) | Anxiety and OCD (n = 103) |  | 0.18 (0.02–1.34) | .094 |
|  |  |  |  |  | No substance use disorder (n = 7658) | Substance use disorder  (n = 55) |  | 0.74 (0.21–2.66) | .643 |
| Yun[87] | 2020 | South Korea | 7590 | 7363 | No psychiatric disease  (n = 4927) | Psychiatric disease  (n = 2436) | Severe COVID-19 | **1.73 (1.25–2.39)** | < .05 |

Abbreviations: ICU, intensive care unit; IMV, invasive mechanical ventilation; NR, not reported.

Mental and behavioral disorders 2: Schizophrenia; schizotypal and delusional disorders; mood disorders; neurotic, stress-related and somatoform disorders.

^a^Aged ≥50 years. ^b^Aged ≥65 years. ^c^Patients with mental illness.

### Supplementary Table 16. Studies assessing other risk factors

| **First author** | **Year** | **Country** | **Total** | **Cohort size** | **Reference (sample size)** | **Comparator (sample size)** | **Outcome** | **OR/HR (95%CI)** | ***P*-value** |
| --- | --- | --- | --- | --- | --- | --- | --- | --- | --- |
| Zhou[13] | 2023 | Hong Kong | 6089 | 6089 | No gastrointestinal bleeding (n = 5947) | Gastrointestinal bleeding  (n = 142) | Severe COVID-19 | **7.59 (4.87–11.8)** | < .0001 |
| Zhou[14] | 2020 | Hong Kong | 1043 | 1043 | No gastrointestinal disease  (n = 869) | Gastrointestinal diseases  (n = 174) | ICU admission | 1.83 (0.55–6.08) | .3242 |
| Miyashita [20] | 2022 | Japan | 937 758 | 937 758 | No peptic ulcer disease  (n = 923 869) | Peptic ulcer disease  (n = 67 798) | Severe COVID-19 | **1.05 (1.01–1.08)** | .006 |
|  |  |  |  |  |  | Peptic ulcer disease  (n = 67 798) | Mortality | **1.07 (1.03–1.12)** | .001 |
| Shoji[122] | 2022 | Japan | 4006 | 254 | First trimester of pregnancy (n = 66) | Second to third trimester of pregnancy (n = 188) | Moderate-to-severe COVID-19 | **5.3 (1.22–23.1)** | .026 |
| Larrazabal [123] | 2021 | Philippines | 355 | 355 | Not malnourished (n = 100) | Malnourished (n = 255) | Mortality | 1.06 (0.37–3.02) | NR |
|  |  |  |  |  | Nutritional risk - low  (n = 134) | Nutritional risk - moderate  (n = 168) | Mortality | 1.05 (0.47–2.34) | NR |
|  |  |  |  |  | Nutritional risk - low  (n = 134) | Nutritional risk - high (n = 53) | Mortality | 0.63 (0.22–1.76) | NR |
| Byeon[42] | 2021 | South Korea | 12 646 | 12 646 | No diseases of the genitourinary system  (n = 11 846) | Diseases of the genitourinary system (n = 497) | Mortality | **1.71 (1.26–2.32**) | NR |
| Cho[47] | 2021 | South Korea | 7590 | 7590 | No peptic ulcer disease  (n = NR) | Peptic ulcer disease (n = NR) | Mortality | 1.10 (0.74–1.66) | .635 |
| Choi[49] | 2021 | South Korea | 7590 | 7590 | No peptic ulcer disease  (n = 5153) | Peptic ulcer disease (n = 2437) | Mortality | 0.83 (0.6–1.14) | .255 |
|  |  |  |  |  | No peptic ulcer disease  (n = 5153) | Peptic ulcer disease (n = 2437) | ICU admission | 1.03 (0.77–1.39) | .836 |
| Huh[52] | 2021 | South Korea | 44 046 | 2805 | No pancreatitis (n = 2732) | Pancreatitis (n = 73) | Severe COVID-19 | 0.88 (0.51–1.51) | .63 |
| Hwang[53] | 2020 | South Korea | 340^a^ | 338 | No ADL impairment (n = 256) | ADL impairment (n = 82) | Severe pneumonia | **5.33 (2.42–11.7)** | < .001 |
|  |  |  |  | 340 | No ADL impairment (n = 256) | ADL impairment (n = 84) | Mortality | **7.13 (2.93–17.4)** | < .001 |
| Ji[104] | 2020 | South Korea | 219 961 | 7341 | No urolithiasis (n = 7256) | Urolithiasis (n = 85) | Severe COVID-19 | 0.93 (0.51–1.71) | NR |
|  |  |  |  |  | No osteoporosis (n = 6708) | Osteoporosis (n = 633) |  | 0.900 (0.7–1.15) | NR |
|  |  |  |  |  | No thyroid disease (n = 6907) | Thyroid disease (n = 434) |  | 1.12 (0.83–1.5) | NR |
|  |  |  |  |  | No cushing syndrome  (n = 7339) | Cushing syndrome (n = 2) |  | 8.5 (0.41–177) | NR |
|  |  |  |  |  | No non-infectious disease of upper digestive system  (n = 953) | Non-infectious disease of upper digestive system  (n = 6388) |  | 1.06 (0.78–1.44) | NR |
|  |  |  |  |  | No non-infectious disease of lower digestive system  (n = 5328) | Non-infectious disease of lower digestive system  (n = 2013) |  | 0.97 (0.81–1.16) | NR |
|  |  |  |  |  | No pancreatic disease  (n = 7301) | Pancreatic disease (n = 40) |  | 0.26 (0.09–0.75) | NR |
|  |  |  |  |  | No anemia (n = 6836) | Anemia (n = 505) |  | 1.00 (0.76–1.32) | NR |
|  |  |  |  |  | No coagulopathy (n = 7306) | Coagulopathy (n = 35) |  | 0.59 (0.24–1.44) | NR |
|  |  |  |  |  | No nutritional deficiency  (n = 7054) | Nutritional deficiency (n = 287) |  | 1.0 (0.71–1.41) | NR |
| Kim[59] | 2020 | South Korea | 9148 | 9148 | No hemorrhagic conditions and other diseases of blood and bloodforming organs  (n = 9076) | Hemorrhagic conditions and other diseases of blood and bloodforming organs (n = 72) | Mortality | **3.88 (1.52–9.88)** | NR |
|  |  |  |  |  | No other endocrine, nutritional and metabolic disorders (n = 7517) | Other endocrine, nutritional and metabolic disorders  (n = 1631) |  | **1.47 (1.01–2.14)** | NR |
|  |  |  |  |  | No cataract and other disorders of lens (n = 8440) | Cataract and other disorders of lens (n = 708) |  | **1.68 (1.11–2.55)** | NR |
|  |  |  |  |  | No acute laryngitis and tracheitis (n = 7263) | Acute laryngitis and tracheitis (n = 1885) |  | **1.71 (1.15–2.56)** | NR |
|  |  |  |  |  | No chronic disease of tonsils and adenoids (n = 8910) | Chronic disease of tonsils and adenoids (n = 238) |  | **3.26 (1.15–9.24)** | NR |
|  |  |  |  |  | No other diseases of the urinary system (n = 8248) | Other diseases of the urinary system (n = 900) |  | **1.55 (1.04–2.32)** | NR |
|  |  |  |  |  | No fracture of neck, thorax or pelvis (n = 8684) | Fracture of neck, thorax or pelvis (n = 464) |  | **1.66 (1.07–2.59)** | NR |
| Kim[106] | 2022 | South Korea | Controls: 121 050 Cases: 8070 | 8070 | No gastrointestinal disease  (n = NR) | Gastrointestinal disease  (n = 3266) | Severe COVID-19 | 1.00 (0.75–1.33) | NR |
| Lee[70] | 2020 | South Korea | 7339 | 7339 | No tuberculosis (n = 7311) | Tuberculosis (n = 28) | Severe COVID-19 | 2.23 (0.85–5.84) | .103 |
|  |  |  |  |  |  |  | Mortality | 2.34 (0.61–8.94) | .216 |
| Lee[72] | 2022 | South Korea | 129 128 | 8080 | No tuberculosis (n = 8067) | Tuberculosis (n = 13) | ICU admission | **4.28 (1.27–14.4)** | .0188 |
|  |  |  |  |  |  |  | Mortality | 2.59 (0.42–16.1) | .3086 |
| Oh[77] | 2021 | South Korea | 122 040 | 7780 | No tuberculosis of lung  (n = NR) | Tuberculosis of lung (n = NR) | Mortality | 1.65 (0.48–5.64) | .423 |
|  |  |  |  |  | No peptic ulcer disease  (n = NR) | Peptic ulcer disease (n = NR) |  | 1.04 (0.73–1.49) | .818 |
| Oh[78] | 2021 | South Korea | 122 040 | 7669 | No peptic ulcer disease  (n = 6655) | Peptic ulcer disease (n = 1014) | Mortality | 1.10 (0.76–1.60) | .62 |
|  |  |  |  |  | No disability (n = 7061) | Mild to moderate disability  (n = 37) |  | 0.87 (0.55–1.38) | .557 |
|  |  |  |  |  | No disability (n = 7061) | Severe disability (n = 291) |  | **2.82 (1.68–4.73)** | < .001 |
| Yang[111] | 2021 | South Korea | 7340 | 7340 | No allergic rhinitis (n = 2176) | Allergic rhinitis (n = 2176) | Severe COVID-19 | **1.27 (1.00–1.64)** | NR |
|  |  |  |  |  | No current allergic rhinitis  (n = 2045) | Current allergic rhinitis  (n = 2054) |  | **1.40 (1.01–1.93)** | NR |
|  |  |  |  |  | No atopic dermatitis  (n = 154) | Atopic dermatitis (n = 154) |  | 0.72 (0.18–2.90) | NR |

Abbreviations: ADL, activities of daily living; ICU, intensive care unit; NR, not reported.

Diseases of the genitourinary system: Acute and rapidly progressive nephritic syndromes; other glomerular diseases; renal tubulointerstitial diseases; renal failure.

^a^Aged ≥65 years.

### Supplementary Table 17. Search terms used in literature search

| **Search engine** | **Search string used** |
| --- | --- |
| Pubmed | Advanced search:  (((("2019/12/01"[Date - Publication]: "3000"[Date - Publication])) AND (COVID-19[MeSH Major Topic])) AND (COVID-19[Title])) AND (Risk [Text Word])) AND (<country/region>[Text Word])  **Apply the following filters**: Humans, English  Where further refinement was required, the following terms were added as required in the following order:   - (<country/region>[Title/Abstract]) rather than [Text Word] eg, (Japan [Title/Abstract]) - (Hospitalization [Text Word]) - (Mortality/death [Text Word]) - (ICU [Text Word]) - (Clinical outcomes [Text Word]) - (Comorbidities [Text Word]) - (At-risk populations [Text Word]) - (<risk factor> [Text Word]) |
| Medline | **Initial search:**  “Risk, severe, covid*, <country/region>”  *Note*: Using ‘*’ will return a search of all words containing the term, eg, covid returns covid, covid-19 and COVID19  Where further refinement was required, the following terms were added as required in the following order:   - Hospitalization - ICU - Mortality/death - Outcomes - Comorbidities - At-risk populations - <risk factor> |
| BioRxiv and Medrxiv | **Using advanced search:**   - Date posted between: “01/12/19 – “ - Include articles from bioRxiv and medRxiv - Full text or Abstract or Title: “Risk AND COVID AND <country/region>   Where further refinement was required, the following terms were added as required in the following order:   - Mortality/hospitalization/ICU/ventilation - COVID-19” (match whole all) - <risk factor> |
| Scopus | **Using advanced search:**  TITLE-ABS-KEY (risk AND severe AND covid) AND (LIMIT-TO (AFFILCOUNTRY, "<country/region>"))  **Filters applied:** Country/region, English  Where further refinement was required, the following terms were added as required in the following order:   - Hospitalization - ICU - Mortality/death - Outcomes - Comorbidities - At-risk populations - <risk factor> |

### Supplementary Table 18. List of search terms and inclusion and exclusion criteria

| **Category** | **Search terms** |
| --- | --- |
| Country/Region | - Australia - Hong Kong & Macau - Japan - New Zealand - Philippines - Singapore - South Korea - Taiwan - Thailand - Vietnam - South-East Asia - Asia - Asia-Pacific - SEA - APAC |
| Potential risk factors | - Age - Elderly - Frailty - Immunocompromise/immunosuppression/ immunosuppressed/immunodeficient/immunodeficiencies   - HIV, AIDS   - Autoimmune/autoinflammatory   - Cancer/neoplasm/malignancy   - Corticosteroids, immunosuppressants/immunosuppressive (medications/therapies)   - Chemotherapy, radiotherapy   - Rheumatism/rheumatic disorder - Obesity, BMI - Cardiovascular/cardiac/heart   - Atrial fibrillation   - Heart failure   - Coronary artery/atherosclerotic   - Hypertension   - Hemorrhagic conditions, bleeding disorders     - Anemia - Cerebrovascular   - Stroke - Cystic fibrosis - Disability/disabilities   - Cerebral palsy   - Down syndrome - Diabetes - Neurologic/neurological/neurocognitive/cognitive   o Alzheimer   - - Dementia - Respiratory/lung/pulmonary   - Asthma   - Bronchiectasis   - COPD   - Interstitial lung disease - Kidney/renal   - Dialysis - Liver   - Cirrhosis   - Hepatitis - Sex - Arthritis - Osteoporosis - Pregnancy - Transplant - Tuberculosis - Vascular disease - Vulnerable - Aged care/nursing home - Race/ethnicity   - Indigenous |
| Key inclusion criteria | - English language - Research articles - Editorials - Reviews - Position statements - Case studies - Risk factor(s) of interest in title and/or abstract |
| Key exclusion criteria | - Article did not discuss risk factor(s) of interest in full text - Contained pooled data where country or region-level data could not be isolated |

Abbreviations: APAC, Asia Pacific; BMI, body mass index; COPD, chronic obstructive pulmonary disease; SEA, South-East Asia.

### Supplementary Table 19. Summary of adult COVID-19 recommendations in select APAC countries

| **Country** | **Age group (years)** | **Time interval** | **Example medical conditions** | **Other** |
| --- | --- | --- | --- | --- |
| Australia [124, 125] | **≥75** | 6 months | *Conditions for which COVID-19 vaccination can be considered (not exhaustive): **Immunocompromise** because of disease or treatment; **cardiac disease** (eg, HF, CAD); **chronic respiratory condition** (eg, severe asthma, COPD); **chronic neurological condition** (eg, seizure disorder, spinal cord injury, neuromuscular disorder); **chronic metabolic condition** (including diabetes); **CKD stages 4–5**; **haematological disorder**; **chronic liver disease** (eg, cirrhosis, NAFLD); **chromosomal abnormality**; **obesity** (BMI ≥30) |  |
|  | **65–74**  **18–64** with severe immunocompromise | Recommended every 12 months or consider* every 6 months |  |  |
|  | **18–64** without severe immunocompromise | Consider* every 12 months |  |  |
| Hong Kong [126, 127] | **≥50**  **18–49** with underlying comorbidities | >6 months since last dose | **Chronic cardiovascular** (except hypertension without complications), **lung diseases**; **metabolic or kidney diseases**; **obesity** (BMI ≥30); **chronic neurological conditions** | Health care workers; pregnancy |
| Japan[128] | **≥65**  **60–64** with underlying medical conditions* | Every 12 months | **Impaired heart, kidney, or respiratory function** that severely restricts their daily lives or those with **impaired immune function caused by HIV** and are almost unable to perform daily activities |  |
| New Zealand [129, 130] | **≥65 ≥50** Māori and Pacific individuals  **≥16** with severe immunocompromise; underlying medical condition or disability | >6 months since last dose (strongly recommended) | **Immunocompromise** because of disease or treatment; **chronic lung or airways disease**; **serious heart conditions** (eg, HF, CAD**); poorly controlled hypertension**; **chronic neurological or neuromuscular disease**; **diabetes**; **CKD**; **severe liver disease**; **severe haematological disorders**; **severe mental illness**; **active cancer**; **obesity** (BMI ≥35); **sickle cell disease**; **Down syndrome** | Pregnancy |
|  | **≥30** | >6 months since last dose (recommended) |  |  |
| Singapore [131, 132] | **≥60**  **18–59** who are medically vulnerable | 12 months  (not <5 months) | **Diabetes; heart conditions** (eg, HF, IHD, cardiomyopathy); **chronic lung conditions** (including severe asthma); **chronic liver conditions** (including cirrhosis); **chronic kidney conditions** (including dialysis); **chronic neurologic conditions** (including stroke); **cancer** on active treatment; **blood conditions** (eg, thalassemia; sickle cell anemia); **immunodeficiencies** (including HIV infection**); obesity** (BMI ≥30); **genetic or metabolic conditions** (including Down syndrome; CF); **noncancer immunosuppressive treatment** | Health care workers; household members and caregivers of individuals who are medically vulnerable; residents of aged care facilities |
| South Korea [133] | **≥65**  **≥18** with immunocompromise | 12 months  (not <3 months) | **Immunocompromise** because of disease or treatment |  |
| Taiwan[134] | **≥65**  **55–64** Indigenous individuals  **≥18** with immunocompromise/ immunoinsufficiency | >3 months since last dose | **Immunocompromise** because of disease or treatment |  |
| Vietnam[135] | **≥50**  **18–49** with underlying medical conditions | >6 months since last dose | *Not available* | Health care workers, pregnant women |

Abbreviations: APAC, Asia Pacific; BMI, body mass index; CAD, coronary artery disease; CF, cystic fibrosis; CKD, chronic kidney disease; COPD, chronic obstructive pulmonary disease; HF, heart failure; IHD, ischemic heart disease; NAFLD, non-alcoholic fatty liver disease.

Note: there are currently no recommendations for additional vaccine doses in the Philippines in 2024.

### Supplementary Table 20. Immunocompromise examples/definitions included in recommendations in select APAC countries

| **Country** |  |
| --- | --- |
| Australia[124, 125] | Immunocompromise because of disease or treatment, asplenia or splenic dysfunction, HIV infection, malignancy, solid organ transplant, haematopoietic stem cell transplant |
| Hong Kong[126, 127] | **Immunocompromised persons include**:  1. Cancer or hematological malignancy on active immunosuppressive treatment now or in the past 12 months 2. Recipients of solid organ transplant or stem cell transplant on immunosuppressive treatment  3. Severe primary immunodeficiency or on chronic dialysis  4. Advanced or untreated HIV disease  5. On active immunosuppressive drugs, or immunosuppressive chemotherapy / radiotherapy in past 6 months |
| Japan[128] | HIV-infected persons with disabilities that interfere with daily life because of immunodeficiency (aged ≥60 years) |
| New Zealand [129, 130] | **Individuals with a weakened immune system.** Some conditions and treatments can weaken a person's immune system, including:   - Having chemotherapy or radiotherapy - Bone marrow or organ transplantation - Some blood cancers - Immune deficiencies including HIV infection - Some immunity weakening medications, such as high-dose corticosteroids and disease-modifying anti-rheumatic drugs that treat inflammatory conditions such as arthritis and inflammatory bowel disease - Long-term hemodialysis or peritoneal dialysis |
| Singapore[131, 132] | - Cancer on active treatment - Blood conditions such as thalassemia and sickle cell anemia - Immunodeficiencies including HIV infection - Persons on noncancer immunosuppressive treatment |
| South Korea[133] | **Immunocompromised patients**   - Patients receiving chemotherapy for a tumor or hematologic malignancy - Patients taking immunosuppressive medications following an organ transplant surgery - Patients who are within 2 years post-hematopoietic stem cell transplantation, or if more than 2 years since transplantation but still receiving immunosuppressive treatment - Patients who have primary (congenital) immunodeficiency (eg, antibody deficiency, DiGeorge syndrome, Wiskott-Aldrich syndrome) - Patients being treated with high doses of corticosteroids or other immunosuppressive medications |
| Taiwan[134] | Patients currently undergoing or who have received immunosuppressive therapy within the past year**,** patients with cancer, recipients of organ transplant/stem cell transplant, patients with moderate/severe congenital immunodeficiency, patients on dialysis, patients with HIV, individuals currently using high-dose immunosuppressive drugs, those who have undergone chemotherapy or radiotherapy in the past 6 months, and others evaluated by a physician to have immunodeficiency or weakened immune systems |

Abbreviation: APAC, Asia Pacific.

### Supplementary Table 21. Risk factors and outcome definitions

| **First author** | **Year** | **Country** | **Risk factor(s) of interest examined** | **Outcomes assessed** | **Definition in paper** |
| --- | --- | --- | --- | --- | --- |
| Bhatia[1] | 2021 | Australia | Age, HF/cardiomyopathy, CKD, COPD, severe valvular disease, diabetes, stroke/TIA, CAD, hypertension, AF/flutter | Mortality | NR (assume all-cause in-hospital mortality in patients with confirmed COVID-19) |
| Davis[2] | 2022 | Australia | Age, diabetes, obesity, CVD, hypertension | Hospitalization | Admission to an in-patient ward or ICU |
| Ellis[3] | 2023 | Australia | Age, sex, cognition, diabetes, IHD, CbVD, AF, HF, CLD, asthma, cancer, hypertension, CKD, BMI | Hospitalization Mortality | Any hospital presentation or admission within 14 days of COVID-19 diagnosis Any death of a person with COVID-19 within 28 days of diagnosis, in the absence of a clear alternative cause (eg, trauma) |
| Liu[4] | 2021 | Australia | Age, sex, immunosuppression, obesity, COPD, IHD, diabetes, CbVD; hypertension, cancer, CKD, asthma | Severe COVID-19 (hospitalization or mortality) Very severe COVID-19 (ICU admission or death) | Severe disease: composite outcome of hospitalization or death from COVID-19 Very severe disease: composite outcome of ICU admission or death from COVID-19 (subset of severe disease group) |
| Muleme[5] | 2023 | Australia | Age, sex | Hospitalization Mortality | Hospital admissions within 14 days of diagnosis or deemed COVID-19–related by reporting hospital Deaths that occurred within 28 days after clearance from COVID-19 isolation or for which COVID was recorded as the primary or antecedent cause of death |
| Shiel[6] | 2021 | Australia | Age | Mortality Severe COVID-19 (ICU admission or death) Incomplete recovery at 30 days | Mortality: NR (assume all-cause mortality in patients with confirmed COVID-19) Severe COVID-19: composite outcome of death or ICU admission Recovery: resolution of all symptoms and self-reported patient estimate of returning to ≥90% their premorbid function |
| Wang[7] | 2023 | Australia | Age, IHD, COPD | Oxygen therapy | Oxygen therapy at any point during hospital admission |
| Chung[8] | 2021 | Hong Kong | Age, sex | Severe COVID-19 (mortality, oxygen therapy [>3L/min], intubation, ECMO or in shock) | Patients with COVID-19 who died or have ever been classiﬁed as critical or serious for 1 or more days during the study period were considered “severe” |
| Fan[9, 10] | 2021 | Hong Kong | Age, neurological disease, renal disease, cancer | Mortality | NR (assume all-cause mortality in patients with confirmed COVID-19) |
| Teoh[10] | 2020 | Hong Kong | Older age, diabetes, AKI | Severe COVID-19 (ICU admission, IMV, and/or death) | Composite endpoint of ICU admission, use of invasive mechanical ventilation, and/or death |
| Wong[11] | 2023 | Hong Kong | Age, sex, diabetes, cancer, CVD | Mortality | NR (assume all-cause in-hospital mortality in patients with confirmed COVID-19) |
| Yip[101] | 2020 | Hong Kong | Diabetes, hypertension | Severe COVID-19 (ICU admission, IMV, and/or death) | Composite endpoint of ICU admission, use of invasive mechanical ventilation, and/or death |
| Yip[12] | 2021 | Hong Kong | HBV exposure, acute liver injury, liver cirrhosis, age, diabetes, cancer, nervous system disease, kidney disease | Mortality | All-cause mortality |
| Zhou[13] | 2023 | Hong Kong | Age, sex, cander, diabetes, hypertension, HF, AF, liver disease, dementia, COPD, myocardial infarction, IHD, vascular disease, stroke/TIA, GI bleeding, obesity | Severe COVID-19 (intubation, ICU admission and 30-day mortality) | Composite endpoint of patients who required intubation, required ICU admission, and/or had 30-day mortality |
| Zhou[14] | 2020 | Hong Kong | Age, sex, CVD, lung disease, diabetes, hypertension, CKD, GI disease | ICU admission | NR |
| Zhou[15] | 2020 | Hong Kong | Age, CVD, diabetes, hypertension, CKD | Mortality | All-cause mortality |
| Fukushima[16] | 2021 | Japan | Age, sex, hypertension, cancer | Critical COVID-19 (ARDS, sepsis, or septic shock) | Composite endpoint of ARDS, sepsis, or septic shock. |
| Fukushima[17] | 2023 | Japan | Prediabetes, undiagnosed diabetes, diagnosed diabetes, age, sex, BMI, hypertension, CVD, CLD, CKD | Critical COVID-19 (high-flow oxygen, IPPV, ECMO, or mortality) | Critical outcomes were defined by using high-flow oxygen, invasive positive-pressure ventilation, ECMO, or death |
| Ito[18, 102] | 2023 | Japan | Age, sex, BMI, hypertension | Hospitalization | Hospitalization (with clinical deterioration) |
| Kurahara[102] | 2021 | Japan | Hypertension, CVD, diabetes, dementia, CKD, underlying pulmonary condition, malignant disease, connective tissue disease | Acute respiratory failure requiring oxygenation |  |
| Lee[96] | 2022 | Japan | BMI | Oxygen therapy ICU admission IMV Mortality Critical COVID-19 (ICU, IMV, or death) | Critical illness: Patients needing treatment in the ICU, requiring invasive mechanical ventilation, or who died |
| Matsushita[19] | 2022 | Japan | Age, sex | COVID severity grade 2/3/4/5: Oxygen therapy, NIPPV, IMV, ECMO, or mortality COVID severity grade 3/4/5: IMV, ECMO, or mortality COVID severity grade 5: Mortality | Grade 2 (patients supported with high-ﬂow oxygen or NIPPV) Grade 3 (patients who required invasive IMV) Grade 4 (ECMO) Grade 5 (death in hospital no matter which treatment was given) |
| Miyashita[20] | 2022 | Japan | Age, sex, diabetes; CLD; CbVD; myocardial infarction; dementia; cancer; COPD; vascular disease; kidney disease; HF; asthma; liver disease, rheumatic disease, peptic ulcer disease, hemiplegia or paraplegia | Severe COVID-19 (use of HFNC, MV, or ECMO, or death) Mortality | Composite endpoint of HFNC therapy, invasive or noninvasive MV, or ECMO |
| Muto[21] | 2021 | Japan | Age, sex, malignancy, diabetes, hemodialysis | Critical COVID-19 (respiratory failure, septic shock and/or multiple organ dysfunction) Severe COVID-19 (SpO2 <94%, PaO2/FiO2 ratio <300 mmHg, RR >30 breaths/min, and/or lung infiltrates >50%) | Critical illness: composite of respiratory failure, septic shock, and/or multiple organ dysfunction Severe illness oxygen saturation <94% in room air at sea level, a ratio of arterial partial pressure of oxygen to fraction of inspired oxygen <300 mmHg, respiratory frequency >30 breaths/min, and/or lung inﬁltrates >50% |
| Ninomiya[22] | 2021 | Japan | Age, overweight, diabetes | Oxygen therapy | Patients who needed oxygen administration because of pneumonia and low oxygen saturation on pulse oximetry (SpO2 ≤ 93% at rest) |
| Nishida[23] | 2022 | Japan | Age, sex | Mortality IMV Composite of mortality or IMV | In-hospital death and invasive mechanical ventilation |
| Nishimura[24] | 2023 | Japan | Age, sex, obesity (BMI ≥ 30kg/m²), diabetes, hypertension, cardiovascular disease, CbVD, CKD, chronic hepatitis or cirrhosis, COPD, asthma, malignant tumor, immunodeficiency | Hospitalization Oxygen therapy | Hospitalization because of COVID-19 Oxygen administration because of COVID-19 |
| Nojiri[91] | 2023 | Japan | Age, sex, cancer, anemia, diabetes, hypertension; arrhythmia, PAD; HF, CLD; ulcers, COPD; dementia; schizophrenia; depression anxiety; CbVD; CVD; MI; asthma; liver disease; rheumatic disease; CKD | Severe COVID-19 (oxygen inhalation plus high-flow therapy and requirement for artificial respiration during hospitalization) Mortality | Severity was defined using a claim history of oxygen inhalation, high-flow therapy, and requirement for artificial respiration during hospitalization  NR (assume all-cause in-hospital mortality in patients with confirmed COVID-19) |
| Numaguchi[25] | 2022 | Japan | Age, sex, BMI, hypertension | Severe COVID-19 (ICU admission or MV) | Admission to an intensive care unit or mechanical ventilator required |
| Ogihara[97] | 2022 | Japan | Obesity | Severe COVID-19 (all-cause mortality or MV or ECMO) | All-cause death, or need MV or ECMO during hospitalization |
| Okauchi[26] | 2021 | Japan | Age, obesity, lymphocytopenia (<1,000/μL/0) | Oxygen therapy | Progression of oxygen requirement in patients with mild to moderate COVID-19. |
| Otoshi[27] | 2021 | Japan | Age, diabetes | Severe COVID-19 (oxygen therapy of >5 ml/min, MV, or ECMO) | Patients who required more than 5 L/min of oxygen administration, patients who could not maintain their respiratory status with oxygen alone and required mechanical ventilation or ECMO |
| Sakamoto[28] | 2022 | Japan | Age, sex, BMI, hypertension, diabetes, heart disease, COPD, liver disease | Hospitalization | Patient who were hospitalized when they required oxygen administration or when they had persistent fever or severe respiratory symptoms |
| Sano[112] | 2022 | Japan | NOAF, preexisting atrial fibrillation | Mortality | In hospital mortality (COVID-related) |
| Sato[29] | 2022 | Japan | Age, sex, sex, hypertension, diabetes, cerebrovascular or cardiovascular disease, COPD, immunosuppressed condition, kidney dysfunction | Composite outcome: mortality, ECMO, IMV/MV, or ICU admission | Primary composite outcome comprised in-hospital death, ECMO, mechanical ventilation (invasive and noninvasive methods), and ICU admission. |
| Shoji[122] | 2022 | Japan | Age, second to third trimester of pregnancy | Moderate to severe COVID-19 (composite outcome: MV, oxygen therapy, SpO2 ≤94%, or tachypnea (RR ≥24 breaths/min)) | Death, need for mechanical ventilation, need for ECMO, or ICU admission was set as the composite outcome for severe COVID-19. Requirement of noninvasive oxygen supports (including nasal cannula, face mask, reservoir mask, high-flow oxygen device, biphasic positive airway pressure, and continuous positive airway pressure) and clinical condition at admission (RR ≥24 breaths/minute, oxygen saturation ≤94% on ambient air, or need of supplemental oxygen administration) |
| Takeyama[30] | 2022 | Japan | Age, hypertension, diabetes, heart disease, cancer, VTE, sex, BMI, lung disease | Mortality | In hospital COVID-19-associated mortality |
| Tanaka[31] | 2021 | Japan | Age, sex | Mortality | NR (assume all-cause mortality in patients with confirmed COVID-19) |
| Yamada[32] | 2021 | Japan | Age, sex, BMI, malignancy, diabetes, CHF, CVD, hypertension | Oxygen therapy | Oxygen administration during hospitalization |
| Jefferies[33] | 2020 | New Zealand | Age, sex, cardiovascular disease (including hypertension), diabetes, chronic lung condition, malignancy | Severe COVID-19 (hospitalization or death) | Defined as hospitalization or death |
| Anlacan[34] | 2023 | Philippines | Age, dementia | Mortality Respiratory failure ICU admission | NR |
| Espiritu[115] | 2022 | Philippines | Malignancy | Severe COVID-19 (severe: dyspnea, respiratory rate >30 breaths/min, hypoxia (SpO2 < 93%), or > 50% lung involvement on imaging within 24–48 h; critical disease: respiratory failure, shock or multiorgan dysfunction)  Mortality Respiratory failure ICU admission | Severe/critical COVID-19 at nadir: severe disease: defined as the presence of dyspnea, respiratory rate > 30 breaths/minute, hypoxia (SpO2 < 93%), or > 50% lung involvement on imaging within 24–48 h; and critical disease: defined as the presence of respiratory failure, shock, or multiorgan dysfunction |
| Espiritu[107] | 2021 | Philippines | Diabetes | Severe COVID-19 (severe: dyspnea, respiratory rate >30 breaths/min, hypoxia (SpO2 < 93%), or > 50% lung involvement on imaging within 24–48 h; critical disease: respiratory failure, shock or multiorgan dysfunction) Mortality Respiratory failure ICU admission | Severe/critical COVID-19 at nadir: severe disease: defined as the presence of dyspnea, respiratory rate > 30 breaths/minute, hypoxia (SpO2 < 93%), or > 50% lung involvement on imaging within 24–48 h; and critical disease: defined as the presence of respiratory failure, shock, or multiorgan dysfunction |
| Espiritu[98] | 2022 | Philippines | BMI | Severe COVID-19 (Severe COVID-19 (severe: dyspnea, respiratory rate >30 breaths/min, hypoxia (SpO2 < 93%), or > 50% lung involvement on imaging within 24–48 h; critical disease: respiratory failure, shock or multiorgan dysfunction) Mortality IMV ICU admission | Severe/critical COVID-19 at nadir: severe disease: defined as the presence of dyspnea, respiratory rate > 30 breaths/minute, hypoxia (SpO2 < 93%), or > 50% lung involvement on imaging within 24–48 h; and critical disease: defined as the presence of respiratory failure, shock, or multiorgan dysfunction |
| Espiritu[103] | 2023 | Philippines | Hypertension | Severe COVID-19 (severe: dyspnea, respiratory rate >30 breaths/min, hypoxia (SpO2 < 93%), or > 50% lung involvement on imaging within 24–48 h; critical disease: respiratory failure, shock, or multiorgan dysfunction) Mortality Respiratory failure ICU admission | Severe/critical COVID-19 at nadir: severe disease: defined as the presence of dyspnea, respiratory rate > 30 breaths/minute, hypoxia (SpO2 < 93%), or > 50% lung involvement on imaging within 24–48 h; and critical disease: defined as the presence of respiratory failure, shock, or multiorgan dysfunction |
| Espiritu[35] | 2021 | Philippines | Age, sex, hypertension, diabetes | Mortality Respiratory failure ICU admission | Severe/critical COVID-19 at nadir |
| Larrazabal[123] | 2021 | Philippines | Malnourished, nutritional risk | Mortality | NR |
| Koh[36] | 2021 | Singapore | Age, sex, diabetes, prediabetes, BMI | Severe COVID-19 (SpO2 ≤93%, RR ≥30, or need for ICU care) ICU admission | Severe COVID‐19: defined by SpO2 ≤93% on room air, RR ≥30, or need for ICU care. |
| Lim[37] | 2021 | Singapore | CCI+Age, CCI+Sex, Frailty Index+Age, Frailty Index+Male | Severe COVID-19 (oxgen therapy, ICU admission, MV, or mortality) | Critical illness refers to the development of any of the following outcomes: needing high flow oxygen, admission to intensive care unit (ICU), noninvasive or invasive mechanical ventilation, or death |
| Ngiam[38] | 2022 | Singapore | Age, underlying medical conditions | Severe COVID-19 (ICU admission, MV, severe AKI, or mortality) | Patients who required intensive care, required mechanical ventilation, had severe acute kidney injury requiring renal replacement therapy, or death |
| Tan[39] | 2022 | Singapore | Age, hypertension | ICU admission and/or mortality Pneumonia | Covid-related admission and death Diagnosed at any point during admission |
| Tee[40] | 2020 | Singapore | Age, diabetes, prediabetes | Pneumonia | NR |
| Bae[41] | 2021 | South Korea | Age, sex, diabetes, HF, CKD, cancer, dementia | Mortality | NR |
| Baek[116] | 2021 | South Korea | Immunocompromised status, Malignancy, solid tumors | Mortality Oxygen therapy HFNC IMV ECMO Vasopressor use Renal replacement therapy Acute HF | NR |
| Byeon[42] | 2021 | South Korea | Age, sex, neoplasms, diabetes, mental disorders, neurologic disorders, CVD, genitourinary disorders | Mortality | NR |
| Chang[43] | 2020 | South Korea | Age, diabetes, chronic lung disease | Mortality | NR |
| Chang[44] | 2020 | South Korea | Age, diabetes, hypertension | Severe COVID-19 (oxygen therapy or MV) | Patients who were initially asymptomatic or with mildly symptomatic COVID-19 who were transferred to a specialized infectious disease hospital because of COVID-19 progression to severe stage. Severe stage defined as high-flow O2 supply or mechanical ventilation required |
| Chang[45] | 2022 | South Korea | Age, sex, hypertension, stroke, CKD, diabetes, CAD, asthma, cancer | Severe COVID-19 (MV, ICU admission, oxygen therapy, or mortality) | Composite of mechanical ventilation, ICU care, high-low oxygen therapy, and death |
| Chang[46] | 2022 | South Korea | Age, sex, hypertension, stroke, CKD, diabetes, CAD, asthma, cancer, AF, HF | Severe COVID-19 (MV, ICU admission, oxygen therapy, or mortality) | Composite of mechanical ventilation, ICU care, high-low oxygen therapy, and death |
| Cho[47] | 2021 | South Korea | Age, sex, hypertension, HF, diabetes, MI, cancer, liver disease, vascular disease, CVD, rheumatologic disease, dementia, CPD, peptic ulcer disease, hemiplegia, renal disease | Mortality | NR |
| Cho[48] | 2021 | South Korea | Age, sex, diabetes, hypertension, dementia, chronic lung disease, CKD, cardiac disease, cancer | Overall survival | The time from COVID-19 diagnosis to death from any cause or up to the date of the last follow-up |
| Cho[92] | 2021 | South Korea | Sex, BMI, diabetes, hypertension, dementia, lung disease, heart disease, CKD, cancer | Mortality Severe COVID-19 (IMV, ECMO, multiorgan failure, or mortality) | Severe COVID-19 defined as the use of invasive mechanical ventilation, the need for ECMO, the development of multi-organ failure, or death |
| Choi[49] | 2021 | South Korea | Age, sex, MI, HF, peripheral vascular disease, CbVD, dementia, chronic pulmonary disease, rheumatologic disease, peptic ulcer disease, liver disease, diabetes, paralysis, renal disease, cancer, HIV/AIDS | Mortality ICU admission | Defined using the associated intervention codes and medical records |
| Chung[50] | 2020 | South Korea | Age, diabetes, hypertension, chronic lung disease, cancer | Severe COVID-19 (ARDS, septic shock, ICU admission, or mortality) | Severe and critical outcome defined as composite outcomes of ARDS, septic shock, ICU care, and 28-day mortality, referring to the classification of the Chinese Center for Disease Control and Prevention. |
| Her[51] | 2021 | South Korea | Age, sex, BMI, diabetes, cancer, dementia | Mortality | NR |
| Her[93] | 2022 | South Korea | Sex | Oxygen therapy Noninvasive ventilation IMV Multi-organ failure/ECMO Mortality | NR |
| Huh[52] | 2021 | South Korea | Age, sex, hypertension, CHD, CLD, asthma/allergic rhinitis, CLD, CKD, cancer, RA/SLE/GCA/JIA, connective tissue disorder, chronic neurologic disease, pancreatitis | Severe COVID-19 (oxygen therapy, HFNC, noninvasive ventilation, MV, ECMO, or mortality) | Requirement of any 1 of the following or death: supplementary oxygen, high-ﬂow nasal cannula, noninvasive ventilation, mechanical ventilation, and ECMO |
| Hwang[53] | 2020 | South Korea | Age, sex, ADL impairment, dementia | Severe pneumonia Mortality | Severe pneumonia deﬁned as fever or suspected respiratory infection, plus 1 of the following: RR >30 breaths/min, severe respiratory distress, or SpO2 ≤ 93% on room air |
| Hwang[54] | 2020 | South Korea | Age, diabetes, chronic lung disease, CVD, Alzheimer dementia, stroke | Mortality | Referred to as nonsurvival (assume all-cause in-hospital mortality in patients with confirmed COVID-19) |
| Jang[55] | 2020 | South Korea | Age, sex, diabetes, hypertension | Severe COVID-19 (ARDS, ICU admission, or mortality) | Defined as a composite outcome of ARDS, ICU care, or death |
| Jang[56] | 2021 | South Korea | Sex, sex | Mortality Oxygen therapy | Need for oxygen support was defined from the maximum disease severity identified during the inpatient period |
| Jeon[113] | 2021 | South Korea | Liver cirrhosis | Severe COVID-19 (oxygen therapy, use of vasopressors, ICU admission, renal replacement therapy, or death) Mortality  Oxygen therapy | Severe complications related to COVID-19 required interventions including oxygen therapy, the use of vasopressors, admission to ICU, continuous renal replacement therapy, and death |
| Jeon[120] | 2020 | South Korea | Schizophrenia, mood disorders, mental disorders | Severe COVID-19 (ICU admission, MV, and ARDS) Mortality | Severe COVID-19 composite of ICU admission, use of mechanical ventilation, and ARDS |
| Ji[104] | 2020 | South Korea | Diabetes, hypertension, lower respiratory disease, CKD/ESRD + 55 other chronic conditions | Severe COVID-19 (oxygen therapy, MV, ECMO, and cardiopulmonary resuscitation) | Confirmed by an RT-PCR test diagnosis, and claims data for oxygen therapy, mechanical ventilator, ECMO, and cardiopulmonary resuscitation |
| Jung[110] | 2021 | South Korea | Asthma, COPD | Severe COVID-19 (ICU admission, IMV, ECMO, and mortality) Mortality | Severe disease/morbidity was defined as admission to the ICU, invasive ventilation, ECMO, and death |
| Kang[57] | 2021 | South Korea | Age, sex, BMI, diabetes, hypertension, CKD, cancer, dementia | Severe COVID-19 (IMV, multi-organ failure, ECMO, and/or mortality) Mortality | Defined as a patient having a “critical illness” if they required more than invasive mechanical ventilation. Critical illnesses included patients requiring invasive mechanical ventilation, those with multi-organ failure, those requiring ECMO therapy, and/or those who died |
| Kang[58] | 2020 | South Korea | Age, sex, hypertension, CKD | Mortality Oxygen therapy IMV ECMO Cardiac arrest MI Acute HF  AKI | Defined using Electronic Data Interchange or ICD codes  AKI was defined as the initiation of hemodialysis, CRRT, or peritoneal dialysis after the diagnosis of COVID-19 in patients not requiring maintenance dialysis |
| Kang[105] | 2021 | South Korea | Hypertension | Severe COVID-19 (ICU admission, MV, and mortality) Hospitalization Oxygen therapy  ICU admission Ventilator care Mortality | The primary study outcome was severe clinical events, a composite of ICU admission, need for ventilator care, and death |
| Kim[94] | 2021 | South Korea | Sex, BMI | Severe COVID-19 (ICU admission, MV, ECMO, or mortality) Mortality | The primary endpoint was defined as the composite of death, admission to ICU, application of mechanical ventilation, or ECMO. |
| Kim[59] | 2020 | South Korea | Age, sex, cancer, hemorrhagic conditions and other diseases of blood and blood-forming organs, diabetes, other endocrine, nutritional and metabolic disorders, schizophrenia schizotypal and delusional disorders, nerve, nerve root and plexus disorders, cataract and other disorders of lens, acute MI, other IHDs, HF, acute laryngitis and tracheitis, influenza, pneumonia, chronic disease of tonsils and adenoids, arthrosis, renal tubulo-interstitial diseases, renal failure, other diseases of the urinary system, fracture of neck, thorax or pelvis, essential hypertension | Mortality | NR |
| Kim[60] | 2021 | South Korea | Age, sex, diabetes, CVD, CbVD, IHD, COPD, malignancy | Severity grade 1 (oxygen therapy, MV, ECMO, or mortality) Severity grade 2 (MV, ECMO, or mortality) Mortality | Severe cases required MV, and critical cases required ECMO. Moderate, severe, critical, and death levels were clustered as Severity grade 1, while severe, critical, and death levels were clustered as Severity grade 2. |
| Kim[106] | 2022 | South Korea | Pulmonary disease, CVD, kidney disease, hepatobiliary disease, gastrointestinal disease, diabetes, hypertension, psychotic disorder, dementia, stroke, neurologic disease, autoimmune disease, malignancy | Severe COVID-19 (tracheostomy, continuous renal replacement therapy, ICU admission, ventilator use, cardiopulmonary resuscitation, and mortality) | Comparing severe + death cases (severe grade was defined as the need for tracheostomy, continuous renal replacement therapy, ICU admission, ventilator use, and cardiopulmonary resuscitation) |
| Kim[61] | 2022 | South Korea | Age, BMI, diabetes, malignancy, dementia | Mortality CSS | Deﬁned as in-hospital mortality CSS stage 3: oxygen therapy, multi-organ damage, and ECMO; Stage 4: death |
| Kim[108] | 2020 | South Korea | Diabetes | Mortality | NR |
| Kim[99] | 2020 | South Korea | BMI, Metabolic health and obesity status | Severe COVID-19 (ICU admission, IMV, ECMO, and mortality) ICU admission IMV or ECMO Mortality | A composite of ICU admissionIVM, ECMO, and death of any cause from the diagnosis of COVID-19 to the end of follow-up |
| Kim[100] | 2020 | South Korea | BMI | Mortality High morbidity during hospitalization (oxygen therapy via nasal cannula/facial mask, MV, multi-organ failure/ECMO, and death) | Maximum morbidity score during hospitalization was categorized as follows: no limitations on activity, limitations on activity but no supplemental oxygen needed, oxygen administered via a nasal cannula, oxygen administered via a facial mask, noninvasive ventilation, invasive ventilation, multiorgan failure/ECMO, and death |
| Kim[62] | 2020 | South Korea | Age, sex, dementia, CKD, malignancy, HF, rheumatoid disease, diabetes, hypertension, BMI, COPD, asthma, CAD, CLD | Severe COVID-19 (noninvasive ventilation, IMV, ECMO, and death) | The severe COVID-19 were classified into 2 groups, such as severe case and nonsevere case. A severe case included no disruption to daily life, hindrance to daily life, and no oxygen required, oxygen treatment via nasal cannula, and oxygen mask. A nonsevere case included noninvasive ventilation, invasive ventilation, ECMO, and death |
| Lee[119] | 2020 | South Korea | Mental disorder, dementia | Mortality | NR |
| Lee[63] | 2023 | South Korea | Age, moderate to severe immunocompromise | Mortality | 90-day mortality after imaging-confirmed pneumonia without any other plausible cause of death |
| Lee[64] | 2020 | South Korea | Age, sex, CVD, CbVD , hypertension, diabetes, pulmonary disease. cancer, CKD | Mortality | NR |
| Lee[65] | 2020 | South Korea | Age, sex | Severe pneumonia (SpO2 ≤93%, PaO2/FiO2 ≤300 mmHg, or MV) | At least one of the following: 1) resting oxygen saturation ≤ 93% in room air, or 2) PaO2/FiO2 ≤300 mmHg or requirement of MV |
| Lee[66] | 2020 | South Korea | Age, sex, malignancy, COPD IHD, hypertension, diabetes | ICU admission MV | Primary objective was to identify risk factors for ICU admission and MV use |
| Lee[67] | 2023 | South Korea | Age, sex, COPD | Mortality | NR |
| Lee[68] | 2020 | South Korea | Age, sex, asthma, hypertension, diabetes, IHD, HF, malignancy | Respiratory failure (MV or ECMO)  Mortality | Cases of mechanical ventilation or ECMO were defined as patients who experienced respiratory failure |
| Lee[69] | 2020 | South Korea | Age, sex, COPD | Respiratory failure (MV or ECMO) Mortality | The use of invasive or noninvasive MV or ECMO |
| Lee[70, 121] | 2020 | South Korea | Age, sex, inﬂuenza, tuberculosis, COPD, pneumonia, asthma, diabetes, CKD, CLD, hypertension, CVD/ CbVD, malignancy, HIV | Severe COVID-19 (oxygen therapy, MV, cardiopulmonary resuscitation, or ECMO) Mortality | The presence of claims for oxygen therapy, MV, cardiopulmonary resuscitation, or ECMO |
| Lee[71, 121] | 2020 | South Korea | Mental illness | Severe COVID-19 (ICU admission, IMV, or mortality) Mortality ICU admission IMV | The severe clinical outcomes of COVID-19 comprised admission to the ICU, invasive ventilation, or death |
| Lee[71, 72] | 2020 | South Korea | Age, liver cirrhosis, COPD, diabetes, CKD | Severe COVID-19 (severe pneumonia, ARDS, sepsis/septic shock, or mortality) Mortality | The degree of severity of COVID-19 (severe vs nonsevere) was defined by the WHO interim guidance for COVID-19. |
| Lee[72] | 2022 | South Korea | Age, sex, tuberculosis | ICU admission Mortality | NR |
| Lim[73] | 2021 | South Korea | Age, COPD | Mortality | NR |
| Moon[74] | 2021 | South Korea | Age, sex, diabetes, malignancy, dementia | Mortality 30-day and 60-day survival | NR |
| Moon[109] | 2020 | South Korea | Diabetes | Hospitalization Oxygen therapy Ventilator support Mortality | NR |
| Moon[75] | 2020 | South Korea | Age, diabetes, malignancy | Mortality | NR |
| Oh[76] | 2022 | South Korea | Age, diabetes, hypertension, HF, chronic cardiac disease, asthma, COPD, CKD, CLD, malignancy, rheumatic/autoimmune disease, dementia, BMI | Severe COVID-19 (chronic renal failure or noninvasive ventilation, high-ﬂow oxygen therapy, IMV, or multi-organ oxygen supply or mortality) | Classified as either severe or dead. ‘Severe’ category included patients with chronic renal failure or those requiring noninvasive ventilation, high-ﬂow oxygen therapy, invasive ventilation, or multi-organ oxygen supply |
| Oh[77] | 2021 | South Korea | Age, sex, chronic respiratory diseases, COPD, asthma, interstitial lung disease, lung cancer, lung disease due to external agent, obstructive sleep apnea, tuberculosis of lung, hypertension, diabetes, PVD, renal disease, rheumatic disease, dementia, peptic ulcer disease, hemiplegia/paraplegia, liver disease, CbVD, HF, MI, malignancy, metastatic solid tumor, AIDS/HIV | Mortality | NR |
| Oh[78] | 2021 | South Korea | Age, sex, PVD, CKD, rheumatic disease, dementia, peptic ulcer disease, diabetes, hemiplegia/paraplegia, liver disease, chronic pulmonary disease, CbVD, HF, MI, solid tumor, malignancy, AIDS/HIV, disability | Mortality | In-hospital mortality |
| Paek[79] | 2020 | South Korea | Age, sex, severe AKI, hypertension, diabetes | Mortality | NR |
| Park[80] | 2020 | South Korea | Age, sex, bronchial asthma, COPD, CKD, malignancy, chronic neurological disorder, CV risk factors/CVD | Mortality | NR |
| Park[81, 95] | 2021 | South Korea | Sex, BMI, CCI score | ICU admission Mortality |  |
| Park[81] | 2020 | South Korea | Age, diabetes, systemic inflammatory response syndrome | Mortality | NR |
| Seon[82] | 2021 | South Korea | Age, sex, CCI score, mental illness, severe mental illness, depression disorder, dementia, anxiety and OCD, substance use disorder | Mortality | COVID-19–related mortality |
| Seong[83] | 2021 | South Korea | Age, diabetes, COPD, CbVD, liver cirrhosis | Mortality | NR |
| Shin[84] | 2021 | South Korea | Age, sex, BMI, diabetes, hypertension, HF, cardiac conduction disease, asthma, COPD, CKD, malignancy, CLD, rheumatic disease/autoimmune disorder, dementia | Severe COVID-19 (oxygen therapy, MV/IMV, multi-organ failure/ECMO) Mortality | Severe disease (O2 treatment needed, original scale = 3–7)*: OS with nasal prongs, facial mask, noninvasive ventilation, invasive ventilation, multi-organ failure/ ECMO  *original scale = 3–7 ( 3 (O2 with nasal prongs), 4 (O2 with facial mask), 5 (noninvasive ventilaion), 6 (invasive ventilation), 7 (multi-organ failure/ ECMO) |
| Shin[118] | 2021 | South Korea | Autoimmune disorder, inflammatory arthritis, connective tissue disease | Severe COVID-19 (oxygen therapy, ICU admission, IMV, or mortality) Mortality | Requirement of oxygen therapy, ICU admission, application of invasive ventilation, or death) |
| Song[85] | 2021 | South Korea | Age, sex, BMI, diabetes, hypertension, HF, asthma, COPD, malignancy, dementia, chronic cardiac disease, CKD | Severe COVID-19 (IMV, multi-organ failure, ECMO, and mortality) Mortality | IMV, multi-organ failure, ECMO, and death |
| Wang[86] | 2021 | South Korea | Age, dementia, diabetes, hypertension, CKD | Mortality | COVID-related mortality |
| Yang[117] | 2021 | South Korea | Lung cancer | Severe COVID-19 (oxygen therapy, ICU admission, and MV, or ECMO, and mortality) | Severe COVID-19 was defined as cases with the need for oxygen therapy, prompt care in the ICU, and MV, or ECMO treatment in addition to patients who died after a confirmed COVID-19 diagnosis |
| Yang[111] | 2021 | South Korea | Asthma, allergic rhinitis, atopic dermatitis | Severe COVID-19 (ICU admission, IMV, or mortality) | Severe clinical outcomes comprised ICU admission, administration of invasive ventilation, or death of patients who tested positive for SARS-CoV-2 |
| Yoo[114] | 2021 | South Korea | NAFLD | Severe COVID-19 (oxygen therapy, MV, ICU admission, or mortality) Mortality | Severe clinical COVID-19 illnesses defined by requirement of oxygen therapy, administration of MV, ICU admission, COVID-19-related death |
| Yun[87] | 2020 | South Korea | Age, sex, hypertension, COPD, diabetes, CbVD, psychiatric disease, CCI score | Severe COVID-19 (MV, ECMO, or mortality) | Risk of critical condition or death relative to mild or severe condition. If a patient received oxygen therapy (by nasal cannula or mask), they were classified as a “severe” case. If a patient required a ventilator for breathing or if there was a claim history of ECMO use, they were classified as a “critical” case |
| Huang[88] | 2022 | Taiwan | Older age, CAD, CbVD, CKD | Mortality | NR |
| Naorungroj[89] | 2023 | Thailand | Age | Mortality | NR |
| Do[90] | 2023 | Vietnam | Age, diabetes | Mortality | NR |

Abbreviations: ADL, activities of daily living; AF, atrial fibrillation; AKI, acute kidney injury; ARDS, acute respiratory distress syndrome; BMI, body mass index; CAD, coronary artery disease; CbVD, cerebrovascualr disease; CCI, craniocervical instability; CHD, coronary heart disease; CHF, congestive heart failure; CKD, chronic kidney disease; CLD, chronic liver disease; COPD, chronic obstructive pulmonary disease; CPD, cephalopelvic disproportion; CRRT, continuous renal replacement therapy; CSS, clinical severity score; CVD, cardiovascular disease; ECMO, extracorporeal membrane oxygenation; ESRD, end-stage renal disease; FiO2, fraction of inspired oxygen; GCA, giant cell arteritis; GI, gastrointestinal; HBV, hepatitis B virus; HF, heart failure; HFNC, high-flow nasal cannula; ICD, International Classification of Diseases; ICU, intensive care unit; IHD, ischemic heart disease; IMV, intermittent mandatory ventilation; IPPV, Intermittent Positive Pressure Ventilation; JIA, juvenile inflammatory arthritis; MI, myocardial infarction; MV, mandatory ventilation; NAFLD, non-alcoholic fatty liver disease; NIPPV, noninvasive positive pressure ventilation; NOAF, new-onset atrial fibrillation; NR, not reported; OCD, obsessive compulsive disorder; PAD, peripheral arterial disease; PaO2, partial pressure of oxygen in the blood; RA, rheumatoid arthritis; PVD, peripheral vascular disease; RR, respiratory rate; RT-PCR, reverse transcription polymerase chain reaction; SLE, systemic lupus erythematosus; SpO2, oxygen saturation; TIA, transient ischemic attack; VTE, venous thromboembolism.

# References

1. Bhatia KS, Sritharan HP, Ciofani J, et al. Association of hypertension with mortality in patients hospitalised with COVID-19. Open Heart **2021**; 8: e001853.

2. Davis R, Bein K, Burrows J, et al. Clinical characteristics and predictors for hospitalisation during the initial phases of the Delta variant COVID-19 outbreak in Sydney, Australia. Emerg Med Australas **2023**; 35:34-40.

3. Ellis RJ, Moffatt CR, Aaron LT, et al. Factors associated with hospitalisations and deaths of residential aged care residents with COVID-19 during the Omicron (BA.1) wave in Queensland. Med J Aust **2023**; 218:174-9.

4. Liu B, Spokes P, He W, Kaldor J. High risk groups for severe COVID-19 in a whole of population cohort in Australia. BMC Infect Dis **2021**; 21:685.

5. Muleme M, McNamara BJ, Ampt FH, et al. Severity of COVID-19 among Residents in Aged Care Facilities in Victoria, Australia: A Retrospective Cohort Study Comparing the Delta and Omicron Epidemic Periods. J Am Med Dir Assoc **2023**; 24:434-40.e5.

6. Shiel E, Miyakis S, Tennant E, et al. Clinical characteristics and outcomes of COVID-19 in a low-prevalence, well resourced setting, Sydney, Australia. Intern Med J **2021**; 51:1605-13.

7. Wang J, Choy KW, Lim HY, Ho P. Laboratory markers of severity across three COVID-19 outbreaks in Australia: has Omicron and vaccinations changed disease presentation? Intern Emerg Med **2023**; 18:43-52.

8. Chung GK-K, Chan S-M, Chan Y-H, et al. Differential Impacts of Multimorbidity on COVID-19 Severity across the Socioeconomic Ladder in Hong Kong: A Syndemic Perspective. Int J Environ Res Public Health **2021**; 18:8168.

9. Fan FSY, Yip TCF, Yiu B, et al. Neurological diseases and risk of mortality in patients with COVID-19 and SARS: a territory-wide study in Hong Kong. J Neurol Neurosurg Psychiatry **2021**; 92:1356-8.

10. Teoh JY, Yip TC, Lui GC, et al. Risks of AKI and Major Adverse Clinical Outcomes in Patients with Severe Acute Respiratory Syndrome or Coronavirus Disease 2019. J Am Soc Nephrol **2021**; 32:961-71.

11. Wong JY, Cheung JK, Lin Y, et al. Intrinsic and effective severity of COVID-19 cases infected with the ancestral strain and Omicron BA.2 variant in Hong Kong. medRxiv **2023**:2023.02.13.23285848.

12. Yip TC, Wong VW, Lui GC, et al. Current and Past Infections of HBV Do Not Increase Mortality in Patients With COVID-19. Hepatology **2021**; 74:1750-65.

13. Zhou J, Lakhani I, Chou O, et al. Clinical characteristics, risk factors and outcomes of cancer patients with COVID-19: A population-based study. Cancer Med **2023**; 12:287-96.

14. Zhou J, Tse G, Lee S, et al. Identifying main and interaction effects of risk factors to predict intensive care admission in patients hospitalized with COVID-19: a retrospective cohort study in Hong Kong. medRxiv **2020**:2020.06.30.20143651.

15. Zhou J, Tse G, Lee S, et al. Renin-angiotensin system blockers and mortality in COVID-19: a territory-wide study from Hong Kong. medRxiv **2020**:2020.12.21.20248645.

16. Fukushima K, Yamada Y, Fujiwara S, et al. Development of a Risk Prediction Score to Identify High-Risk Groups for the Critical Coronavirus Disease 2019 (COVID-19) in Japan. Jpn J Infect Dis **2021**; 74:344-51.

17. Fukushima T, Chubachi S, Namkoong H, et al. Clinical significance of prediabetes, undiagnosed diabetes and diagnosed diabetes on critical outcomes in COVID-19: Integrative analysis from the Japan COVID-19 task force. Diabetes Obes Metab **2023**; 25:144-55.

18. Ito H, Sugimoto T, Ogihara Y, et al. Clinical characteristics and the risk of hospitalization of patients with coronavirus disease 2019 quarantined in a designated hotel in Japan. PLoS One **2023**; 18:e0280291.

19. Matsushita Y, Yokoyama T, Hayakawa K, et al. We Should Pay More Attention to Sex Differences to Predict the Risk of Severe COVID-19: Men Have the Same Risk of Worse Prognosis as Women More Than 10 Years Older. J Epidemiol **2023**; 33:38-44.

20. Miyashita K, Hozumi H, Furuhashi K, et al. Changes in the characteristics and outcomes of COVID-19 patients from the early pandemic to the delta variant epidemic: a nationwide population-based study. Emerg Microbes Infect **2023**; 12:2155250.

21. Muto Y, Awano N, Inomata M, et al. Predictive model for the development of critical coronavirus disease 2019 and its risk factors among patients in Japan. Respir Investig **2021**; 59:804-9.

22. Ninomiya T, Otsubo K, Hoshino T, et al. Risk factors for disease progression in Japanese patients with COVID-19 with no or mild symptoms on admission. BMC Infect Dis **2021**; 21:850.

23. Nishida Y, Mita T, Hiki M, Matsushita Y, Naito T, Watada H. Retrospective Study on the Effects of Glucose Abnormality on COVID-19 Outcomes in Japan. Diabetes Ther **2022**; 13:325-39.

24. Nishimura M, Asai K, Tabuchi T, et al. Association of combustible cigarettes and heated tobacco products use with SARS-CoV-2 infection and severe COVID-19 in Japan: a JASTIS 2022 cross-sectional study. Sci Rep **2023**; 13:1120.

25. Numaguchi R, Kurajoh M, Hiura Y, et al. Glycated hemoglobin level on admission associated with progression to severe disease in hospitalized patients with non-severe coronavirus disease 2019. J Diabetes Investig **2022**; 13:1779-87.

26. Okauchi Y, Matsuno K, Nishida T, et al. Obesity, glucose intolerance, advanced age, and lymphocytopenia are independent risk factors for oxygen requirement in Japanese patients with Coronavirus disease 2019 (COVID-19). Endocr J **2021**; 68:849-56.

27. Otoshi R, Hagiwara E, Kitayama T, et al. Clinical characteristics of Japanese patients with moderate to severe COVID-19. J Infect Chemother **2021**; 27:895-901.

28. Sakamoto Y, Satoh K, Tanaka T, et al. Risk factors associated with hospitalization in patients with asymptomatic or mild COVID-19 in public accommodation facilities in Tokyo. J Infect Chemother **2022**; 28:1439-44.

29. Sato R, Matsuzawa Y, Ogawa H, et al. Chronic kidney disease and clinical outcomes in patients with COVID-19 in Japan. Clin Exp Nephrol **2022**; 26:974-81.

30. Takeyama M, Yachi S, Nishimoto Y, et al. Mortality-associated Risk Factors in Hospitalized COVID-19 Patients in Japan: Findings of the CLOT-COVID Study. J Epidemiol **2023**; 33:150-7.

31. Tanaka K, Zha L, Kitamura T, et al. Characteristics and outcomes of older patients with coronavirus disease 2019 in Japan. Geriatr Gerontol Int **2021**; 21:629-35.

32. Yamada G, Hayakawa K, Matsunaga N, et al. Predicting respiratory failure for COVID-19 patients in Japan: a simple clinical score for evaluating the need for hospitalisation. Epidemiol Infect **2021**; 149:e175.

33. Jefferies S, French N, Gilkison C, et al. COVID-19 in New Zealand and the impact of the national response: a descriptive epidemiological study. Lancet Public Health **2020**; 5:e612-e23.

34. Anlacan VMM, Piamonte BLC, Sy MCC, Villanueva Iii EQ, Jamora RDG, Espiritu AI. Clinical Outcomes of Older Persons and Persons with Dementia Admitted for Coronavirus Disease 2019: Findings from the Philippine CORONA Study. Dement Geriatr Cogn Disord **2022**; 51:485-98.

35. Espiritu AI, Sy MCC, Anlacan VMM, Jamora RDG. COVID-19 outcomes of 10,881 patients: retrospective study of neurological symptoms and associated manifestations (Philippine CORONA Study). J Neural Transm (Vienna) **2021**; 128:1687-703.

36. Koh H, Moh AMC, Yeoh E, et al. Diabetes predicts severity of COVID-19 infection in a retrospective cohort: A mediatory role of the inflammatory biomarker C-reactive protein. J Med Virol **2021**; 93:3023-32.

37. Lim JP, Low KYH, Lin NJJ, et al. Predictors for development of critical illness amongst older adults with COVID-19: Beyond age to age-associated factors. Arch Gerontol Geriatr **2021**; 94:104331.

38. Ngiam JN, Liong TS, Chew NWS, et al. Serum creatinine to absolute lymphocyte count ratio effectively risk stratifies patients who require intensive care in hospitalized patients with coronavirus disease 2019. Medicine (Baltimore) **2022**; 101:e30755.

39. Tan HY, Yeo M, Tay XY, et al. Predictive performance of emergency department-specific variables on COVID-19 pneumonia. Singapore Med J **2022**; 63:715-22.

40. Tee LY, Alhamid SM, Tan JL, et al. COVID-19 and Undiagnosed Pre-diabetes or Diabetes Mellitus Among International Migrant Workers in Singapore. Front Public Health **2020**; 8:584249.

41. Bae S, Kim Y, Hwang S, Kwon KT, Chang HH, Kim SW. New Scoring System for Predicting Mortality in Patients with COVID-19. Yonsei Med J **2021**; 62:806-13.

42. Byeon KH, Kim DW, Kim J, Choi BY, Choi B, Cho KD. Factors affecting the survival of early COVID-19 patients in South Korea: An observational study based on the Korean National Health Insurance big data. Int J Infect Dis **2021**; 105:588-94.

43. Chang MC, Hwang JM, Jeon JH, Kwak SG, Park D, Moon JS. Fasting Plasma Glucose Level Independently Predicts the Mortality of Patients with Coronavirus Disease 2019 Infection: A Multicenter, Retrospective Cohort Study. Endocrinol Metab (Seoul) **2020**; 35:595-601.

44. Chang MC, Park YK, Kim BO, Park D. Risk factors for disease progression in COVID-19 patients. BMC Infect Dis **2020**; 20:445.

45. Chang Y, Jeon J, Song TJ, Kim J. Association of triglyceride-glucose index with prognosis of COVID-19: A population-based study. J Infect Public Health **2022**; 15:837-44.

46. Chang Y, Jeon J, Song TJ, Kim J. Association between the fatty liver index and the risk of severe complications in COVID-19 patients: a nationwide retrospective cohort study. BMC Infect Dis **2022**; 22:384.

47. Cho SI, Yoon S, Lee HJ. Impact of comorbidity burden on mortality in patients with COVID-19 using the Korean health insurance database. Sci Rep **2021**; 11:6375.

48. Cho SY, Park SS, Song MK, Bae YY, Lee DG, Kim DW. Prognosis Score System to Predict Survival for COVID-19 Cases: a Korean Nationwide Cohort Study. J Med Internet Res **2021**; 23:e26257.

49. Choi YJ, Park JY, Lee HS, et al. Variable effects of underlying diseases on the prognosis of patients with COVID-19. PLoS One **2021**; 16:e0254258.

50. Chung SM, Lee YY, Ha E, et al. The Risk of Diabetes on Clinical Outcomes in Patients with Coronavirus Disease 2019: A Retrospective Cohort Study. Diabetes Metab J **2020**; 44:405-13.

51. Her AY, Bhak Y, Jun EJ, et al. A Clinical Risk Score to Predict In-hospital Mortality from COVID-19 in South Korea. J Korean Med Sci **2021**; 36:e108.

52. Huh K, Ji W, Kang M, et al. Association of prescribed medications with the risk of COVID-19 infection and severity among adults in South Korea. Int J Infect Dis **2021**; 104:7-14.

53. Hwang J, Ryu HS, Kim HA, Hyun M, Lee JY, Yi HA. Prognostic Factors of COVID-19 Infection in Elderly Patients: A Multicenter Study. J Clin Med **2020**; 9:3932.

54. Hwang JM, Kim JH, Park JS, Chang MC, Park D. Neurological diseases as mortality predictive factors for patients with COVID-19: a retrospective cohort study. Neurol Sci **2020**; 41:2317-24.

55. Jang JG, Hur J, Choi EY, Hong KS, Lee W, Ahn JH. Prognostic Factors for Severe Coronavirus Disease 2019 in Daegu, Korea. J Korean Med Sci **2020**; 35:e209.

56. Jang SY, Seon JY, Eun BL, et al. Risk Factors of Outcomes of COVID-19 Patients in Korea: Focus on Early Symptoms. J Korean Med Sci **2021**; 36:e132.

57. Kang IS, Kong KA. Body mass index and severity/fatality from coronavirus disease 2019: A nationwide epidemiological study in Korea. PLoS One **2021**; 16:e0253640.

58. Kang SH, Kim SW, Kim AY, Cho KH, Park JW, Do JY. Association between Chronic Kidney Disease or Acute Kidney Injury and Clinical Outcomes in COVID-19 Patients. J Korean Med Sci **2020**; 35:e434.

59. Kim DW, Byeon KH, Kim J, Cho KD, Lee N. The Correlation of Comorbidities on the Mortality in Patients with COVID-19: an Observational Study Based on the Korean National Health Insurance Big Data. J Korean Med Sci **2020**; 35:e243.

60. Kim HS, Kang M, Kang G. Renin-angiotensin system modulators and other risk factors in COVID-19 patients with hypertension: a Korean perspective. BMC Infect Dis **2021**; 21:175.

61. Kim J, Heo N, Kang H. Sex-Based Differences in Outcomes of Coronavirus Disease 2019 (COVID-19) in Korea. Asian Nurs Res (Korean Soc Nurs Sci) **2022**; 16:224-30.

62. Kim SR, Nam SH, Kim YR. Risk Factors on the Progression to Clinical Outcomes of COVID-19 Patients in South Korea: Using National Data. Int J Environ Res Public Health **2020**; 17:8847.

63. Lee J, Kim AR, Kang SW, et al. Protracted course of SARS-CoV-2 pneumonia in moderately to severely immunocompromised patients. Clin Exp Med **2023**; 23:2255-64.

64. Lee JH, Kim YC, Cho SH, et al. Effect of sex hormones on coronavirus disease 2019: an analysis of 5,061 laboratory-confirmed cases in South Korea. Menopause **2020**; 27:1376-81.

65. Lee JY, Nam BH, Kim M, et al. A risk scoring system to predict progression to severe pneumonia in patients with Covid-19. Sci Rep **2022**; 12:5390.

66. Lee SH, Park SY, Seon JY, et al. Intensive Care Unit Capacity and Its Associated Risk Factors During the COVID-19 Surge in the Republic of Korea: Analysis Using Nationwide Health Claims Data. Risk Manag Healthc Policy **2020**; 13:2571-81.

67. Lee SI, Chung C, Park D, Kang DH, Ju YR, Lee JE. The Influence of Sex on Characteristics and Outcomes of Coronavirus-19 Patients: A Retrospective Cohort Study. J Clin Med **2023**; 12.

68. Lee SC, Son KJ, Han CH, Jung JY, Park SC. Impact of comorbid asthma on severity of coronavirus disease (COVID-19). Sci Rep **2020**; 10:21805.

69. Lee SC, Son KJ, Han CH, Park SC, Jung JY. Impact of COPD on COVID-19 prognosis: A nationwide population-based study in South Korea. Sci Rep **2021**; 11:3735.

70. Lee SG, Park GU, Moon YR, Sung K. Clinical Characteristics and Risk Factors for Fatality and Severity in Patients with Coronavirus Disease in Korea: A Nationwide Population-Based Retrospective Study Using the Korean Health Insurance Review and Assessment Service (HIRA) Database. Int J Environ Res Public Health **2020**; 17.

71. Lee YR, Kang MK, Song JE, et al. Clinical outcomes of coronavirus disease 2019 in patients with pre-existing liver diseases: A multicenter study in South Korea. Clin Mol Hepatol **2020**; 26:562-76.

72. Lee YW, Seon JY, Lee SH, Oh IH. COVID-19 Disease Burden Related to Social Vulnerability and Comorbidities: Challenges to Tuberculosis Control. Int J Environ Res Public Health **2022**; 19: 3597.

73. Lim JK, Park B, Park J, et al. Impact of Computed Tomography-Quantified Emphysema Score on Clinical Outcome in Patients with COVID-19. Int J Gen Med **2021**; 14:3327-33.

74. Moon HJ, Kim K, Kang EK, Yang HJ, Lee E. Prediction of COVID-19-related Mortality and 30-Day and 60-Day Survival Probabilities Using a Nomogram. J Korean Med Sci **2021**; 36:e248.

75. Moon SS, Lee K, Park J, Yun S, Lee YS, Lee DS. Clinical Characteristics and Mortality Predictors of COVID-19 Patients Hospitalized at Nationally-Designated Treatment Hospitals. J Korean Med Sci **2020**; 35:e328.

76. Oh H, Kim R, Chung W. Sex-Specific Association between Underlying Diseases and the Severity and Mortality Due to COVID-19 Infection: A Retrospective Observational Cohort Analysis of Clinical Epidemiological Information Collected by the Korea Disease Control and Prevention Agency. Healthcare (Basel) **2022**; 10: 1846.

77. Oh TK, Song IA. Impact of coronavirus disease-2019 on chronic respiratory disease in South Korea: an NHIS COVID-19 database cohort study. BMC Pulm Med **2021**; 21:12.

78. Oh TK, Choi JW, Song IA. Socioeconomic disparity and the risk of contracting COVID-19 in South Korea: an NHIS-COVID-19 database cohort study. BMC Public Health **2021**; 21:144.

79. Paek JH, Kim Y, Park WY, et al. Severe acute kidney injury in COVID-19 patients is associated with in-hospital mortality. PLoS One **2020**; 15:e0243528.

80. Park BE, Lee JH, Park HK, et al. Impact of Cardiovascular Risk Factors and Cardiovascular Diseases on Outcomes in Patients Hospitalized with COVID-19 in Daegu Metropolitan City. J Korean Med Sci **2021**; 36:e15.

81. Park JG, Kang MK, Lee YR, et al. Fibrosis-4 index as a predictor for mortality in hospitalised patients with COVID-19: a retrospective multicentre cohort study. BMJ Open **2020**; 10:e041989.

82. Seon JY, Kim S, Hong M, Lim MK, Oh IH. Risk of COVID-19 diagnosis and death in patients with mental illness: a cohort study. Epidemiol Psychiatr Sci **2021**; 30:e68.

83. Seong GM, Baek AR, Baek MS, et al. Comparison of Clinical Characteristics and Outcomes of Younger and Elderly Patients with Severe COVID-19 in Korea: A Retrospective Multicenter Study. J Pers Med **2021**; 11:1258.

84. Shin EK, Choi HY, Hayes N. The anatomy of COVID-19 comorbidity networks among hospitalized Korean patients. Epidemiol Health **2021**; 43:e2021035.

85. Song J, Park DW, Cha JH, et al. Clinical course and risk factors of fatal adverse outcomes in COVID-19 patients in Korea: a nationwide retrospective cohort study. Sci Rep **2021**; 11:10066.

86. Wang SM, Park SH, Kim NY, et al. Association between Dementia and Clinical Outcome after COVID-19: A Nationwide Cohort Study with Propensity Score Matched Control in South Korea. Psychiatry Investig **2021**; 18:523-9.

87. Yun K, Lee JS, Kim EY, Chandra H, Oh BL, Oh J. Severe COVID-19 Illness: Risk Factors and Its Burden on Critical Care Resources. Front Med (Lausanne) **2020**; 7:583060.

88. Huang AC, Lin SM, Chiu TH, et al. Comparison of Clinical Characteristics and Outcomes of Hospitalized Patients Infected with the D614G Strain or Alpha Variant of COVID-19 in Taiwan: A Multi-Center Cohort Study. Int J Med Sci **2022**; 19:1912-9.

89. Naorungroj T, Viarasilpa T, Tongyoo S, et al. Characteristics, outcomes, and risk factors for in-hospital mortality of COVID-19 patients: A retrospective study in Thailand. Front Med (Lausanne) **2022**; 9:1061955.

90. Do TV, Manabe T, Vu GV, et al. Clinical characteristics and mortality risk among critically ill patients with COVID-19 owing to the B.1.617.2 (Delta) variant in Vietnam: A retrospective observational study. PLoS One **2023**; 18:e0279713.

91. Nojiri S, Irie Y, Kanamori R, Naito T, Nishizaki Y. Mortality Prediction of COVID-19 in Hospitalized Patients Using the 2020 Diagnosis Procedure Combination Administrative Database of Japan. Intern Med **2023**; 62:201-13.

92. Cho Y, Cho Y, Choi HJ, et al. The effect of BMI on COVID-19 outcomes among older patients in South Korea: a nationwide retrospective cohort study. Ann Med **2021**; 53:1292-301.

93. Her AY, Bhak Y, Jun EJ, et al. Sex-specific difference of in-hospital mortality from COVID-19 in South Korea. PLoS One **2022**; 17:e0262861.

94. Kim DH, Park HC, Cho A, et al. Age-adjusted Charlson comorbidity index score is the best predictor for severe clinical outcome in the hospitalized patients with COVID-19 infection. Medicine (Baltimore) **2021**; 100:e25900.

95. Park HC, Kim DH, Cho A, et al. Clinical outcomes of initially asymptomatic patients with COVID-19: a Korean nationwide cohort study. Ann Med **2021**; 53:357-64.

96. Lee H, Chubachi S, Namkoong H, et al. Effects of mild obesity on outcomes in Japanese patients with COVID-19: a nationwide consortium to investigate COVID-19 host genetics. Nutr Diabetes **2022**; 12:38.

97. Ogihara Y, Yachi S, Takeyama M, et al. Influence of obesity on incidence of thrombosis and disease severity in patients with COVID-19: From the CLOT-COVID study. J Cardiol **2023**; 81:105-10.

98. Espiritu AI, Reyes NGD, Leochico CFD, et al. Body mass index and its association with COVID-19 clinical outcomes: Findings from the Philippine CORONA study. Clin Nutr ESPEN **2022**; 49:402-10.

99. Kim NH, Kim KJ, Choi J, Kim SG. Metabolically unhealthy individuals, either with obesity or not, have a higher risk of critical coronavirus disease 2019 outcomes than metabolically healthy individuals without obesity. Metabolism **2022**; 128:154894.

100. Kim SY, Yoo DM, Min C, Wee JH, Kim JH, Choi HG. Analysis of Mortality and Morbidity in COVID-19 Patients with Obesity Using Clinical Epidemiological Data from the Korean Center for Disease Control & Prevention. Int J Environ Res Public Health **2020**; 17:9336.

101. Yip TC, Lui GC, Wong VW, et al. Liver injury is independently associated with adverse clinical outcomes in patients with COVID-19. Gut **2021**; 70:733-42.

102. Kurahara Y, Kobayashi T, Shintani S, et al. Clinical characteristics of COVID-19 in Osaka, Japan: Comparison of the first-third waves with the fourth wave. Respir Investig **2021**; 59:810-8.

103. Espiritu AI, Sucaldito M, Ona DID, et al. Clinical outcomes in COVID-19 among patients with hypertension in the Philippine CORONA Study. Eur J Med Res **2023**; 28:62.

104. Ji W, Huh K, Kang M, et al. Effect of Underlying Comorbidities on the Infection and Severity of COVID-19 in Korea: a Nationwide Case-Control Study. J Korean Med Sci **2020**; 35:e237.

105. Kang SH, Lee DH, Han KD, et al. Hypertension, renin-angiotensin-aldosterone-system-blocking agents, and COVID-19. Clin Hypertens **2021**; 27:11.

106. Kim J, Park SH, Kim JM. Effect of Comorbidities on the Infection Rate and Severity of COVID-19: Nationwide Cohort Study With Propensity Score Matching. JMIR Public Health Surveill **2022**; 8:e35025.

107. Espiritu AI, Chiu HHC, Sy MCC, Anlacan VMM, Jamora RDG. The outcomes of patients with diabetes mellitus in The Philippine CORONA Study. Sci Rep **2021**; 11:24436.

108. Kim MK, Jeon JH, Kim SW, et al. The Clinical Characteristics and Outcomes of Patients with Moderate-to-Severe Coronavirus Disease 2019 Infection and Diabetes in Daegu, South Korea. Diabetes Metab J **2020**; 44:602-13.

109. Moon SJ, Rhee EJ, Jung JH, et al. Independent Impact of Diabetes on the Severity of Coronavirus Disease 2019 in 5,307 Patients in South Korea: A Nationwide Cohort Study. Diabetes Metab J **2020**; 44:737-46.

110. Jung Y, Wee JH, Kim JH, Choi HG. The Effects of Previous Asthma and COPD on the Susceptibility to and Severity of COVID-19: A Nationwide Cohort Study in South Korea. J Clin Med **2021**; 10:4626.

111. Yang JM, Koh HY, Moon SY, et al. Allergic disorders and susceptibility to and severity of COVID-19: A nationwide cohort study. J Allergy Clin Immunol **2020**; 146:790-8.

112. Sano T, Matsumoto S, Ikeda T, et al. New-Onset Atrial Fibrillation in Patients With Coronavirus Disease 2019 (COVID-19) and Cardiovascular Disease　- Insights From the CLAVIS-COVID Registry. Circ J **2022**; 86:1237-44.

113. Jeon D, Son M, Choi J. Impact of liver cirrhosis on the clinical outcomes of patients with COVID-19: a nationwide cohort study of Korea. Korean J Intern Med **2021**; 36:1092-101.

114. Yoo HW, Jin HY, Yon DK, et al. Non-alcoholic Fatty Liver Disease and COVID-19 Susceptibility and Outcomes: a Korean Nationwide Cohort. J Korean Med Sci **2021**; 36:e291.

115. Espiritu AI, Larrazabal RB, Jr., Sy MCC, Villanueva EQ, 3rd, Anlacan VMM, Jamora RDG. Outcomes and Risk Factors of Patients With COVID-19 and Cancer (ONCORONA): Findings from The Philippine CORONA Study. Front Oncol **2022**; 12:857076.

116. Baek MS, Lee MT, Kim WY, Choi JC, Jung SY. COVID-19-related outcomes in immunocompromised patients: A nationwide study in Korea. PLoS One **2021**; 16:e0257641.

117. Yang B, Choi H, Lee SK, et al. Risk of Coronavirus Disease 2019 Occurrence, Severe Presentation, and Mortality in Patients with Lung Cancer. Cancer Res Treat **2021**; 53:678-84.

118. Shin YH, Shin JI, Moon SY, et al. Autoimmune inflammatory rheumatic diseases and COVID-19 outcomes in South Korea: a nationwide cohort study. Lancet Rheumatol **2021**; 3:e698-e706.

119. Lee DY, Cho J, You SC, et al. Risk of Mortality in Elderly Coronavirus Disease 2019 Patients With Mental Health Disorders: A Nationwide Retrospective Study in South Korea. Am J Geriatr Psychiatry **2020**; 28:1308-16.

120. Jeon H-L, Kwon JS, Park S-H, Shin J-Y. Association of mental disorders with SARS-CoV-2 infection and severe health outcomes: a nationwide cohort study. medRxiv **2020**:2020.08.05.20169201.

121. Lee SW, Yang JM, Moon SY, et al. Association between mental illness and COVID-19 susceptibility and clinical outcomes in South Korea: a nationwide cohort study. Lancet Psychiatry **2020**; 7:1025-31.

122. Shoji K, Tsuzuki S, Akiyama T, et al. Clinical Characteristics and Outcomes of Coronavirus Disease 2019 (COVID-19) in Pregnant Women: A Propensity Score-Matched Analysis of Data From the COVID-19 Registry Japan. Clin Infect Dis **2022**; 75:e397-e402.

123. Larrazabal RB, Jr., Chiu HHC, Palileo-Villanueva LAM. Outcomes of nutritionally at-risk Coronavirus Disease 2019 (COVID 19) patients admitted in a tertiary government hospital: A follow-up study of the MalnutriCoV study. Clin Nutr ESPEN **2021**; 43:239-44.

124. Australian Government Department of Health and Aged Care. You are hereHome Recommendations Adults aged ≥18 years are recommended to receive COVID-19 vaccine. Available at: <https://immunisationhandbook.health.gov.au/recommendations/adults-aged-18-years-are-recommended-to-receive-covid-19-vaccine>. Accessed October 22 2024.

125. Australian Government Department of Health and Aged Care. ATAGI statement on the administration of COVID-19 vaccines in 2024. Available at: <https://www.health.gov.au/resources/publications/atagi-statement-on-the-administration-of-covid-19-vaccines-in-2024>. Accessed October 22 2024.

126. Centre for Health Protection. Consensus Interim Recommendations on Use of COVID-19 Vaccines in Hong Kong (as of 17 July 2024). Available at: <https://www.chp.gov.hk/files/pdf/consensus_interim_recommendations_on_use_of_covid19_vaccines_in_hong_kong_17jul.pdf>. Accessed October 22 2024.

127. Centre for Health Protection. How many doses of COVID-19 vaccine are recommended for me? Available at: <https://www.chp.gov.hk/files/pdf/poster_recommend_dose.pdf>. Accessed October 22 2024.

128. Ministry of Health Labour and Welfare. About the COVID-19 vaccine. Available at: <https://www.mhlw.go.jp/stf/seisakunitsuite/bunya/vaccine_00184.html>. Accessed October 22 2024.

129. Health New Zealand. About COVID-19. Available at: <https://info.health.nz/conditions-treatments/infectious-diseases/covid-19/about-covid-19#covid-19-prevention-695>. Accessed October 22 2024.

130. Health New Zealand. COVID-19 vaccines additional doses Available at: <https://info.health.nz/immunisations/vaccines-aotearoa/covid-19-vaccines/covid-19-vaccine-boosters>. Accessed October 22 2024.

131. Ministry of Health. COVID-19 vaccination. Available at: <https://www.moh.gov.sg/covid-19/vaccination>. Accessed October 22 2024.

132. Ministry of Health. List of medical conditions for which persons are considered medically vulnerable to severe covid-19 and recommended a second booster dose. Available at: <https://www.moh.gov.sg/docs/librariesprovider5/default-document-library/annexa9750bbc24d74226a05f0ca74ca322ba.pdf>. Accessed October 22 2024.

133. Korea Disease Control and Prevention Agency. Additional vaccinations for high-risk groups for COVID-19, including immunocompromised individuals, to begin on April 15 (Friday, March 22). Available at: <https://www.kdca.go.kr/board/board.es?mid=a20501010000&bid=0015&list_no=724871&cg_code=&act=view&nPage=18&newsField>=. Accessed October 22 2024.

134. Taiwan Centers for Disease Control. The second dose of the COVID-19 XBB vaccine will be administered from now on: Add 1 more dose of the new crown vaccine for those over 65 years old, and the protection will not expire. Available at: <https://www.cdc.gov.tw/Bulletin/Detail/LJBhkv0fvVtJxG0bzqP0vw?typeid=9>. Accessed October 22 2024.

135. Việt Nam News. Việt Nam issues new COVID-19 vaccine guidance amid uncertain global pandemic situation. Available at: <https://vietnamnews.vn/society/1655537/viet-nam-issues-new-covid-19-vaccine-guidance-amid-uncertain-global-pandemic-situation.html>. Accessed October 22 2024.
